# How Effective Have Thirty Years of Internationally Driven Conservation and Development Efforts Been in Madagascar?

**DOI:** 10.1371/journal.pone.0161115

**Published:** 2016-08-17

**Authors:** Patrick O. Waeber, Lucienne Wilmé, Jean-Roger Mercier, Christian Camara, Porter P. Lowry

**Affiliations:** 1 Forest Management and Development, Department of Environmental Sciences, Swiss Federal Institute of Technology Zurich, Zurich, Switzerland; 2 Madagascar Wildlife Conservation, Ambatondrazaka, Madagascar; 3 University of Antananarivo, School of Agronomy, Water and Forest Department, Antananarivo, Madagascar; 4 Missouri Botanical Garden, Madagascar Research & Conservation Program, Antananarivo, Madagascar; 5 Independent Researcher, *Saint-André*, France; 6 Missouri Botanical Garden, Africa and Madagascar Program, St. Louis, Missouri, United States of America; 7 Institut de systématique, évolution, et biodiversité, Unité mixte de recherche 7205, Centre national de la recherche scientifique/Muséum national d’Histoire Naturelle/École pratique des hautes études, Université Pierre et Marie Curie, Sorbonne Universités, Paris, France; University of Massachusetts Amherst, UNITED STATES

## Abstract

Conservation and development are intricately linked. The international donor community has long provided aid to tropical countries in an effort to alleviate poverty and conserve biodiversity. While hundreds of millions of $ have been invested in over 500 environmental-based projects in Madagascar during the period covered by a series of National Environmental Action Plans (1993–2008) and the protected areas network has expanded threefold, deforestation remains unchecked and none of the eight Millennium Development Goals (MDGs) established for 2000–2015 were likely be met. Efforts to achieve sustainable development had failed to reduce poverty or deliver progress toward any of the MDGs. Cross-sectorial policy adjustments are needed that (i) enable and catalyze Madagascar’s capacities rather than deepening dependency on external actors such as the World Bank, the International Monetary Fund and donor countries, and that (ii) deliver improvements to the livelihoods and wellbeing of the country’s rural poor.

## Introduction

Human activities and demands on Earth’s natural capital have been steadily increasing and several interlinked planetary boundaries have now been crossed, causing fundamental and potentially catastrophic environmental change [1]. Terrestrial and aquatic biodiversity has been on a steady decline and is projected to continue its downward spiral at an increasing rate [2]. Biodiversity conservation has been a centerpiece of environmental politics and policy over the last several decades, and while conservation focused on a ‘nature exclusive’ pathway for decades, it has gradually evolved towards a ‘nature including humans’ approach [3]. In the same period, many transnational agencies and international NGOs and economists have claimed that biodiversity can only be preserved by adopting the idea of natural capital as a way to increase the visibility and the perceived economic value of nature [4]. A conviction grew out of this that by monetizing and commodifying nature for the goods and service it provides, and by requiring compensation for the destructive exploitation of these resources, sustainable use can be encouraged and stimulated [5].

Current levels and patterns of human consumption are undermining our planet’s environmental resource base and exacerbating inequalities. According to the latest Oxfam report on the 2015 World Economic Forum held in Davos, Switzerland, 50% of the world’s wealth is held by the richest 1% of the population [6]. Efforts to bridge this inequality gap include, inter alia, economic-based concepts that seek to incentivize the sustainable use and conservation of the biological resources and ecological functions that provide valuable services and benefits, such as market based ecosystem services and payments for ecosystem services.

Since the publication of the Brundtland Commission’s report entitled “Our Common Future”, global concepts such as sustainable development and approaches such as integrated conservation and development projects (ICDP) have been used widely in an attempt to bridge the conservation-poverty gap. 2015 represented a key transition from the Millennium Development Goals (MDGs, Table A in S1 File) to the Sustainable Development Goals (SDGs) and marked a growing consensus on a shared Post-2015 agenda, especially that developed by the High-Level Panel on Post-2015.

In an effort to examine the impact and effectiveness of conservation and development efforts that have been undertaken at both the international and national level, we present a case study using Madagascar. We chose this country because it is internationally recognized for its unique, globally important biodiversity [7,8] and is often held up as an example of how responsible environmental governance can address a complex and difficult set of inter-linked social and environmental problems [9]. Since independence, Madagascar has been the beneficiary of several hundred million $ of aid and development support [10–13]. It was also one of the first countries to develop a National Environmental Action Plan (NEAP), which was done in two stages: preparation and formal adoption (1990), followed by three successive 5-year environmental management projects (1993–2008) [14]. In parallel, Madagascar entered into various international agreements such as the Convention on Biodiversity (CBD) and the Convention on International Trade in Endangered Species of Wild Fauna and Flora (CITES), linking national efforts to global goals and strategies. In this context, we consider two related questions in this paper: (1) has the effort in Madagascar, involving the government, local institutions and international partners, and often applying tools and principles founded in economics, been effective in conserving the country’s natural environment?; and (2) did the overall sustainable development situation improve in Madagascar during the period covered by the MDGs (i.e., through the end of 2015)? In order to address these questions, we summarize information on several key MDG indicators and considered national and international efforts undertaken with institutional support and financial assistance, as well as the role and effectiveness of Madagascar’s protected areas network. Data and information were collected and collated from open source databases (e.g., IUCN, World Bank data report).

## Methods

We collected and collated data and information from a search of published and unpublished secondary sources, in French and English, conducted in August to October 2015 using the open source catalogues of Google Scholar and Scopus: Key search terms (“forest conservation”, “ecosystem services”, “sustainable development”, “forest cover”) were used in a boolean combination with the key terms “global”, “Madagascar”, and “tropical”. We also used our own sources on Madagascar’s biodiversity based on the Noe4D database [15] comprising ca. 10,000 references and 50,000 georeferenced records primarily documenting the endemic vertebrate fauna. Noe4D was also consulted to assess research efforts driven by Malagasy institutions and individuals (cf. S1 Fig).

Publicly available databases and annual reports of the International Union for Conservation of Nature (IUCN), World Bank, United Nations Development Program (UNDP), Organization for Economic Co-operation and Development (OECD), and Food and Agricultural Organization (FAO) were consulted to access data and information relating to: a) indicators of the Millennium Development Goals (MDGs) (cf. Tables A–S in S1 File), b) institutions involved in Madagascar’s conservation and development projects (the operators), and c) aid funding provided by bi- and multi-lateral donors to the Malagasy Government to support the implementation of these projects. AidData.org was consulted for information on projects and funding from 1990 to 2012 (the last year for which data were available), using the following key words: “environment”, “conservation”, and “Madagascar”. These were compared with figures provided by the Madagascar Government on aid funding available at stpca-primature.gov.mg (cf. Table T in S1 File for original data).

## Results

### MDGs 1–6: poverty, education, gender equality, health

**Fig 1:**
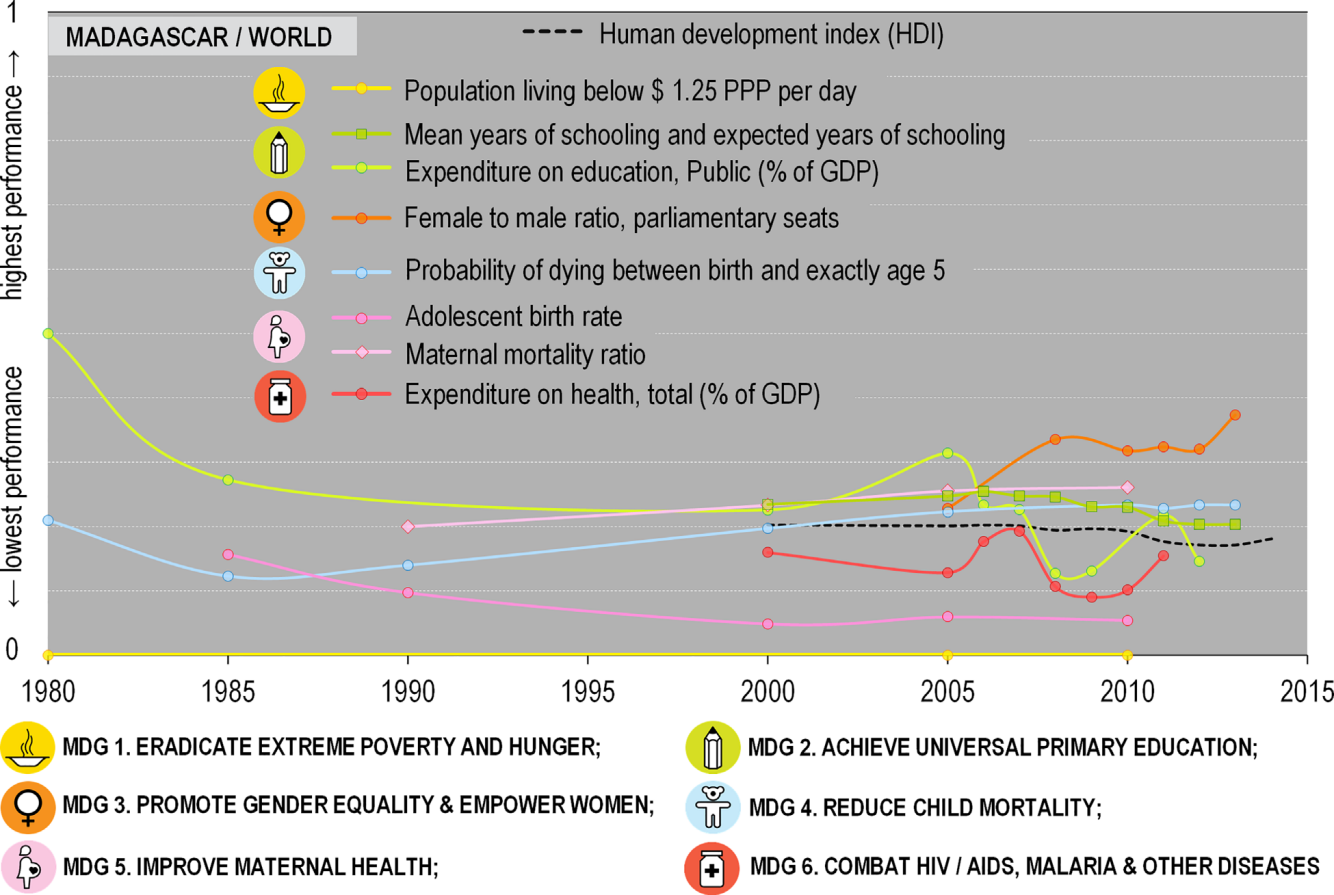
Evolution of a selection of key indicators representing the six first Millennium Development Goals (MDGs). In order to allow comparison between the indicators, all have been normalized to one. The graphs read as a relative performance to scores of other countries worldwide (Tables B–S in S1 File), or other African countries (S1 Fig).

According to the 2014 MDG Monitor [16], Madagascar was on track during the period 2002–2006 to achieve its goals. This trend was reversed during the subsequent period of political instability (2009–2013) and its aftermath (Fig 1, S2 File). By 2015, Madagascar had the highest proportion of the working population living below the international poverty level of $1.25 per day (cf. MDG 1, “eradicate extreme poverty & hunger”, see also Tables D,E in S1 File) of any country in the world. Despite the Education for All Action Plan, the relative level of investment in education declined throughout the MDG period and especially during the political crisis, compared to global levels, with a drop from over 0.3 to a level around or below 0.2 (Fig 1). Likewise, mean and expected years of schooling (cf. Tables F,G in S1 File for MDG 2 indicator, with MDG 2 being “achieve universal primary education”) experienced a steep decline in 2010 compared to 187 other countries, and has stagnated since.

The objective of MDG 4 is to reduce child mortality by 66.7% by the end of 2015. While the probability of dying between birth and age 5 (MDG 4) was almost cut in half between 2000 and 2013 (from 109‰ to 58‰), it decreased only slightly since 2010 and Madagascar remained among the lowest performing of the 194 countries considered (Fig 1, Fig B in S2 File). The MDG 5 objective is to improve maternal health. Its six indicators have been stagnant for the most part. The maternal health in Madagascar has not improved compared to other countries: in 2010 it ranked 175th among 185 for adolescent birth rates and the maternal mortality rate was 240 deaths of women per 100,000 live births (135th of 180 globally and 14th of 51 African countries, Fig A in S2 File), considered a ‘low human development’ value. Expenditures on health (MDG 6, “combat HIV/AIDS, malaria and other diseases”) increased significantly in 2006, when Madagascar ranked 33rd of 188 countries, followed by a decrease and then a sudden increase in 2011, when the country ranked 28th (Fig 1). Madagascar’s Human Development Index (HDI), a composite measure of development based on life expectancy at birth, knowledge and education, and standard of living, has steadily decreased throughout the period of the MDGs (Fig 1).

### MDG 7: environment

**Fig 2:**
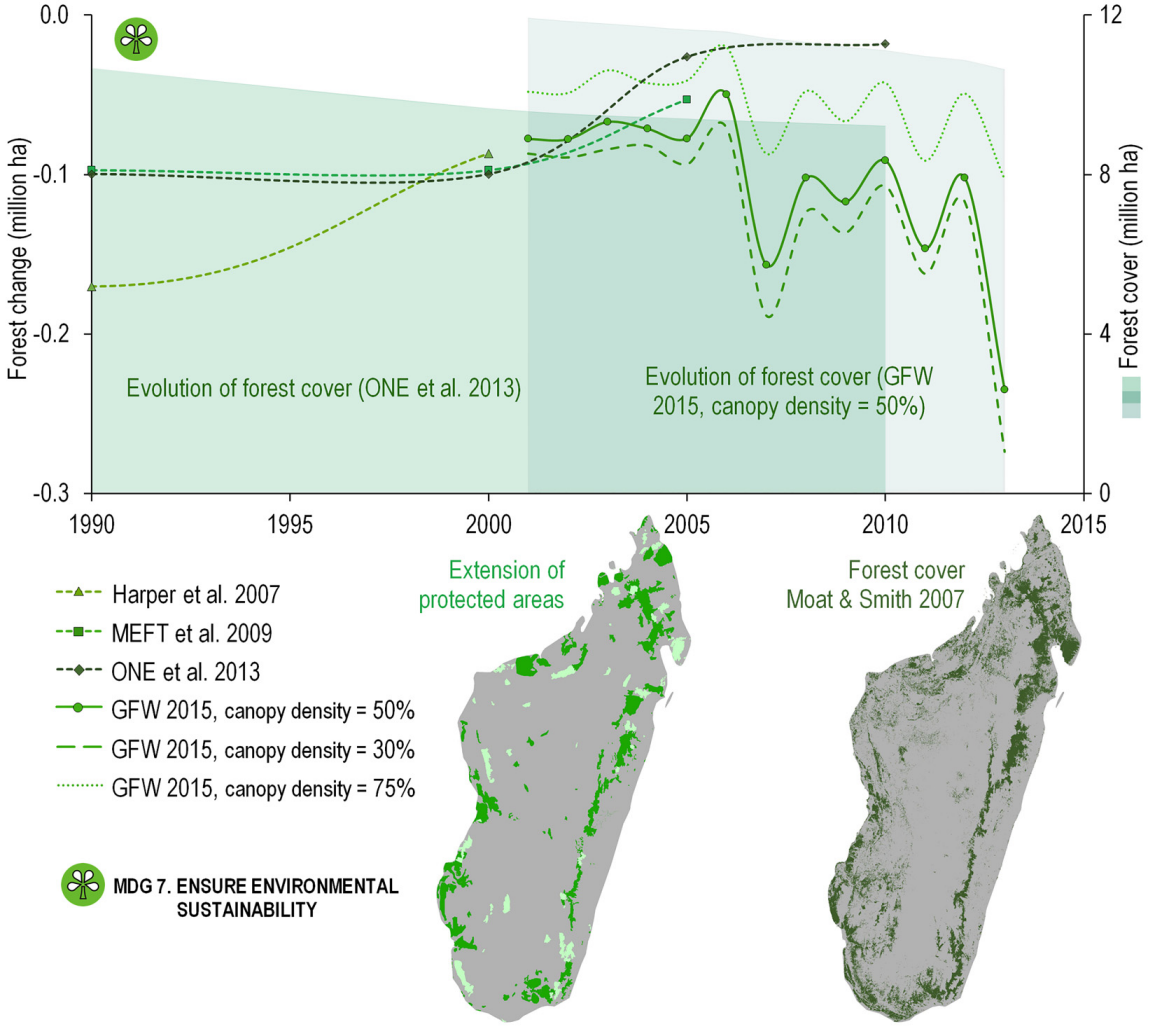
Forest cover and extension of protected areas as two indicators for the MDG 7 (environment), representing the outcomes of conservation efforts over the last 25 years. The forest loss over time according to four different publicly available sources [18–21]. Left Madagascar map: The light green are the early parks and reserves until the 2000s, while the dark green are the extension of the system of Madagascar protected areas (SAMP). After the political crisis from 2009 to 2013, the ‘*Code des Aires Protégées*’ was revised under the ‘*Refonte du Code des Aires Protégées*’ (N. 2015–005). This was supplemented with an updated environmental charter, ‘*Charte de l’Environnement Malagasy Actualisé*’ (N. 2015–003). Further, the ratification and finalization of 74 new protected areas into permanent Protected Areas is covering now a total area of 70,815 km^2^.

The 7 indicators for MDG 7 aim to assess environmental sustainability. The majority of Madagascar’s endemic floral and faunal species are found in forest ecosystems [17,18]. Successive National Evaluations [19,20] show a steady decrease in the loss of forest cover since the 1990s, and especially since 2000 (Fig 2), whereas estimates by the Global Forest Watch (GFW) in 2015 [21] clearly show an increase in forest loss after 2006, independent of canopy density (Fig 2). We have used a canopy density of 50% (Fig 2) because using a density of 75% leads to an overestimate of the area occupied by humid forest, while a 30% threshold underestimates the extent of dry forests. There is a discrepancy of more than an order of magnitude between the increasing deforestation rates indicated in the GFW and the decreasing deforestation rates estimated by ONE et al. studies [20,21] (cf. years 2006 and onwards, Fig 2).

### MDG 8: partnership

**Fig 3:**
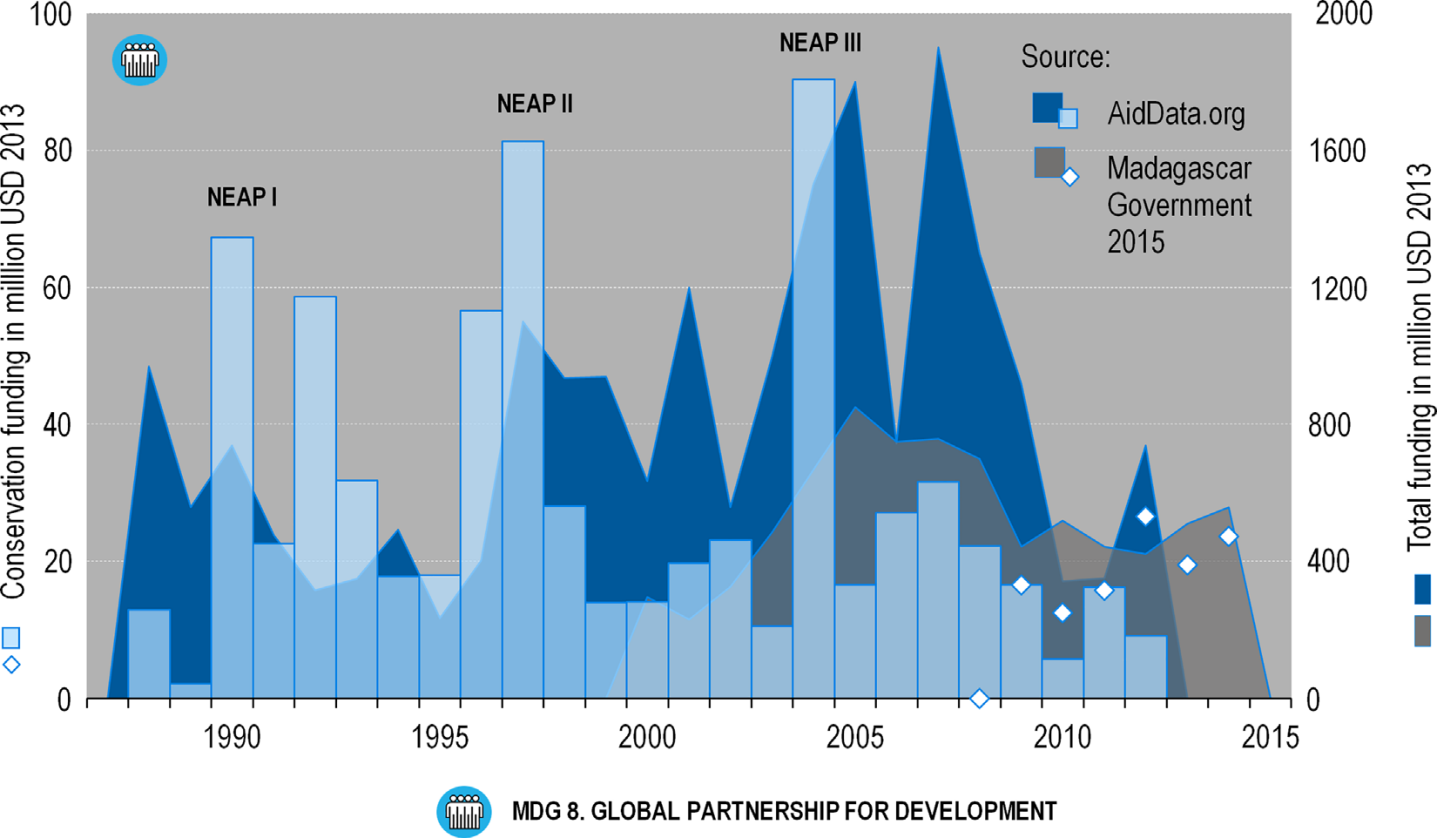
Aid funding for Madagascar during the period 1988 to 2014; total funding (right Y axis) and funding for environmental projects (left Y axis). Data based on AidData.org and Madagascar Government (Table T in S1 File).

In the late 1980s and early 1990s, the NGOs operating in Madagascar increased in number and level of activity as support from bilateral and multilateral donors and financial institutions expanded [9]. Since 1990, aid flowing into Madagascar accounted for more than $19 billion (in 2013 dollars). At the start of the NEAP in 1990 only a few NGOs were promoting conservation in partnership with the Water and Forest authorities, but their numbers grew rapidly as the NEAP progressed through phases 1–3. According to AidData.com some $700 million flowed into Madagascar for conservation programs from 1990 to 2012, representing 3.7% of total foreign assistance (Fig 3), which averaged ca. $500,000 per year between 2009 and 2014 [21] that qualified as Official Development Aid (ODA) as defined by the OECD [22]. Madagascar’s ODA constitutes up to 70% to total development financing [23], of which 4.8% was allocated to conservation programs on average between 2009 and 2012, ranging from 3.0% in 2010 to 7.2% in 2012 (Fig 3).

## Discussion

According to some economists, external aid creates dependencies, fosters corruption, and increases poverty [10,24]. Our analysis of the MDG indicators suggests that Madagascar likely failed to achieve any of the Millennium Development Goals and targets. Some have said that Africa (including Madagascar) would never have been able to meet the goals given the arbitrary “success” and “failure” targets and levels that were set [25]. In an analysis of state-donor relations in the context of conservation in Madagascar, Horning [10] has shown that Madagascar’s poor performance can also be attributed to competition among donors, who defend their own interest. The author demonstrated that the interplay between foreign aid agencies and donors, on the one hand, and the government of Madagascar’s efforts to interest a diversity of donors, on the other, is mutualistic, and unless Madagascar defines its own development goals, the situation will remain unchanged [10]. Further, Madagascar’s political crisis (2009–2013) unquestionably hampered the process and put the country on an even more difficult track, despite the fact that the initial MDG assessment [16] seemed promising. Gore and collaborators [26] state that the social mechanisms used by informal institutions such as village committees may actually increase accountability (and hence reduce corruption) and may be more efficient than new, additional layers of state governance, such as those imposed during the NEAP phase. Important constraints on the decentralization of public service delivery are especially salient in remote areas [27]. Interestingly, even during the crisis, aid variously estimated at $421–519 million [23] or $342–918 million (AidData.org) per year continued (Fig 3), despite the fact that the ‘transitional government’ was not recognized as legitimate by the international donor community. Starting in 2012, the prospect of presidential elections coincided with a significant increase in aid money of more than $337 million (cf. AidData.org in Fig 3). Presidential and legislative elections were held in December 2013 and a new government was appointed at the end of January 2014. It is interesting to note that figures provided by the Madagascar Government in 2015 [23] suggest a higher portion of international aid going to support the environment sector during this period than indicated by the available data based on the OECD (cf. Fig 3). This discrepancy becomes even more evident when comparing these numbers with the figures on deforestation provided by ONE et al. in 2013 [20] (MDG 7, Fig 2) in the light of carbon PES (payments for environmental services). Conservation projects based on PES and REDD (reducing emissions through deforestation and degradation) goals being spearheaded by NGOs, such as Conservation International (CI), Wildlife Conservation Society (WCS) and World Wildlife Fund for Nature (WWF) in eastern Madagascar, are increasingly being promoted, though when compared to the 500+ environmental projects carried out between 1990 and 2012, they still represent a small minority [12,28,29]. Another interesting aspect are the spikes in health expenditures in 2006 and 2013 (Fig 1), which coincided with presidential elections, a pattern similar to those observed in logging of precious hardwoods, in particular increased illegal rosewood trafficking to finance electoral campaigns [30,31].

Global biodiversity is decreasing and will continue to decline over the 21st century [32]. The IUCN Red List highlights species whose evaluation shows they are at the greatest risk of extinction, providing a valuable tool for conservation [33,34]. Starting in the early 1990s, assessments of species submitted to the IUCN Red List require “p. 72 … the rationale for listing, supported by data on range size, population size and trend, distribution, habitat preferences, altitude, threats and conservation actions in place or needed” [34]. Examples of global assessment are provided by Butchart et al. in 2004 [35] for birds and Nori & Loyola in 2015 [36] for amphibians, and Brummitt et al. [37] examined a sample of plants, all with the goal of facilitating the integration of information on threatened species in the design of protected areas. For Madagascar, 3921 native taxa have been assessed, including 1036 plants (representing no more than 8% of the 12,000–14,000 plant species in Madagascar) and 2885 animals (2077 vertebrates, including 268 amphibians, 392 reptiles, 244 birds and 245 mammals; cf. Table U in S1 File). Many new species are being described in each group [17], the rate of which may, as in other tropical countries, approximate that at which conservation assessments are being done [33]. Globally, revisions of assessments resulting in a change in threat category are mostly the result of improved knowledge or updated taxonomy [33], a situation also found in Madagascar, as exemplified by the 22 species of lemurs recognized in the early 1980s vs. more than 100 species today [38]. Although the IUCN Red List is a valuable conservation tool, it cannot been used to assess MDG 7.

In human-caused habitat degradations and conversions, some species are lost others suffer from defaunation [39]. Using a large sample of species, Boyd et al. [40] showed that site protection is the cornerstone for assuring the survival of ca. 99% of threatened species. Since the majority of the terrestrial biodiversity endemic to Madagascar occurs in forests and other woody vegetation types [17], several major indicators for MDG 7 are relevant for monitoring environmental sustainability (MDG 7), such as the proportion of land area covered by forest (target 7.1, cf. Fig 2) and the proportion of terrestrial and marine areas protected (target 7.6, cf. Fig 2), given that protected areas (PAs) are one of the main tools available to conserve threatened species by reducing or halting habitat loss, fragmentation, overexploitation and other anthropogenic pressures [41]. However, with our current level of knowledge of species richness among Malagasy plants and animals (see above), it is not possible to assess the proportion of species threatened with extinction (target 7.7), so we have opted to use forest cover as a proxy for biodiversity health, an approach we regard as sufficiently robust because forest cover trends clearly impact biodiversity [42]. The establishment of PAs is widely assumed to reduce deforestation, but in Madagascar the rate was only marginally lower in parks and reserves during the three decades of intervention, due to factors such as a significant increase in illegal logging within PAs, especially of rosewood, which continues unabated [30] (Fig 2), mirroring a global pattern [43].

Drivers of deforestation at the global scale are generally well understood [44]. Madagascar’s unique biodiversity has been in the focus of the international conservation and research communities since the late 1980s, and many studies have explored the causes and drivers of deforestation, among which slash-and-burn agriculture (‘tavy’) is generally regarded as one of the most important [18,45]. However, despite a growing body of knowledge resulting from major research efforts [46], no alternatives have been adopted and implemented at a scale anywhere near what is needed to have an appreciable impact on deforestation at a national level. Recent work proposes a more bottom-up strategy to complement current knowledge (i.e., a local vs. scientific approach [47]) to address the continuing decrease in the area of forest in Madagascar despite more than three decades of national and international conservation programs. A comparison of studies based on satellite images [48] VS. [49] clearly shows that deforestation over time is not only leading to a change in land cover but also to increased fragmentation, thus compounding the negative impacts on biodiversity [50–52].

Early in the political crisis that began in 2009, the international donor community froze all financial aid, maintaining only emergency humanitarian support to address the most urgent needs. Acute malnourishment has long been prevalent, especially in southern Madagascar, which often faces extended periods of drought, exacerbating food shortages and leading on occasion to major famines. A much more prevalent, albeit less visible, problem is chronic malnourishment, in particular insufficiencies in vitamins and microminerals, which are especially problematic during the first years of child development and can stunt brain capacity [53]. Chronic malnourishment affects some 50% of Madagascar’s children [54] and impacts the country’s economic performance, GNI and GDP, not only affecting youth physically but mentally [55]. Children with reduced brain capacity perform at a lower level, requiring on average two years longer to finish a primary school degree. The dropout rate is also much higher, significantly so since before the crisis, although this is also due in part to economic reasons since families everywhere in Madagascar are finding it increasingly difficult to pay for teacher salaries and school fees in the face of rising food prices and/or food insecurity [29,54], a situation that is being compounded by the consequences of climate change.

Globally, areas of high poverty coincide with those that show a high vulnerability to climate change, such as Haiti [56]. Ensuring environmental sustainability requires consideration of risks stemming from climate change, which will impact many MDGs (e.g., MDG 1, “eradicate extreme poverty and hunger”; MDG 4, “reduce child mortality”; or MDG 7, “ensure environmental sustainability”) and possibly SDGs [57]. The frequency of tropical storms, droughts, floods, and insect infestations is increasing globally as is the predicted intensity of cyclonic events [58], which will intensify pressure on forests and the biodiversity they contain [59], and extended droughts will impact dry and arid ecosystems. Such extreme climatic events will also negatively impact humans and undermine development efforts [60,61]. For example, the consequences of the locust plague that hit the Sahel region in the early 2000s cost of over $500 million [62]. Madagascar has also experienced severe insect outbreaks, threatening the food base of several million people living in the southwest [62] and further increasing pressure on the region’s remaining natural ecosystems.

For more than two decades starting in the 1970s the World Bank advocated reduced allocations to higher education, which contributed to a decline in the quality of institutions of higher education, the consequences of which are still visible today [63]. According to Levine and Bolo [64], higher education was too costly, inequitable, and inefficient, and only contributed marginally to national development goals, a position that stood in contrast to that taken by African governments [63]. However, growing social and economic unrest and increased protest, often initiated from universities, led governments, including Madagascar’s, to abandon their earlier thinking and adopt the policy changes promoted by the World Bank [63]. For example, in the 1990s the Malagasy government imposed a hiring freeze to reduce expenditures in the higher education sectors [65], and while the students-to-teacher-ratio has improved, the quality of education has suffered and the inefficiency of the entire education system has worsened (over 30% of the total budget goes to administration, referred to as ‘technical support’) [65].

One consequence of the reduced capacity in Madagascar’s universities is the lack of ‘locally produced’ expertise. The conservation and development ‘operators’ that implement donor-funded projects have had to bring in technology, knowhow and staff, using a considerable portion of aid money (cf. ‘Assistance Technique’, which cost ca. $23 million from 2009–2014 [21]). Another consequence is that Malagasy universities can barely compete by international standards. The situation in Madagascar mirrors global conservation and development patterns, with national research institutions receiving so little funding that they have no choice but to engage in international research projects [66] that are led and driven by institutions mainly in Europe and the USA. Of 3942 publications on biodiversity from 1960 to 2015, the lead author of only 352 (8.9%) was based at a Malagasy institution (based on Noe4D [15], cf. S1 Fig), which is paralleled by vertebrate collections in natural history museums (all major 20th century inventory projects were led by institutions in Europe or the USA). This situation will impact Madagascar’s development for years to come, profoundly influencing the quality of education, which is far more important than time spent in schools, as evaluated under MDG 2 [67]. In recent years the World Bank has begun to change its policy, acknowledging the importance to higher education for development, which may lead to better support for Malagasy universities. The higher education system has recently been reorganized to align with that used internationally and new bachelors and masters level courses are now offered in fields such as sustainable development and environmental impact assessment.

Has the effort in Madagascar been effective in conserving the country’s natural environment? Nationally- and internationally-led efforts have produced more than a threefold increase in the area managed for conservation, which formally provides protection for natural habitats and populations of many IUCN red listed species but does not guarantee that the threats they face are reduced. Indeed, while legal protection empowers authorities to take action when the law is broken, deforestation and illegal exploitation are still impacting nearly all protected areas despite 30 years of intensive conservation efforts. Enforcement remains weak and inconsistent, a reflection of Madagascar’s rampant corruption at all levels (according to the Corruption Perception Index the country was ranked 85th of 180 in 2008, 133/175 in 2014, and 132/168 in 2015). Quantitative benchmarking of efforts to conserve biodiversity will require monitoring both forest loss and quality, coupled with expanded red listing and regular re-assessments based on updated data as habitats are further impacted.

Did the overall sustainable development situation improve in Madagascar during the period covered by the MDGs? By most measures, the country is faring worse than ever. The poverty and school dropout rates have increased, and most other indicators point in the same direction (Figs 1 and 2). While this may in part be due to the country’s latest political crisis, it has only exacerbated problems that have existed for decades and persist today. It is thus clear that while the significant investments made in Madagascar to address the twin challenges of sustainable development and biodiversity conservation have had positive benefits, they have failed to reverse long-standing trends and the country has fallen short on almost all of its Millennium Development Goals.

As the MDG process came to an end in December 2015 and stock was taken of progress toward its eight goals, the leaders of 190 countries committed to 17 new, ambitious Sustainable Development Goals (SDGs) that they hope will end global poverty, fight inequality and injustice, and abate climate change. Can the SDGs deliver success where the MDGs failed? Madagascar has experienced a political crisis almost every decade [30], which is likely to be at the core of the failure of governance. Unstable governments are often under frequent changes in the composition of parliament, and with it are decrease in transparency and -accountability [68,69]. Conservation is intricately intertwined with development, especially in biodiversity-rich tropical countries such as Madagascar, where a majority of the population lives in rural areas and depends directly on natural resources. Many policy adjustments have been made to address conservation and development issues jointly in Madagascar, but internationally-supported efforts have barely affected rural people. Yet these same poor, rural people are the ones profoundly altering the island’s landscapes to meet their basic, daily needs, and in the process they have the greatest overall impact on the country’s ecosystems and biodiversity. The formulation of policies to promote conservation and development goals and the identification of strategies and actions need to include and involve these key actors. Moreover, decision-making must avoid the disconnect between, on the one hand, adjusting national-level policy on biodiversity values on the basis of globally-developed economic principles and mechanisms (which are likely to have little meaning at the local level), and on the other hand, setting sectorial or cross-sectorial development targets, which are almost always driven by external donors and international operators but fail to take into account the complex, front-line interactions between humans and the many other biodiversity elements on which they so deeply depend. Many aspects of the situation found in Madagascar are shared with other biodiversity-rich but economically disadvantaged countries that face a similar array of challenges. As decisions are made on priority actions to meet the new Sustainable Development Goals, it would be instructive to take stock of whether progress was made over the last 15 years toward achieving the Millennium Development Goals and if not, why not.

## Supporting Information

S1 Fig Publications documenting the biodiversity of vertebrates in Noe4D. (PDF)

S1 File Information, data and sources considered to document Madagascar’s relative development performance: Official list of MDGs goals, targets and indicators (Table A). Human development index (HDI) values (Table B). HDI rank for each country per year (Table C). Percentage of the population living below the international poverty line $1.25 (in purchasing power parity terms) a day (Table D). Country rank for population living below $1.25 PPP per day (%) (Table E). Mean years of schooling and expected years of schooling (Table F). Country rank per year for Mean Years of Schooling and Expected Years of Schooling (Table G). Total public expenditure (current and capital) on education expressed as a percentage of GDP (Table H). Country rank per year for Expenditure on education, Public (% of GDP) (Table I). Percentage of parliamentary seats held by women expressed as a ratio of those held by men (Table J). Country rank per year for percentage of parliamentary seats held by women expressed as a ratio of those held by men (Table K). Probability of dying between birth and exactly age 5, expressed per 1,000 live births (Table L). Country rank per year for Probability of dying between birth and exactly age 5 (Table M). Adolescent birth rate (women aged 15–19 years) (births per 1,000 women ages 15–19) (Table N). Country rank per year for number of births to women ages 15–19 per 1,000 women ages 15–19 (Table O). Maternal mortality ratio (deaths of women per100,000 live births) (Table P). Country rank per year for maternal mortality ratio (Table Q). Current and capital spending on health (Table R). Country rank per year for total expenditure on health (Table S). Aid funding for Madagascar during the period 1988 to 2014 in millions of dollars (Table T). Madagascar native taxa assessed for the Red List (Table U).
(PDF)

S2 File MDG performance of Madagascar compared to the World, Africa, and Sub-Saharan Africa:. Relative performance of Madagascar compared to Africa (Fig A). Evolution of HDI and MDGs 1 to 6 values over time for Madagascar, mean values for the World, Sub-Saharan Africa, and minimum and maximum values (Fig B).
(PDF)
