# Supplementary material for: How Effective Have Thirty Years of Internationally Driven Conservation and Development Efforts Been in Madagascar?
Source: PLoS One. 2016 Aug 17;11(8):e0161115. doi: 10.1371/journal.pone.0161115 (PMC4988661; doi:10.1371/journal.pone.0161115)
Supplement: S1 Fig — (PDF) [file pone.0161115.s001.pdf]

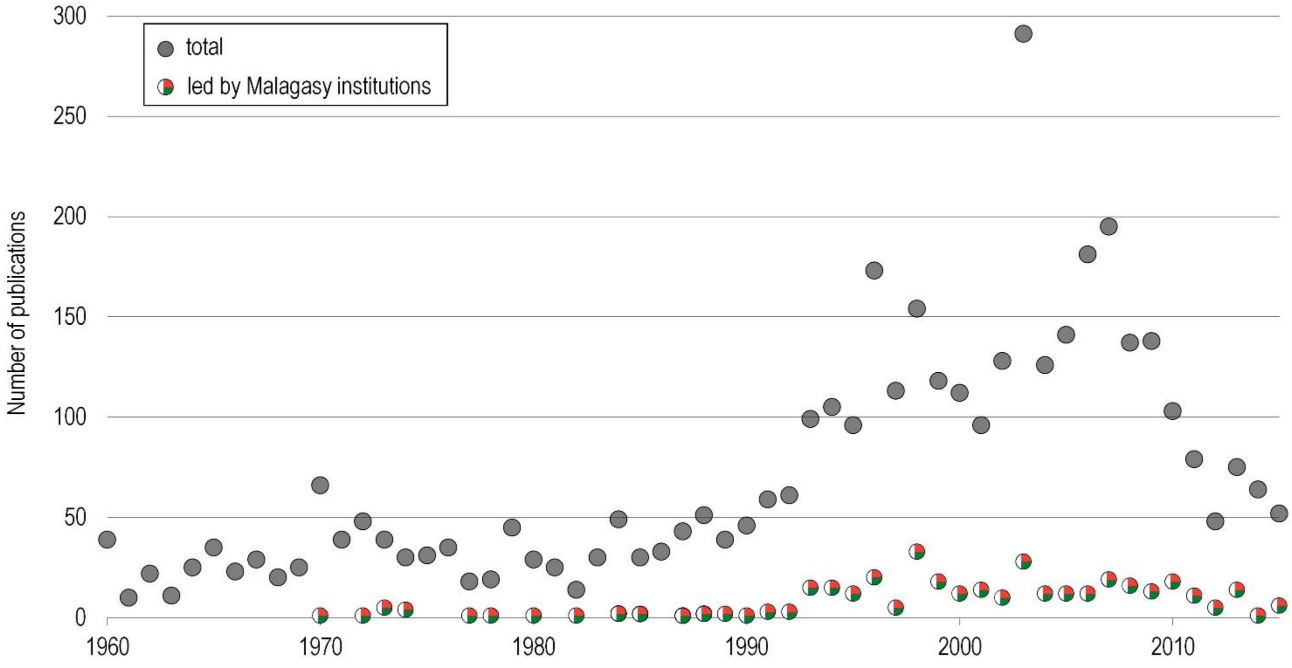

S1 Fig. Publication documenting the biodiversity of vertebrates in Noe4D.

(Wilmé L, Ravokatra M, Dolch R, Schuurman D, Mathieu E, Schuetz H, Waeber, PO. Toponyms for centers of endemism in Madagascar. Madag Conserv Dev. 2012; 7: 30–40.)
