# Supplementary material for: How Effective Have Thirty Years of Internationally Driven Conservation and Development Efforts Been in Madagascar?
Source: PLoS One. 2016 Aug 17;11(8):e0161115. doi: 10.1371/journal.pone.0161115 (PMC4988661; doi:10.1371/journal.pone.0161115)
Supplement: S1 File — Official list of MDGs goals, targets and indicators (Table A). Human development index (HDI) values (Table B). HDI rank for each country per year (Table C). Percentage of the population living below the international poverty line $1.25 (in purchasing power parity terms) a day (Table D). Country rank for population living below $1.25 PPP per day (%) (Table E). Mean years of schooling and expected years of schooling (Table F). Country rank per year for Mean Years of Schooling and Expected Years of Schooling (Table G). Total public expenditure (current and capital) on education expressed as a percentage of GDP (Table H). Country rank per year for Expenditure on education, Public (% of GDP) (Table I). Percentage of parliamentary seats held by women expressed as a ratio of those held by men (Table J). Country rank per year for percentage of parliamentary seats held by women expressed as a ratio of those held by men (Table K). Probability of dying between birth and exactly age 5, expressed per 1,000 live births (Table L). Country rank per year for Probability of dying between birth and exactly age 5 (Table M). Adolescent birth rate (women aged 15–19 years) (births per 1,000 women ages 15–19) (Table N). Country rank per year for number of births to women ages 15–19 per 1,000 women ages 15–19 (Table O). Maternal mortality ratio (deaths of women per100,000 live births) (Table P). Country rank per year for maternal mortality ratio (Table Q). Current and capital spending on health (Table R). Country rank per year for total expenditure on health (Table S). Aid funding for Madagascar during the period 1988 to 2014 in millions of dollars (Table T). Madagascar native taxa assessed for the Red List (Table U). (PDF) [file pone.0161115.s002.pdf]

S1 File. Information, data and sources considered to document Madagascar's relative development performance

Table A. Official list of MDGs goals, targets and indicators 2

Table B. Human development index (HDI) values 5

Table C. HDI rank for each country per year 9

Table D. Percentage of the population living below the international poverty line $1.25 (in purchasing power parity terms) a day 14

Table E. Country rank for population living below $1.25 PPP per day (%) 16

Table F. Mean years of schooling and expected years of schooling 20

Table G. Country rank per year for Mean Years of Schooling and Expected Years of Schooling 24

Table H. Total public expenditure (current and capital) on education expressed as a percentage of GDP 30

Table I. Country rank per year for Expenditure on education, Public (% of GDP) 34

Table J. Percentage of parliamentary seats held by women expressed as a ratio of those held by men 39

Table K. Country rank per year for percentage of parliamentary seats held by women expressed as a ratio of those held by men 43

Table L. Probability of dying between birth and exactly age 5, expressed per 1,000 live births 48

Table M. Country rank per year for Probability of dying between birth and exactly age 5 53

Table N. Adolescent birth rate (women aged 15–19 years) (births per 1,000 women ages 15–19) 58

Table O. Country rank per year for number of births to women ages 15–19 per 1,000 women ages 15–19 62

Table P. Maternal mortality ratio (deaths of women per100,000 live births) 68

Table Q. Country rank per year for maternal mortality ratio 72

Table R. Current and capital spending on health 78

Table S. Country rank per year for total expenditure on health 83

Table T. Aid funding for Madagascar during the period 1988 to 2014 in millions of dollars 88

Table U. Madagascar native taxa assessed for the Red List 89

Table A. Official list of MDGs goals, targets and indicators
(source of the table: http://mdgs.un.org/unsd/mdg/host.aspx?Content=indicators/officiallist.htm)
The chosen indicators for this study were based on available data for Madagascar; if data deficient, we did not consider an indicator for tracking evolution over the period 2000-2015

**Millennium Development Goals (MDGs)**	
**Goals and Targets (from the Millennium Declaration)**	**Indicators for monitoring progress**
**Goal 1: Eradicate extreme poverty and hunger**	
Target 1.A: Halve, between 1990 and 2015, the proportion of people whose income is less than one dollar a day	1.1 Proportion of population below $1.25 (PPP) per day<br>1.2 Poverty gap ratio<br>1.3 Share of poorest quintile in national consumption
Target 1.B: Achieve full and productive employment and decent work for all, including women and young people	1.4 Growth rate of GDP per person employed<br>1.5 Employment-to-population ratio<br>1.6 Proportion of employed people living below $1.25 (PPP) per day<br>1.7 Proportion of own-account and contributing family workers in total employment
Target 1.C: Halve, between 1990 and 2015, the proportion of people who suffer from hunger	1.8 Prevalence of underweight children under-five years of age<br>1.9 Proportion of population below minimum level of dietary energy consumption
**Goal 2: Achieve universal primary education**	
Target 2.A: Ensure that, by 2015, children everywhere, boys and girls alike, will be able to complete a full course of primary schooling	2.1 Net enrolment ratio in primary education<br>2.2 Proportion of pupils starting grade 1 who reach last grade of primary<br>2.3 Literacy rate of 15-24 year-olds, women and men
**Goal 3: Promote gender equality and empower women**	
Target 3.A: Eliminate gender disparity in primary and secondary education, preferably by 2005, and in all levels of education no later than 2015	3.1 Ratios of girls to boys in primary, secondary and tertiary education<br>3.2 Share of women in wage employment in the non-agricultural sector<br>3.3 Proportion of seats held by women in national parliament
**Goal 4: Reduce child mortality**	
Target 4.A: Reduce by two-thirds, between 1990 and 2015, the under-five mortality rate	4.1 Under-five mortality rate<br>4.2 Infant mortality rate<br>4.3 Proportion of 1 year-old children immunised against measles
**Goal 5: Improve maternal health**	
Target 5.A: Reduce by three quarters, between 1990 and 2015, the maternal mortality ratio	5.1 Maternal mortality ratio<br>5.2 Proportion of births attended by skilled health personnel
Target 5.B: Achieve, by 2015, universal access to reproductive health	5.3 Contraceptive prevalence rate<br>5.4 Adolescent birth rate<br>5.5 Antenatal care coverage (at least one visit and at least four visits)<br>5.6 Unmet need for family planning
**Goal 6: Combat HIV/AIDS, malaria and other diseases**	
Target 6.A: Have halted by 2015 and begun to reverse the spread of HIV/AIDS	6.1 HIV prevalence among population aged 15-24 years<br>6.2 Condom use at last high-risk sex<br>6.3 Proportion of population aged 15-24 years with comprehensive correct knowledge of HIV/AIDS<br>6.4 Ratio of school attendance of orphans to school attendance of non-orphans aged 10-14 years
Target 6.B: Achieve, by 2010, universal access to treatment for HIV/AIDS for all those who need it	6.5 Proportion of population with advanced HIV infection with access to antiretroviral drugs
Target 6.C: Have halted by 2015 and begun to reverse the incidence of malaria and other major diseases	6.6 Incidence and death rates associated with malaria

Millennium Development Goals (MDGs)	
**Goals and Targets (from the Millennium Declaration)**	**Indicators for monitoring progress**
	6.7 Proportion of children under 5 sleeping under insecticide-treated bednets<br>6.8 Proportion of children under 5 with fever who are treated with appropriate anti-malarial drugs<br>6.9 Incidence, prevalence and death rates associated with tuberculosis<br>6.10 Proportion of tuberculosis cases detected and cured under directly observed treatment short course
**Goal 7: Ensure environmental sustainability**	
Target 7.A: Integrate the principles of sustainable development into country policies and programmes and reverse the loss of environmental resources<br>Target 7.B: Reduce biodiversity loss, achieving, by 2010, a significant reduction in the rate of loss	7.1 Proportion of land area covered by forest<br>7.2 CO2 emissions, total, per capita and per $1 GDP (PPP)<br>7.3 Consumption of ozone-depleting substances<br>7.4 Proportion of fish stocks within safe biological limits<br>7.5 Proportion of total water resources used<br>7.6 Proportion of terrestrial and marine areas protected<br>7.7 Proportion of species threatened with extinction
Target 7.C: Halve, by 2015, the proportion of people without sustainable access to safe drinking water and basic sanitation	7.8 Proportion of population using an improved drinking water source<br>7.9 Proportion of population using an improved sanitation facility
Target 7.D: By 2020, to have achieved a significant improvement in the lives of at least 100 million slum dwellers	7.10 Proportion of urban population living in slums
**Goal 8: Develop a global partnership for development**	
Target 8.A: Develop further an open, rule-based, predictable, non-discriminatory trading and financial system<br><br>Includes a commitment to good governance, development and poverty reduction – both nationally and internationally<br><br>Target 8.B: Address the special needs of the least developed countries<br><br>Includes: tariff and quota free access for the least developed countries' exports; enhanced programme of debt relief for heavily indebted poor countries (HIPC) and cancellation of official bilateral debt; and more generous ODA for countries committed to poverty reduction<br><br>Target 8.C: Address the special needs of landlocked developing countries and small island developing States (through the Programme of Action for the Sustainable Development of Small Island Developing States and the outcome of the twenty-second special session of the General Assembly)<br><br>Target 8.D: Deal comprehensively with the debt problems of developing countries through national and international measures in order to make debt sustainable in the long term	*Some of the indicators listed below are monitored separately for the least developed countries (LDCs), Africa, landlocked developing countries and small island developing States.*<br><br>Official development assistance (ODA)<br>8.1 Net ODA, total and to the least developed countries, as percentage of OECD/DAC donors' gross national income<br>8.2 Proportion of total bilateral, sector-allocable ODA of OECD/DAC donors to basic social services (basic education, primary health care, nutrition, safe water and sanitation)<br>8.3 Proportion of bilateral official development assistance of OECD/DAC donors that is untied<br>8.4 ODA received in landlocked developing countries as a proportion of their gross national incomes<br>8.5 ODA received in small island developing States as a proportion of their gross national incomes<br>Market access<br>8.6 Proportion of total developed country imports (by value and excluding arms) from developing countries and least developed countries, admitted free of duty<br>8.7 Average tariffs imposed by developed countries on agricultural products and textiles and clothing from developing countries<br>8.8 Agricultural support estimate for OECD countries as a percentage of their gross domestic product<br>8.9 Proportion of ODA provided to help build trade capacity<br><br>Debt sustainability<br>8.10 Total number of countries that have reached their HIPC decision points and number that have reached their HIPC completion points (cumulative)<br>8.11 Debt relief committed under HIPC and MDRI Initiatives

Millennium Development Goals (MDGs)	
**Goals and Targets (from the Millennium Declaration)**	**Indicators for monitoring progress**
	8.12 Debt service as a percentage of exports of goods and services
Target 8.E: In cooperation with pharmaceutical companies, provide access to affordable essential drugs in developing countries	8.13 Proportion of population with access to affordable essential drugs on a sustainable basis
Target 8.F: In cooperation with the private sector, make available the benefits of new technologies, especially information and communications	8.14 Fixed-telephone subscriptions per 100 inhabitants<br>8.15 Mobile-cellular subscriptions per 100 inhabitants<br>8.16 Internet users per 100 inhabitants

The Millennium Development Goals and targets come from the Millennium Declaration, signed by 189 countries, including 147 heads of State and Government, in September 2000 (http://www.un.org/millennium/declaration/ares552e.htm) and from further agreement by member states at the 2005 World Summit (Resolution adopted by the General Assembly - A/RES/60/1, http://www.un.org/Docs/journal/asp/ws.asp?m=A/RES/60/1). The goals and targets are interrelated and should be seen as a whole. They represent a partnership between the developed countries and the developing countries "to create an environment – at the national and global levels alike – which is conducive to development and the elimination of poverty".

Human development index (HDI) value
A composite index measuring average achievement in three basic dimensions of human development—a long and healthy life, knowledge and a decent standard of living. See Technical note 1 for details on how the HDI is calculated.
Source: HDRO calculations based on data from UNDESA (2013a), Barro and Lee (2013), UNESCO Institute for Statistics (2013b), United Nations Statistics Division (2014), World Bank (2014) and IMF (2014).
Data in the tables are those available to the Human Development Report Office as of 15 November, 2013, and Human Development Report 2015 Work for Human Development (http://hdr.undp.org/en/2015-report)

Table B. Human development index (HDI) values

HDI Rank	Countries	2000	2005	2006	2007	2008	2009	2010	2011	2012	2013	2014
169	Afghanistan	0.341	0.396	0.406	0.416	0.430	0.437	0.453	0.458	0.466	0.468	0.465
95	Albania	0.655	0.689	0.694	0.699	0.703	0.705	0.708	0.714	0.714	0.716	0.733
93	Algeria	0.634	0.675	0.680	0.686	0.695	0.703	0.709	0.715	0.715	0.717	0.736
37	Andorra							0.832	0.831	0.830	0.830	0.845
149	Angola	0.377	0.446	0.465	0.478	0.490	0.491	0.504	0.521	0.524	0.526	0.532
61	Antigua and Barbuda							0.778	0.772	0.773	0.774	0.783
49	Argentina	0.753	0.758	0.766	0.771	0.777	0.789	0.799	0.804	0.806	0.808	0.836
87	Armenia	0.648	0.693	0.707	0.721	0.722	0.717	0.720	0.724	0.728	0.730	0.733
2	Australia	0.898	0.912	0.915	0.918	0.922	0.924	0.926	0.928	0.931	0.933	0.935
21	Austria	0.835	0.851	0.857	0.861	0.868	0.870	0.877	0.879	0.880	0.881	0.885
76	Azerbaijan	0.639	0.686	0.703	0.715	0.724	0.736	0.743	0.743	0.745	0.747	0.751
51	Bahamas	0.766	0.787	0.789	0.791	0.791	0.788	0.788	0.789	0.788	0.789	0.790
44	Bahrain	0.784	0.811	0.808	0.809	0.810	0.811	0.812	0.812	0.813	0.815	0.824
142	Bangladesh	0.453	0.494	0.502	0.510	0.515	0.527	0.539	0.549	0.554	0.558	0.570
59	Barbados	0.745	0.761	0.764	0.771	0.776	0.782	0.779	0.780	0.776	0.776	0.785
53	Belarus		0.725	0.738	0.751	0.764	0.775	0.779	0.784	0.785	0.786	0.798
21	Belgium	0.873	0.865	0.868	0.871	0.873	0.873	0.877	0.880	0.880	0.881	0.890
84	Belize	0.675	0.710	0.708	0.707	0.710	0.712	0.714	0.717	0.731	0.732	0.715
165	Benin	0.391	0.432	0.439	0.446	0.454	0.461	0.467	0.471	0.473	0.476	0.480
136	Bhutan							0.569	0.579	0.580	0.584	0.605
113	Bolivia (Plurinational State of)	0.615	0.636	0.640	0.643	0.649	0.653	0.658	0.661	0.663	0.667	0.662
86	Bosnia and Herzegovina		0.716	0.719	0.722	0.727	0.725	0.726	0.729	0.729	0.731	0.733
109	Botswana	0.560	0.610	0.625	0.643	0.656	0.662	0.672	0.678	0.681	0.683	0.698
79	Brazil	0.682	0.705	0.713	0.721	0.731	0.732	0.739	0.740	0.742	0.744	0.755
30	Brunei Darussalam	0.822	0.838	0.843	0.844	0.843	0.844	0.844	0.846	0.852	0.852	0.856
58	Bulgaria	0.714	0.749	0.753	0.759	0.766	0.767	0.773	0.774	0.776	0.777	0.782
181	Burkina Faso		0.321	0.328	0.338	0.349	0.357	0.367	0.376	0.385	0.388	0.402
180	Burundi	0.290	0.319	0.339	0.350	0.362	0.372	0.381	0.384	0.386	0.389	0.400
123	Cabo Verde	0.573	0.589	0.602	0.607	0.613	0.617	0.622	0.631	0.635	0.636	0.646
136	Cambodia	0.466	0.536	0.547	0.558	0.564	0.566	0.571	0.575	0.579	0.584	0.555
152	Cameroon	0.433	0.457	0.459	0.468	0.477	0.485	0.493	0.498	0.501	0.504	0.512
8	Canada	0.867	0.892	0.894	0.895	0.896	0.894	0.896	0.900	0.901	0.902	0.913
185	Central African Republic	0.314	0.327	0.332	0.338	0.344	0.349	0.355	0.361	0.365	0.341	0.350
184	Chad	0.301	0.324	0.325	0.331	0.338	0.343	0.349	0.365	0.370	0.372	0.392
41	Chile	0.753	0.785	0.784	0.792	0.805	0.804	0.808	0.815	0.819	0.822	0.832
91	China	0.591	0.645	0.657	0.671	0.682	0.693	0.701	0.710	0.715	0.719	0.727
98	Colombia	0.655	0.680	0.685	0.693	0.700	0.703	0.706	0.710	0.708	0.711	0.720

Table B. Human development index (HDI) values

HDI Rank	Countries	2000	2005	2006	2007	2008	2009	2010	2011	2012	2013	2014
159	Comoros		0.464	0.468	0.471	0.474	0.476	0.479	0.483	0.486	0.488	0.503
140	Congo	0.501	0.525	0.535	0.535	0.548	0.559	0.565	0.549	0.561	0.564	0.591
186	Congo (Democratic Republic of the)	0.274	0.292	0.297	0.301	0.307	0.313	0.319	0.323	0.333	0.338	0.433
68	Costa Rica	0.705	0.721	0.729	0.735	0.744	0.746	0.750	0.758	0.761	0.763	0.766
171	Côte d'Ivoire	0.393	0.407	0.414	0.422	0.427	0.433	0.439	0.443	0.448	0.452	0.462
47	Croatia	0.748	0.781	0.788	0.796	0.801	0.800	0.806	0.812	0.812	0.812	0.818
44	Cuba	0.742	0.786	0.806	0.822	0.830	0.832	0.824	0.819	0.813	0.815	0.769
32	Cyprus	0.800	0.828	0.832	0.838	0.844	0.852	0.848	0.850	0.848	0.845	0.850
28	Czech Republic	0.806	0.845	0.848	0.853	0.856	0.856	0.858	0.861	0.861	0.861	0.870
10	Denmark	0.859	0.891	0.893	0.895	0.896	0.895	0.898	0.899	0.900	0.900	0.923
170	Djibouti		0.412	0.417	0.428	0.438	0.444	0.452	0.461	0.465	0.467	0.470
93	Dominica	0.691	0.708	0.708	0.712	0.712	0.717	0.717	0.718	0.716	0.717	0.724
102	Dominican Republic	0.645	0.668	0.675	0.681	0.684	0.686	0.691	0.695	0.698	0.700	0.715
98	Ecuador	0.658	0.687	0.690	0.692	0.697	0.698	0.701	0.705	0.708	0.711	0.732
110	Egypt	0.621	0.645	0.652	0.659	0.667	0.672	0.678	0.679	0.681	0.682	0.690
115	El Salvador	0.607	0.640	0.644	0.646	0.648	0.649	0.652	0.657	0.660	0.662	0.666
144	Equatorial Guinea	0.476	0.517	0.528	0.533	0.543	0.543	0.559	0.553	0.556	0.556	0.587
182	Eritrea							0.373	0.377	0.380	0.381	0.391
33	Estonia	0.776	0.821	0.827	0.832	0.832	0.827	0.830	0.836	0.839	0.840	0.861
173	Ethiopia	0.284	0.339	0.356	0.378	0.394	0.403	0.409	0.422	0.429	0.435	0.442
88	Fiji	0.674	0.694	0.704	0.707	0.712	0.717	0.721	0.722	0.722	0.724	0.727
24	Finland	0.841	0.869	0.874	0.877	0.878	0.873	0.877	0.879	0.879	0.879	0.883
20	France	0.848	0.867	0.870	0.873	0.875	0.876	0.879	0.882	0.884	0.884	0.888
112	Gabon	0.632	0.644	0.645	0.651	0.654	0.659	0.662	0.666	0.670	0.674	0.684
172	Gambia	0.383	0.414	0.418	0.425	0.432	0.436	0.440	0.436	0.438	0.441	0.441
79	Georgia		0.710	0.714	0.726	0.730	0.735	0.733	0.736	0.741	0.744	0.754
6	Germany	0.854	0.887	0.896	0.899	0.902	0.901	0.904	0.908	0.911	0.911	0.916
138	Ghana	0.487	0.511	0.521	0.532	0.544	0.549	0.556	0.566	0.571	0.573	0.579
29	Greece	0.798	0.853	0.859	0.857	0.858	0.858	0.856	0.854	0.854	0.853	0.865
79	Grenada							0.746	0.747	0.743	0.744	0.750
125	Guatemala	0.551	0.576	0.583	0.595	0.601	0.606	0.613	0.620	0.626	0.628	0.627
179	Guinea		0.366	0.374	0.377	0.377	0.376	0.380	0.387	0.391	0.392	0.411
177	Guinea-Bissau		0.387	0.393	0.395	0.397	0.397	0.401	0.402	0.396	0.396	0.420
121	Guyana	0.570	0.584	0.589	0.616	0.621	0.623	0.626	0.632	0.635	0.638	0.636
168	Haiti	0.433	0.447	0.450	0.455	0.458	0.463	0.462	0.466	0.469	0.471	0.483
129	Honduras	0.558	0.584	0.590	0.597	0.604	0.607	0.612	0.615	0.616	0.617	0.606
15	Hong Kong, China (SAR)	0.810	0.839	0.847	0.860	0.877	0.879	0.882	0.886	0.889	0.891	0.910
43	Hungary	0.774	0.805	0.810	0.813	0.814	0.816	0.817	0.817	0.817	0.818	0.828
13	Iceland	0.858	0.888	0.890	0.894	0.886	0.885	0.886	0.890	0.893	0.895	0.899
135	India	0.483	0.527	0.537	0.547	0.554	0.560	0.570	0.581	0.583	0.586	0.609
108	Indonesia	0.609	0.640	0.645	0.650	0.654	0.665	0.671	0.678	0.681	0.684	0.684
75	Iran (Islamic Republic of)	0.652	0.681	0.691	0.703	0.711	0.718	0.725	0.733	0.749	0.749	0.766
120	Iraq	0.606	0.621	0.636	0.632	0.632	0.637	0.638	0.639	0.641	0.642	0.654
11	Ireland	0.862	0.890	0.895	0.901	0.902	0.898	0.899	0.900	0.901	0.899	0.916
19	Israel	0.849	0.869	0.872	0.877	0.877	0.878	0.881	0.885	0.886	0.888	0.894

Table B. Human development index (HDI) values

HDI Rank	Countries	2000	2005	2006	2007	2008	2009	2010	2011	2012	2013	2014
26	Italy	0.825	0.858	0.863	0.867	0.868	0.866	0.869	0.872	0.872	0.872	0.873
96	Jamaica	0.671	0.700	0.702	0.707	0.710	0.709	0.712	0.714	0.715	0.715	0.719
17	Japan	0.858	0.873	0.877	0.879	0.881	0.880	0.884	0.887	0.888	0.890	0.891
77	Jordan	0.705	0.733	0.736	0.741	0.746	0.746	0.744	0.744	0.744	0.745	0.748
70	Kazakhstan	0.679	0.734	0.740	0.743	0.744	0.746	0.747	0.750	0.755	0.757	0.788
147	Kenya	0.455	0.479	0.487	0.500	0.508	0.516	0.522	0.527	0.531	0.535	0.548
133	Kiribati							0.599	0.599	0.606	0.607	0.590
15	Korea (Republic of)	0.819	0.856	0.862	0.869	0.874	0.876	0.882	0.886	0.888	0.891	0.898
46	Kuwait	0.804	0.795	0.795	0.797	0.800	0.804	0.807	0.810	0.813	0.814	0.816
125	Kyrgyzstan	0.586	0.605	0.609	0.614	0.617	0.617	0.614	0.618	0.621	0.628	0.655
139	Lao People's Democratic Republic	0.473	0.511	0.517	0.525	0.533	0.543	0.549	0.560	0.565	0.569	0.575
48	Latvia	0.729	0.786	0.796	0.804	0.813	0.814	0.809	0.804	0.808	0.810	0.819
65	Lebanon		0.741	0.738	0.745	0.750	0.756	0.759	0.764	0.764	0.765	0.769
162	Lesotho	0.443	0.437	0.441	0.448	0.456	0.464	0.472	0.476	0.481	0.486	0.497
175	Liberia	0.339	0.335	0.350	0.364	0.374	0.386	0.393	0.402	0.407	0.412	0.430
55	Libya	0.745	0.772	0.778	0.784	0.789	0.794	0.799	0.753	0.789	0.784	0.724
18	Liechtenstein							0.882	0.887	0.888	0.889	0.908
35	Lithuania	0.757	0.806	0.814	0.820	0.827	0.833	0.829	0.828	0.831	0.834	0.839
21	Luxembourg	0.866	0.876	0.877	0.880	0.882	0.876	0.881	0.881	0.880	0.881	0.892
155	Madagascar	0.453	0.470	0.476	0.480	0.487	0.496	0.494	0.495	0.496	0.498	0.510
174	Malawi	0.341	0.368	0.375	0.383	0.395	0.407	0.406	0.411	0.411	0.414	0.445
62	Malaysia	0.717	0.747	0.752	0.757	0.760	0.761	0.766	0.768	0.770	0.773	0.779
103	Maldives	0.599	0.659	0.661	0.674	0.675	0.686	0.688	0.692	0.695	0.698	0.706
176	Mali	0.309	0.359	0.367	0.377	0.385	0.393	0.398	0.405	0.406	0.407	0.419
39	Malta	0.770	0.801	0.801	0.802	0.809	0.818	0.821	0.823	0.827	0.829	0.839
161	Mauritania	0.433	0.455	0.465	0.467	0.466	0.474	0.475	0.475	0.485	0.487	0.506
63	Mauritius	0.686	0.722	0.728	0.735	0.741	0.747	0.753	0.759	0.769	0.771	0.777
71	Mexico	0.699	0.724	0.732	0.735	0.739	0.741	0.748	0.752	0.755	0.756	0.756
124	Micronesia (Federated States of)							0.627	0.627	0.629	0.630	0.640
114	Moldova (Republic of)	0.598	0.639	0.645	0.646	0.652	0.646	0.652	0.656	0.657	0.663	0.693
103	Mongolia	0.580	0.637	0.646	0.655	0.665	0.668	0.671	0.682	0.692	0.698	0.727
51	Montenegro		0.750	0.760	0.771	0.780	0.780	0.784	0.787	0.787	0.789	0.802
129	Morocco	0.526	0.569	0.575	0.582	0.588	0.594	0.603	0.612	0.614	0.617	0.628
178	Mozambique	0.285	0.343	0.348	0.359	0.366	0.375	0.380	0.384	0.389	0.393	0.416
150	Myanmar	0.421	0.472	0.483	0.493	0.500	0.507	0.514	0.517	0.520	0.524	0.536
127	Namibia	0.556	0.570	0.580	0.589	0.598	0.603	0.610	0.616	0.620	0.624	0.628
145	Nepal	0.449	0.477	0.487	0.492	0.501	0.513	0.527	0.533	0.537	0.540	0.548
4	Netherlands	0.874	0.888	0.895	0.901	0.901	0.900	0.904	0.914	0.915	0.915	0.922
7	New Zealand	0.873	0.894	0.896	0.899	0.899	0.903	0.903	0.904	0.908	0.910	0.913
132	Nicaragua	0.554	0.585	0.590	0.595	0.599	0.600	0.604	0.608	0.611	0.614	0.631
187	Niger	0.262	0.293	0.297	0.303	0.309	0.312	0.323	0.328	0.335	0.337	0.348
152	Nigeria		0.466	0.471	0.480	0.483	0.488	0.492	0.496	0.500	0.504	0.514
1	Norway	0.910	0.935	0.938	0.938	0.937	0.937	0.939	0.941	0.943	0.944	0.944
56	Oman		0.733	0.693	0.701	0.714	0.728	0.780	0.781	0.781	0.783	0.793
146	Pakistan	0.454	0.504	0.527	0.535	0.536	0.545	0.526	0.531	0.535	0.537	0.538

Table B. Human development index (HDI) values

HDI Rank	Countries	2000	2005	2006	2007	2008	2009	2010	2011	2012	2013	2014
60	Palau	0.741	0.771	0.773	0.773	0.772	0.773	0.768	0.770	0.773	0.775	0.780
107	Palestine, State of		0.649	0.658	0.668	0.672	0.679	0.671	0.679	0.683	0.686	0.677
65	Panama	0.709	0.728	0.742	0.747	0.752	0.754	0.759	0.757	0.761	0.765	0.780
157	Papua New Guinea	0.423	0.441	0.452	0.454	0.467	0.474	0.479	0.484	0.490	0.491	0.505
111	Paraguay	0.625	0.648	0.648	0.653	0.661	0.661	0.669	0.672	0.670	0.676	0.679
82	Peru	0.682	0.694	0.696	0.700	0.707	0.709	0.722	0.727	0.734	0.737	0.734
117	Philippines	0.619	0.638	0.638	0.644	0.648	0.647	0.651	0.652	0.656	0.660	0.668
35	Poland	0.784	0.803	0.808	0.812	0.817	0.820	0.826	0.830	0.833	0.834	0.843
41	Portugal	0.780	0.790	0.794	0.800	0.805	0.809	0.816	0.819	0.822	0.822	0.830
31	Qatar	0.811	0.840	0.846	0.852	0.855	0.850	0.847	0.843	0.850	0.851	0.850
54	Romania	0.706	0.750	0.759	0.769	0.781	0.781	0.779	0.782	0.782	0.785	0.793
57	Russian Federation	0.717	0.750	0.757	0.765	0.770	0.770	0.773	0.775	0.777	0.778	0.798
151	Rwanda	0.329	0.391	0.414	0.423	0.432	0.443	0.453	0.463	0.502	0.506	0.483
73	Saint Kitts and Nevis						0.752	0.747	0.745	0.749	0.750	0.752
97	Saint Lucia						0.720	0.717	0.718	0.715	0.714	0.729
91	Saint Vincent and the Grenadines						0.717	0.717	0.715	0.717	0.719	0.720
106	Samoa	0.654	0.681	0.681	0.684	0.683	0.689	0.688	0.690	0.693	0.694	0.702
142	Sao Tome and Principe	0.495	0.520	0.520	0.531	0.537	0.543	0.543	0.548	0.556	0.558	0.555
34	Saudi Arabia	0.744	0.773	0.779	0.784	0.791	0.802	0.815	0.825	0.833	0.836	0.837
163	Senegal	0.413	0.451	0.456	0.466	0.474	0.478	0.483	0.483	0.484	0.485	0.466
77	Serbia	0.713	0.732	0.735	0.739	0.743	0.742	0.743	0.744	0.743	0.745	0.771
71	Seychelles	0.743	0.757	0.760	0.763	0.766	0.744	0.763	0.749	0.755	0.756	0.772
183	Sierra Leone	0.297	0.329	0.335	0.340	0.346	0.350	0.353	0.360	0.368	0.374	0.413
9	Singapore	0.800	0.840	0.852	0.862	0.868	0.868	0.894	0.896	0.899	0.901	0.912
37	Slovakia	0.776	0.803	0.810	0.817	0.824	0.826	0.826	0.827	0.829	0.830	0.844
25	Slovenia	0.821	0.855	0.861	0.865	0.871	0.875	0.873	0.874	0.874	0.874	0.880
157	Solomon Islands	0.475	0.483	0.493	0.498	0.506	0.500	0.489	0.494	0.489	0.491	0.506
118	South Africa	0.628	0.608	0.611	0.617	0.623	0.631	0.638	0.646	0.654	0.658	0.666
	South Sudan											0.467
27	Spain	0.826	0.844	0.848	0.852	0.857	0.858	0.864	0.868	0.869	0.869	0.876
73	Sri Lanka	0.679	0.710	0.716	0.721	0.725	0.728	0.736	0.740	0.745	0.750	0.757
166	Sudan	0.385	0.423	0.430	0.440	0.447	0.458	0.463	0.468	0.472	0.473	0.479
100	Suriname		0.672	0.676	0.690	0.694	0.696	0.698	0.701	0.702	0.705	0.714
148	Swaziland	0.498	0.498	0.505	0.513	0.518	0.523	0.527	0.530	0.529	0.530	0.531
12	Sweden	0.889	0.887	0.889	0.891	0.891	0.888	0.895	0.896	0.897	0.898	0.907
3	Switzerland	0.886	0.901	0.905	0.905	0.903	0.909	0.915	0.914	0.916	0.917	0.930
118	Syrian Arab Republic	0.605	0.653		0.658	0.658	0.662	0.662	0.662	0.662	0.658	0.594
133	Tajikistan	0.529	0.572	0.578	0.583	0.591	0.592	0.596	0.600	0.603	0.607	0.624
159	Tanzania (United Republic of)	0.376	0.419	0.430	0.440	0.451	0.457	0.464	0.478	0.484	0.488	0.521
89	Thailand	0.649	0.685	0.685	0.698	0.704	0.708	0.715	0.716	0.720	0.722	0.726
84	The former Yugoslav Republic of Macedonia		0.699	0.705	0.708	0.724	0.725	0.728	0.730	0.730	0.732	0.747
128	Timor-Leste	0.465	0.505	0.521	0.552	0.579	0.599	0.606	0.606	0.616	0.620	0.595
166	Togo	0.430	0.442	0.448	0.446	0.447	0.454	0.460	0.467	0.470	0.473	0.484
100	Tonga	0.672	0.695	0.696	0.697	0.696	0.698	0.701	0.702	0.704	0.705	0.717
64	Trinidad and Tobago	0.697	0.745	0.751	0.759	0.764	0.766	0.764	0.764	0.765	0.766	0.772

Table B. Human development index (HDI) values

HDI Rank	Countries	2000	2005	2006	2007	2008	2009	2010	2011	2012	2013	2014
90	Tunisia	0.653	0.687	0.694	0.700	0.706	0.710	0.715	0.716	0.719	0.721	0.721
69	Turkey	0.653	0.687	0.698	0.706	0.710	0.716	0.738	0.752	0.756	0.759	0.761
103	Turkmenistan							0.687	0.690	0.693	0.698	0.688
164	Uganda	0.392	0.429	0.437	0.446	0.458	0.466	0.472	0.477	0.480	0.484	0.483
83	Ukraine	0.668	0.713	0.720	0.726	0.729	0.722	0.726	0.730	0.733	0.734	0.747
40	United Arab Emirates	0.797	0.823	0.826	0.829	0.832	0.826	0.824	0.824	0.825	0.827	0.835
14	United Kingdom	0.863	0.888	0.885	0.887	0.890	0.890	0.895	0.891	0.890	0.892	0.907
5	United States	0.883	0.897	0.900	0.903	0.905	0.905	0.908	0.911	0.912	0.914	0.915
50	Uruguay	0.740	0.755	0.759	0.769	0.773	0.774	0.779	0.783	0.787	0.790	0.793
116	Uzbekistan		0.626	0.630	0.635	0.643	0.645	0.648	0.653	0.657	0.661	0.675
131	Vanuatu					0.608	0.616	0.617	0.618	0.617	0.616	0.594
67	Venezuela (Bolivarian Republic of)	0.677	0.716	0.731	0.748	0.758	0.757	0.759	0.761	0.763	0.764	0.762
121	Viet Nam	0.563	0.598	0.604	0.611	0.617	0.622	0.629	0.632	0.635	0.638	0.666
154	Yemen	0.427	0.462	0.465	0.468	0.471	0.478	0.484	0.497	0.499	0.500	0.498
141	Zambia	0.423	0.471	0.480	0.486	0.505	0.526	0.530	0.543	0.554	0.561	0.586
156	Zimbabwe	0.428	0.412	0.417	0.424	0.422	0.439	0.459	0.473	0.484	0.492	0.509

Table C. HDI rank for each country per year

HDI Rank	Countries	2000	2005	2006	2007	2008	2009	2010	2011	2012	2013	2014
169	Afghanistan	146	159	159	160	159	162	169	170	169	169	171
95	Albania	77	84	85	87	88	91	96	96	97	95	85
93	Algeria	87	93	93	93	92	93	95	94	94	94	83
37	Andorra							33	34	37	37	34
149	Angola	144	147	143	141	140	144	150	149	149	149	149
61	Antigua and Barbuda							58	59	60	61	58
49	Argentina	44	51	50	50	51	48	50	49	49	49	40
87	Armenia	84	83	77	75	77	81	87	86	87	87	85
2	Australia	2	2	2	2	2	2	2	2	2	2	2
21	Austria	20	24	24	24	25	23	24	24	23	23	23
76	Azerbaijan	86	88	80	76	75	71	76	77	75	76	78
51	Bahamas	41	42	44	46	46	49	51	50	51	51	55
44	Bahrain	35	34	37	39	40	41	44	45	45	45	45
142	Bangladesh	125	133	132	133	134	136	143	142	143	143	142
59	Barbados	47	50	51	51	52	50	54	56	58	59	57
53	Belarus		66	63	60	59	53	56	52	54	53	50
21	Belgium	8	19	19	19	21	22	23	22	22	22	21
84	Belize	71	74	75	79	82	86	93	90	84	84	101
165	Benin	141	151	150	151	152	155	162	164	165	165	166
136	Bhutan							137	136	136	136	132
113	Bolivia (Plurinational State of)	93	108	106	108	107	109	114	114	113	113	119
86	Bosnia and Herzegovina		71	71	72	73	76	82	84	86	86	85
109	Botswana	106	111	110	109	103	106	107	109	109	109	106
79	Brazil	66	77	74	74	70	73	77	78	80	80	75
30	Brunei Darussalam	23	30	30	30	31	31	32	31	30	30	31

Table C. HDI rank for each country per year

HDI Rank	Countries	2000	2005	2006	2007	2008	2009	2010	2011	2012	2013	2014
58	Bulgaria	56	57	57	57	57	57	60	58	59	58	59
181	Burkina Faso		171	170	171	170	173	182	182	181	181	183
180	Burundi	154	172	167	168	169	172	178	179	180	180	184
123	Cabo Verde	103	115	114	116	116	119	124	123	123	123	122
136	Cambodia	121	123	122	123	125	128	135	137	137	137	143
152	Cameroon	129	143	144	144	143	146	152	151	152	152	153
8	Canada	9	6	9	9	9	10	10	9	9	8	9
185	Central African Republic	150	169	169	170	172	175	183	184	185	185	187
184	Chad	152	170	171	172	173	176	185	183	183	184	185
41	Chile	43	45	46	45	43	43	46	44	42	42	42
91	China	100	101	98	97	96	97	100	98	96	92	90
98	Colombia	78	92	91	90	89	92	97	97	98	98	97
159	Comoros		141	140	142	144	149	157	158	158	159	159
140	Congo	113	125	124	126	127	130	138	141	140	140	136
186	Congo (Democratic Republic of the)	157	174	173	174	175	177	187	187	187	186	176
68	Costa Rica	60	69	68	68	66	67	69	66	67	68	69
171	Côte d'Ivoire	139	158	157	159	160	164	172	171	171	171	172
47	Croatia	45	46	45	44	44	46	48	46	47	47	47
44	Cuba	50	43	39	34	34	33	38	41	44	44	67
32	Cyprus	31	31	31	31	30	29	30	30	32	32	32
28	Czech Republic	28	25	26	27	28	28	28	28	28	28	28
10	Denmark	13	7	10	10	10	9	9	10	10	10	4
170	Djibouti		157	156	155	156	159	170	169	170	170	168
93	Dominica	64	76	76	77	79	82	89	89	92	93	94
102	Dominican Republic	85	95	95	95	94	99	102	102	102	102	101
98	Ecuador	76	85	89	91	90	94	98	99	99	99	88
110	Egypt	91	100	99	99	99	102	106	107	108	110	108
115	El Salvador	95	104	105	106	108	110	115	115	115	115	116
144	Equatorial Guinea	118	127	125	128	129	133	139	140	141	144	138
182	Eritrea							181	181	182	182	186
33	Estonia	37	33	32	32	32	34	34	33	33	33	30
173	Ethiopia	156	166	164	163	164	166	173	173	173	173	174
88	Fiji	72	82	79	80	80	83	86	87	88	88	90
24	Finland	19	17	16	16	16	21	22	23	24	24	24
20	France	18	18	18	18	19	18	21	20	20	20	22
112	Gabon	88	102	102	103	104	108	113	112	112	112	110
172	Gambia	143	155	154	156	157	163	171	172	172	172	175
79	Georgia		75	73	71	71	72	80	80	81	81	76
6	Germany	16	13	6	8	6	6	5	6	6	6	6
138	Ghana	116	128	127	129	128	131	140	138	138	138	140
29	Greece	32	23	23	26	26	26	29	29	29	29	29
79	Grenada							73	73	78	79	79
125	Guatemala	110	119	118	119	119	122	127	125	125	125	128
179	Guinea		163	162	164	166	170	179	178	178	179	182
177	Guinea-Bissau		161	160	161	162	167	175	176	177	177	178

Table C. HDI rank for each country per year

HDI Rank	Countries	2000	2005	2006	2007	2008	2009	2010	2011	2012	2013	2014
121	Guyana	104	117	117	113	113	116	123	122	122	122	124
168	Haiti	131	146	147	147	149	154	165	167	168	168	163
129	Honduras	107	118	116	117	118	121	128	129	129	129	131
15	Hong Kong, China (SAR)	27	29	28	25	18	15	16	17	15	15	12
43	Hungary	39	36	35	37	38	39	41	43	43	43	44
13	Iceland	14	11	11	11	13	13	14	14	13	13	16
135	India	117	124	123	125	126	129	136	135	135	135	130
108	Indonesia	94	103	103	104	105	104	110	110	110	108	110
75	Iran (Islamic Republic of)	82	91	88	83	81	80	84	81	74	75	69
120	Iraq	96	110	108	111	111	114	119	120	120	120	121
11	Ireland	12	8	7	5	5	8	8	8	8	11	6
19	Israel	17	16	17	17	17	16	19	19	19	19	18
26	Italy	22	20	20	21	23	25	26	26	26	26	27
96	Jamaica	74	78	81	81	83	88	94	95	95	96	99
17	Japan	15	15	15	15	15	14	15	15	16	17	20
77	Jordan	61	62	64	65	64	65	74	75	77	77	80
70	Kazakhstan	68	61	61	64	65	66	72	71	71	70	56
147	Kenya	123	135	134	134	135	139	148	148	147	147	145
133	Kiribati							133	134	133	133	137
15	Korea (Republic of)	25	21	21	20	20	19	17	18	18	16	17
46	Kuwait	29	40	42	43	45	44	47	47	46	46	48
125	Kyrgyzstan	101	113	112	114	114	118	126	127	126	126	120
139	Lao People's Democratic Republic	120	129	130	131	132	135	141	139	139	139	141
48	Latvia	53	44	41	40	39	40	45	48	48	48	46
65	Lebanon		60	62	63	63	61	66	63	65	65	67
162	Lesotho	128	150	149	149	151	153	161	161	163	162	161
175	Liberia	148	167	165	166	167	169	177	177	175	175	177
55	Libya	46	48	48	48	48	47	49	68	50	55	94
18	Liechtenstein							18	16	17	18	13
35	Lithuania	42	35	34	35	35	32	35	36	36	36	37
21	Luxembourg	10	14	14	14	14	17	20	21	21	21	19
155	Madagascar	126	139	138	139	141	143	151	154	155	155	154
174	Malawi	147	162	161	162	163	165	174	174	174	174	173
62	Malaysia	55	58	58	59	60	59	62	61	62	62	62
103	Maldives	98	96	96	96	97	100	104	103	103	103	104
176	Mali	151	164	163	165	165	168	176	175	176	176	179
39	Malta	40	39	40	41	41	38	40	40	39	39	37
161	Mauritania	130	144	142	145	148	151	159	162	159	161	156
63	Mauritius	65	68	69	69	68	64	68	65	63	63	63
71	Mexico	62	67	66	67	69	70	70	69	70	71	74
124	Micronesia (Federated States of)							122	124	124	124	123
114	Moldova (Republic of)	99	105	104	105	106	112	116	116	116	114	107
103	Mongolia	102	107	101	101	100	103	109	106	106	105	90
51	Montenegro		56	53	52	50	52	52	51	52	52	49
129	Morocco	112	122	121	122	123	126	132	130	131	130	126

Table C. HDI rank for each country per year

HDI Rank	Countries	2000	2005	2006	2007	2008	2009	2010	2011	2012	2013	2014
178	Mozambique	155	165	166	167	168	171	180	180	179	178	180
150	Myanmar	137	137	136	136	139	141	149	150	150	150	148
127	Namibia	108	121	119	120	121	123	129	128	127	127	126
145	Nepal	127	136	135	137	138	140	146	145	145	145	145
4	Netherlands	6	9	8	6	7	7	6	4	4	4	5
7	New Zealand	7	5	5	7	8	5	7	7	7	7	9
132	Nicaragua	109	116	115	118	120	124	131	131	132	132	125
187	Niger	158	173	172	173	174	178	186	186	186	187	188
152	Nigeria		140	139	140	142	145	153	153	153	153	152
1	Norway	1	1	1	1	1	1	1	1	1	1	1
56	Oman		63	87	84	78	75	53	55	56	56	52
146	Pakistan	124	131	126	127	131	132	147	146	146	146	147
60	Palau	51	49	49	49	54	55	61	60	61	60	60
107	Palestine, State of		98	97	98	98	101	108	108	107	107	113
65	Panama	58	65	60	62	62	62	67	67	68	66	60
157	Papua New Guinea	136	149	146	148	147	150	158	156	156	157	158
111	Paraguay	90	99	100	102	101	107	111	111	111	111	112
82	Peru	67	81	84	85	85	89	85	85	82	82	84
117	Philippines	92	106	107	107	109	111	117	118	118	117	115
35	Poland	34	37	38	38	37	37	37	35	34	35	36
41	Portugal	36	41	43	42	42	42	42	42	41	41	43
31	Qatar	26	27	29	29	29	30	31	32	31	31	32
54	Romania	59	55	55	54	49	51	55	54	55	54	52
57	Russian Federation	54	54	56	55	55	56	59	57	57	57	50
151	Rwanda	149	160	158	158	158	160	168	168	151	151	163
73	Saint Kitts and Nevis						63	71	74	73	73	77
97	Saint Lucia						79	88	88	93	97	89
91	Saint Vincent and the Grenadines						84	90	93	91	91	97
106	Samoa	79	90	92	94	95	98	103	104	104	106	105
142	Sao Tome and Principe	115	126	129	130	130	134	142	143	142	142	143
34	Saudi Arabia	48	47	47	47	47	45	43	38	35	34	39
163	Senegal	138	145	145	146	145	147	156	157	160	163	170
77	Serbia	57	64	65	66	67	69	75	76	79	78	66
71	Seychelles	49	52	52	56	56	68	64	72	72	72	64
183	Sierra Leone	153	168	168	169	171	174	184	185	184	183	181
9	Singapore	30	28	25	23	24	24	13	12	11	9	11
37	Slovakia	38	38	36	36	36	36	36	37	38	38	35
25	Slovenia	24	22	22	22	22	20	25	25	25	25	25
157	Solomon Islands	119	134	133	135	136	142	154	155	157	158	156
118	South Africa	89	112	111	112	112	115	120	119	119	119	116
188	South Sudan											169
27	Spain	21	26	27	28	27	27	27	27	27	27	26
73	Sri Lanka	69	73	72	73	74	74	79	79	76	74	73
166	Sudan	142	153	152	153	155	156	164	165	166	166	167
100	Suriname		94	94	92	93	96	101	101	101	101	103

Table C. HDI rank for each country per year

HDI Rank	Countries	2000	2005	2006	2007	2008	2009	2010	2011	2012	2013	2014
148	Swaziland	114	132	131	132	133	138	145	147	148	148	150
12	Sweden	3	12	12	12	11	12	12	11	12	12	14
3	Switzerland	4	3	3	3	4	3	3	3	3	3	3
118	Syrian Arab Republic	97	97		100	102	105	112	113	114	118	134
133	Tajikistan	111	120	120	121	122	127	134	133	134	134	129
159	Tanzania (United Republic of)	145	154	153	154	153	157	163	159	161	160	151
89	Thailand	83	89	90	88	87	90	92	92	89	89	93
84	The former Yugoslav Republic of Macedonia		79	78	78	76	77	81	82	85	85	81
128	Timor-Leste	122	130	128	124	124	125	130	132	130	128	133
166	Togo	132	148	148	150	154	158	166	166	167	167	162
100	Tonga	73	80	83	89	91	95	99	100	100	100	100
64	Trinidad and Tobago	63	59	59	58	58	58	63	62	64	64	64
90	Tunisia	81	87	86	86	86	87	91	91	90	90	96
69	Turkey	80	86	82	82	84	85	78	70	69	69	72
103	Turkmenistan							105	105	105	104	109
164	Uganda	140	152	151	152	150	152	160	160	164	164	163
83	Ukraine	75	72	70	70	72	78	83	83	83	83	81
40	United Arab Emirates	33	32	33	33	33	35	39	39	40	40	41
14	United Kingdom	11	10	13	13	12	11	11	13	14	14	14
5	United States	5	4	4	4	3	4	4	5	5	5	8
50	Uruguay	52	53	54	53	53	54	57	53	53	50	52
116	Uzbekistan		109	109	110	110	113	118	117	117	116	114
131	Vanuatu					117	120	125	126	128	131	134
67	Venezuela (Bolivarian Republic of)	70	70	67	61	61	60	65	64	66	67	71
121	Viet Nam	105	114	113	115	115	117	121	121	121	121	116
154	Yemen	134	142	141	143	146	148	155	152	154	154	160
141	Zambia	135	138	137	138	137	137	144	144	144	141	139
156	Zimbabwe	133	156	155	157	161	161	167	163	162	156	155
	**Normalized value**	0.203	0.201	0.202	0.201	0.194	0.197	0.193	0.176	0.171	0.171	0.181
	**Total...**	**158**	**174**	**173**	**174**	**175**	**178**	**187**	**187**	**187**	**187**	**188**

International Human Development Indicators
Accessed: 7/14/2014,2:06 PM from: http://hdr.undp.org
Percentage of the population living below the international poverty line $1.25 (in purchasing power parity terms) a day.
Source: World Bank (2013). "World Development Indicators 2013." Washington, D.C.: World Bank. http://data.worldbank.org. Accessed October, 2013. http://data.worldbank.org/data-catalog/world-development-indicators
Data in the tables are those available to the Human Development Report Office as of 15 November, 2013, unless otherwise specified.

Table D. Percentage of the population living below the international poverty line $1.25 (in purchasing power parity terms) a day

HDI Rank	Country	1980	1985	1990	2000	2005	2006	2007	2008	2009	2010	2011
33	Estonia				0.6							
35	Poland		0.1		0.1	0.1	0.1	0.1	0.1	0.1	0.1	0.1
35	Lithuania				0.3				0.2			
37	Slovakia					0.2	0.1	0.1	0.1	0.1		
41	Chile			5.1	2.3		1.1			1.4		
43	Hungary				0.2			0.2				
47	Croatia				0.1				0.1			
48	Latvia							0.2	0.1	0.2		
49	Argentina				5.1	4.6	3.7	2.7	1.9	2	0.9	
50	Uruguay				0.5	1.4	0.7	0.5	0.3	0.3	0.2	
51	Montenegro					0.1	0.1	0.2	0.1	0	0	
53	Belarus				0.3	0.2	0.1	0.1	0.1	0.2	0.1	0.1
54	Romania				3.7	1.2	1	0.8	0.5	0.4	0.5	0.4
57	Russian Federation					0.2	0.1	0	0	0		
58	Bulgaria							0				
62	Malaysia							0		0		
65	Panama					9.5	10.2			5.9	6.6	
67	Venezuela (Bolivarian Republic of)					13.4	6.6					
68	Costa Rica			8.5	5.5	3.9	4	2.2	2.4	3.1		
69	Turkey					2	1.5	1.1	0	0.6	1.3	
70	Kazakhstan						0.4	0.2	0.1	0.1		
71	Mexico				5.5	5.1	0.7		1.2		0.7	
71	Seychelles				0			0.3				
73	Sri Lanka		20					7			4.1	
75	Iran (Islamic Republic of)			3.9		1.5						
76	Azerbaijan								0.4			
77	Serbia					0	0.3	0.2	0.1	0.3	0.2	
77	Jordan						0.4		0.1		0.1	
79	Georgia				19.3	16	15.6	15.2	15.3	15.2	18	
79	Brazil		13.5	17.2		8.5	7.6	7.1	6	6.1		
82	Peru				12.4	8.6	7.2	7.9	6.2	5.5	4.9	
83	Ukraine					0.1	0.1	0.1	0	0.1	0	

Table D. Percentage of the population living below the international poverty line $1.25 (in purchasing power parity terms) a day

HDI Rank	Country	1980	1985	1990	2000	2005	2006	2007	2008	2009	2010	2011
84	The former Yugoslav Republic of Macedonia				4.3	0.3	0.9		0.3	0	0.6	
86	Bosnia and Herzegovina							0				
87	Armenia					4	3	3.5	1.3		2.5	
88	Fiji									5.9		
89	Thailand			11.6	3		1		0.4	0.4	0.4	
90	Tunisia		8.7	5.9	2.6	1.4					1.1	
91	China			60.2		16.3			13.1	11.8		
95	Albania					0.9			0.6			
96	Jamaica			1.3								
98	Colombia	15.5			17.9	12.7	11	8.8	11.3	9.7	8.2	
98	Ecuador				20.7	9.1	6.1	7.2	6.5	6.4	4.6	
102	Dominican Republic				5.2	6.1	4.2	3.8	3.3	3	2.2	
107	Palestine, State of							0.4		0		
108	Indonesia			54.3		21.4	28.6	24.2	22.6	20.4	18.1	16.2
110	Egypt				1.8	2			1.7			
111	Paraguay			1		7.2	10.9	8.8	5.6	7.6	7.2	
112	Gabon					4.8						
113	Bolivia (Plurinational State of)				26.9	18.2	16.2	13.1	15.6			
114	Moldova (Republic of)					12.5	2	0.9	1.1	0.4	0.4	
115	El Salvador					11.6	5.3	6.6	5.4	9		
117	Philippines		34.9		22.5		22.6			18.4		
118	South Africa				26.2		17.4			13.8		
120	Iraq							2.8				
121	Viet Nam						21.4		16.9			
124	Micronesia (Federated States of)				31.2							
125	Kyrgyzstan					22.9	5.9	1.9	6.4	6.2	6.7	5
125	Guatemala				11.9		13.5					
129	Honduras			46.9		26.4	22.9	16.3	21.4	17.9		
129	Morocco		10.3					2.5				
132	Nicaragua					11.9						
133	Tajikistan							14.7		6.6		
135	India					41.6					32.7	
136	Bhutan							10.2				
136	Cambodia							32.2	22.8	18.6		
138	Ghana						28.6					
139	Lao People's Democratic Republic								33.9			
140	Congo					54.1						
141	Zambia						68.5				74.5	

Table D. Percentage of the population living below the international poverty line $1.25 (in purchasing power parity terms) a day

HDI Rank	Country	1980	1985	1990	2000	2005	2006	2007	2008	2009	2010	2011
142	Bangladesh				58.6	50.5					43.3	
145	Nepal		78.2								24.8	
146	Pakistan					22.6	22.6		21			
147	Kenya					43.4						
148	Swaziland										40.6	
149	Angola				54.3					43.4		
151	Rwanda		63.3		74.6		72.1					63.2
152	Nigeria										68	
152	Cameroon							9.6				
154	Yemen					17.5						
155	Madagascar	85.9				67.8					81.3	
159	Tanzania (United Republic of)				84.6			67.9				
161	Mauritania				21.2				23.4			
163	Senegal					33.5						29.6
164	Uganda						51.5			38		
166	Sudan									19.8		
166	Togo						38.7					28.2
171	Côte d'Ivoire		9.5						23.8			
173	Ethiopia				55.6	39						30.7
174	Malawi										61.6	
175	Liberia							83.8				
176	Mali						51.4				50.4	
178	Mozambique								59.6			
179	Guinea							43.3				
180	Burundi						81.3					
181	Burkina Faso									44.6		
183	Sierra Leone			62.8								51.7
185	Central African Republic								62.8			
186	Congo (Democratic Republic of the)						87.7					
187	Niger					65.9			43.6			

Table E. Country rank for population living below $1.25 PPP per day (%)

HDI Rank	Country	1980	2005	2010
33	Estonia			
35	Poland		46	31
35	Lithuania			
37	Slovakia		43	

Table E. Country rank for population living below $1.25 PPP per day (%)

HDI Rank	Country	1980	2005	2010
41	Chile			
43	Hungary			
47	Croatia			
48	Latvia			
49	Argentina		30	23
50	Uruguay		37	29
51	Montenegro		44	34
53	Belarus		41	31
54	Romania		38	26
57	Russian Federation		42	
58	Bulgaria			
62	Malaysia			
65	Panama		22	15
67	Venezuela (Bolivarian Republic of)		17	
68	Costa Rica		32	
69	Turkey		34	21
70	Kazakhstan			
71	Mexico		28	24
71	Seychelles			
73	Sri Lanka			18
75	Iran (Islamic Republic of)		35	
76	Azerbaijan			
77	Serbia		47	29
77	Jordan			31
79	Georgia		16	11
79	Brazil		25	
82	Peru		24	16
83	Ukraine		45	34
84	The former Yugoslav Republic of Macedonia		40	25
86	Bosnia and Herzegovina			
87	Armenia		31	19
88	Fiji			
89	Thailand			27
90	Tunisia		36	22
91	China		15	
95	Albania		39	
96	Jamaica			
98	Colombia	2	18	12
98	Ecuador		23	17
102	Dominican Republic		27	20
107	Palestine, State of			

Table E. Country rank for population living below $1.25 PPP per day (%)

HDI Rank	Country	1980	2005	2010
108	Indonesia		12	10
110	Egypt		33	
111	Paraguay		26	13
112	Gabon		29	
113	Bolivia (Plurinational State of)		13	
114	Moldova (Republic of)		19	27
115	El Salvador		21	
117	Philippines			
118	South Africa			
120	Iraq			
121	Viet Nam			
124	Micronesia (Federated States of)			
125	Kyrgyzstan		10	14
125	Guatemala			
129	Honduras		9	
129	Morocco			
132	Nicaragua		20	
133	Tajikistan			
135	India		6	8
136	Bhutan			
136	Cambodia			
138	Ghana			
139	Lao People's Democratic Republic			
140	Congo		3	
141	Zambia			2
142	Bangladesh		4	6
145	Nepal			9
146	Pakistan		11	
147	Kenya		5	
148	Swaziland			7
149	Angola			
151	Rwanda			
152	Nigeria			3
152	Cameroon			
154	Yemen		14	
155	Madagascar	1	1	1
159	Tanzania (United Republic of)			
161	Mauritania			
163	Senegal		8	
164	Uganda			
166	Sudan			

Table E. Country rank for population living below $1.25 PPP per day (%)

HDI Rank	Country	1980	2005	2010
166	Togo			
171	Côte d'Ivoire			
173	Ethiopia		7	
174	Malawi			4
175	Liberia			
176	Mali			5
178	Mozambique			
179	Guinea			
180	Burundi			
181	Burkina Faso			
183	Sierra Leone			
185	Central African Republic			
186	Congo (Democratic Republic of the)			
187	Niger		2	
	**Total..**	2	47	35
	**Normalized value**	0	0	0

International Human Development Indicators
Education index
Source: Calculated using Mean Years of Schooling and Expected Years of Schooling.
Data in the tables are those available to the Human Development Report Office as of 15 November, 2013, unless otherwise specified.

Table F. Mean years of schooling and expected years of schooling

HDI Rank	Countries	2000	2005	2006	2007	2008	2009	2010	2011	2012	2013
1	Norway	0.870	0.910	0.912	0.909	0.903	0.902	0.907	0.910	0.910	0.910
2	Australia	0.895	0.902	0.905	0.907	0.913	0.916	0.919	0.922	0.927	0.927
3	Switzerland	0.804	0.822	0.827	0.828	0.830	0.834	0.841	0.844	0.844	0.844
4	Netherlands	0.824	0.844	0.850	0.860	0.860	0.859	0.866	0.892	0.894	0.894
5	United States	0.850	0.867	0.870	0.876	0.880	0.884	0.887	0.890	0.890	0.890
6	Germany	0.787	0.858	0.872	0.875	0.877	0.879	0.879	0.884	0.884	0.884
7	New Zealand	0.885	0.908	0.910	0.911	0.913	0.915	0.917	0.917	0.917	0.917
8	Canada	0.808	0.853	0.853	0.852	0.851	0.851	0.850	0.850	0.850	0.850
9	Singapore	0.606	0.667	0.684	0.698	0.714	0.715	0.759	0.759	0.759	0.768
10	Denmark	0.804	0.864	0.863	0.865	0.867	0.868	0.873	0.873	0.873	0.873
11	Ireland	0.837	0.866	0.867	0.880	0.884	0.886	0.887	0.887	0.887	0.887
12	Sweden	0.866	0.830	0.827	0.824	0.824	0.823	0.833	0.830	0.830	0.830
13	Iceland	0.786	0.829	0.833	0.836	0.840	0.843	0.847	0.847	0.847	0.847
14	United Kingdom	0.836	0.866	0.854	0.852	0.859	0.865	0.878	0.860	0.860	0.860
15	Hong Kong, China (SAR)	0.648	0.683	0.690	0.713	0.748	0.758	0.759	0.762	0.767	0.767
15	Korea (Republic of)	0.797	0.837	0.843	0.848	0.854	0.856	0.862	0.865	0.865	0.865
17	Japan	0.767	0.785	0.790	0.792	0.795	0.800	0.802	0.808	0.808	0.808
18	Liechtenstein							0.750	0.762	0.762	0.762
19	Israel	0.820	0.848	0.846	0.849	0.844	0.847	0.848	0.851	0.854	0.854
20	France	0.761	0.790	0.792	0.796	0.800	0.803	0.807	0.813	0.816	0.816
21	Luxembourg	0.734	0.743	0.744	0.746	0.748	0.755	0.762	0.762	0.762	0.762
21	Austria	0.730	0.747	0.756	0.759	0.771	0.780	0.794	0.794	0.794	0.794
21	Belgium	0.835	0.792	0.794	0.796	0.797	0.802	0.807	0.812	0.812	0.812
24	Finland	0.765	0.812	0.816	0.814	0.815	0.808	0.812	0.815	0.815	0.815
25	Slovenia	0.794	0.842	0.849	0.850	0.854	0.858	0.864	0.862	0.863	0.863
26	Italy	0.695	0.762	0.768	0.775	0.779	0.780	0.784	0.790	0.790	0.790
27	Spain	0.721	0.742	0.747	0.753	0.763	0.768	0.783	0.792	0.794	0.794
28	Czech Republic	0.783	0.850	0.848	0.854	0.851	0.860	0.863	0.866	0.866	0.866
29	Greece	0.680	0.794	0.803	0.792	0.794	0.795	0.797	0.797	0.797	0.797
30	Brunei Darussalam	0.646	0.672	0.682	0.681	0.677	0.682	0.678	0.681	0.692	0.692
31	Qatar	0.612	0.673	0.686	0.699	0.703	0.689	0.680	0.671	0.686	0.686
32	Cyprus	0.676	0.731	0.740	0.751	0.765	0.778	0.765	0.771	0.776	0.776
33	Estonia	0.808	0.855	0.853	0.854	0.852	0.852	0.859	0.859	0.859	0.859
34	Saudi Arabia	0.565	0.617	0.624	0.630	0.641	0.660	0.688	0.707	0.721	0.723
35	Poland	0.780	0.795	0.797	0.799	0.801	0.808	0.817	0.823	0.825	0.825
35	Lithuania	0.767	0.850	0.856	0.864	0.872	0.882	0.883	0.877	0.877	0.877

Table F. Mean years of schooling and expected years of schooling

HDI Rank	Countries	2000	2005	2006	2007	2008	2009	2010	2011	2012	2013
37	Andorra							0.670	0.670	0.670	0.670
37	Slovakia	0.743	0.784	0.789	0.797	0.800	0.802	0.805	0.802	0.802	0.802
39	Malta	0.616	0.684	0.682	0.679	0.688	0.705	0.722	0.722	0.733	0.733
40	United Arab Emirates	0.604	0.649	0.654	0.659	0.664	0.669	0.673	0.673	0.673	0.673
41	Portugal	0.656	0.661	0.667	0.677	0.690	0.701	0.713	0.720	0.728	0.728
41	Chile	0.650	0.707	0.705	0.717	0.740	0.737	0.740	0.746	0.746	0.746
43	Hungary	0.755	0.793	0.797	0.798	0.799	0.801	0.808	0.805	0.805	0.805
44	Bahrain	0.656	0.711	0.703	0.706	0.708	0.711	0.714	0.714	0.714	0.714
44	Cuba	0.660	0.735	0.771	0.803	0.819	0.819	0.790	0.768	0.743	0.743
46	Kuwait	0.637	0.609	0.610	0.612	0.619	0.626	0.633	0.639	0.646	0.646
47	Croatia	0.656	0.708	0.719	0.729	0.737	0.744	0.760	0.770	0.770	0.770
48	Latvia	0.709	0.796	0.804	0.814	0.824	0.823	0.822	0.813	0.813	0.813
49	Argentina	0.711	0.726	0.730	0.729	0.732	0.756	0.783	0.783	0.783	0.783
50	Uruguay	0.660	0.691	0.693	0.708	0.709	0.709	0.707	0.710	0.712	0.712
51	Bahamas	0.668	0.708	0.708	0.714	0.714	0.714	0.714	0.714	0.714	0.714
51	Montenegro		0.717	0.730	0.744	0.757	0.768	0.774	0.774	0.774	0.774
53	Belarus		0.717	0.738	0.759	0.779	0.812	0.812	0.817	0.820	0.820
54	Belize	0.603	0.660	0.676	0.678	0.679	0.676	0.675	0.678	0.689	0.689
54	Romania	0.654	0.709	0.717	0.732	0.748	0.754	0.747	0.748	0.748	0.748
55	Libya	0.625	0.660	0.667	0.675	0.683	0.690	0.698	0.698	0.698	0.698
56	Oman		0.527	0.527	0.539	0.556	0.589	0.596	0.603	0.603	0.603
57	Russian Federation	0.723	0.764	0.765	0.769	0.772	0.779	0.780	0.780	0.780	0.780
58	Bulgaria	0.676	0.710	0.713	0.725	0.728	0.735	0.747	0.749	0.749	0.749
59	Barbados	0.684	0.714	0.720	0.725	0.736	0.747	0.743	0.740	0.740	0.740
60	Palau	0.760	0.787	0.787	0.787	0.787	0.787	0.787	0.787	0.787	0.787
61	Antigua and Barbuda							0.687	0.681	0.681	0.681
62	Malaysia	0.602	0.651	0.655	0.659	0.663	0.667	0.671	0.671	0.671	0.671
63	Mauritius	0.574	0.638	0.648	0.656	0.666	0.679	0.686	0.696	0.718	0.718
64	Trinidad and Tobago	0.607	0.684	0.689	0.695	0.700	0.700	0.700	0.700	0.700	0.700
65	Lebanon		0.633	0.622	0.625	0.625	0.628	0.628	0.633	0.631	0.631
65	Panama	0.623	0.653	0.656	0.655	0.658	0.658	0.663	0.657	0.657	0.657
67	Venezuela (Bolivarian Republic of)	0.513	0.594	0.618	0.650	0.670	0.674	0.682	0.682	0.682	0.682
68	Costa Rica	0.584	0.594	0.602	0.611	0.627	0.637	0.640	0.652	0.654	0.654
69	Turkey	0.493	0.531	0.545	0.557	0.563	0.582	0.625	0.648	0.652	0.652
70	Kazakhstan	0.683	0.755	0.759	0.758	0.759	0.755	0.754	0.759	0.762	0.762
71	Seychelles	0.666	0.691	0.689	0.686	0.683	0.633	0.678	0.636	0.636	0.636
71	Mexico	0.544	0.588	0.601	0.604	0.612	0.621	0.631	0.636	0.638	0.638
73	Saint Kitts and Nevis						0.638	0.638	0.638	0.638	0.638
73	Sri Lanka	0.679	0.709	0.715	0.721	0.726	0.729	0.738	0.738	0.738	0.738
75	Iran (Islamic Republic of)	0.526	0.554	0.570	0.587	0.606	0.617	0.625	0.639	0.683	0.683
76	Azerbaijan	0.638	0.667	0.673	0.679	0.679	0.687	0.697	0.700	0.700	0.700

Table F. Mean years of schooling and expected years of schooling

HDI Rank	Countries	2000	2005	2006	2007	2008	2009	2010	2011	2012	2013
77	Serbia	0.686	0.691	0.692	0.693	0.697	0.697	0.695	0.695	0.695	0.695
77	Jordan	0.671	0.707	0.706	0.707	0.714	0.708	0.703	0.700	0.700	0.700
79	Brazil	0.581	0.614	0.627	0.639	0.657	0.658	0.662	0.661	0.661	0.661
79	Grenada							0.724	0.724	0.724	0.724
79	Georgia		0.754	0.751	0.768	0.759	0.770	0.770	0.770	0.770	0.770
82	Peru	0.642	0.635	0.633	0.629	0.631	0.633	0.656	0.660	0.664	0.664
83	Ukraine	0.705	0.765	0.772	0.776	0.779	0.783	0.784	0.790	0.796	0.796
84	The former Yugoslav Republic of Macedonia		0.597	0.603	0.609	0.636	0.639	0.642	0.642	0.642	0.642
86	Bosnia and Herzegovina		0.653	0.651	0.648	0.652	0.649	0.650	0.655	0.655	0.655
87	Armenia	0.668	0.671	0.685	0.701	0.690	0.701	0.701	0.701	0.701	0.701
88	Fiji	0.671	0.694	0.710	0.723	0.735	0.747	0.759	0.767	0.767	0.767
89	Thailand	0.516	0.569	0.559	0.582	0.589	0.601	0.608	0.608	0.608	0.608
90	Tunisia	0.525	0.580	0.588	0.597	0.605	0.610	0.619	0.621	0.621	0.621
91	China	0.478	0.531	0.544	0.561	0.575	0.590	0.599	0.610	0.610	0.610
91	Saint Vincent and the Grenadines						0.657	0.657	0.657	0.657	0.657
93	Dominica	0.617	0.617	0.617	0.617	0.607	0.607	0.607	0.607	0.607	0.607
93	Algeria	0.493	0.563	0.570	0.581	0.598	0.615	0.631	0.643	0.643	0.643
95	Albania	0.565	0.595	0.596	0.598	0.600	0.601	0.602	0.609	0.609	0.609
96	Jamaica	0.588	0.648	0.652	0.656	0.660	0.664	0.668	0.668	0.668	0.668
97	Saint Lucia						0.637	0.626	0.631	0.631	0.631
98	Ecuador	0.547	0.583	0.585	0.587	0.589	0.592	0.594	0.594	0.594	0.594
98	Colombia	0.536	0.574	0.580	0.591	0.603	0.609	0.611	0.613	0.602	0.602
100	Suriname		0.588	0.588	0.588	0.588	0.588	0.588	0.588	0.588	0.588
100	Tonga	0.664	0.710	0.712	0.714	0.716	0.718	0.720	0.720	0.720	0.720
102	Dominican Republic	0.540	0.576	0.579	0.582	0.583	0.583	0.584	0.587	0.590	0.590
103	Maldives	0.484	0.520	0.525	0.531	0.537	0.542	0.548	0.548	0.548	0.548
103	Turkmenistan							0.679	0.679	0.679	0.679
103	Mongolia	0.532	0.627	0.636	0.649	0.661	0.674	0.683	0.688	0.694	0.694
106	Samoa	0.677	0.697	0.702	0.702	0.702	0.702	0.702	0.702	0.702	0.702
107	Palestine (State of)		0.613	0.620	0.639	0.643	0.650	0.652	0.656	0.662	0.662
108	Indonesia	0.521	0.559	0.564	0.565	0.567	0.588	0.594	0.603	0.603	0.603
109	Botswana	0.576	0.607	0.603	0.607	0.611	0.615	0.619	0.619	0.619	0.619
110	Egypt	0.486	0.525	0.534	0.544	0.553	0.562	0.573	0.573	0.573	0.573
111	Paraguay	0.524	0.577	0.565	0.564	0.574	0.583	0.587	0.587	0.587	0.587
112	Gabon	0.545	0.571	0.575	0.578	0.582	0.586	0.589	0.589	0.589	0.589
113	Bolivia (Plurinational State of)	0.620	0.654	0.655	0.656	0.662	0.668	0.674	0.674	0.674	0.674
114	Moldova (Republic of)	0.618	0.646	0.654	0.654	0.657	0.654	0.653	0.656	0.653	0.653
115	El Salvador	0.469	0.528	0.531	0.530	0.531	0.537	0.541	0.548	0.553	0.553
116	Uzbekistan		0.654	0.654	0.654	0.651	0.651	0.651	0.651	0.651	0.651
117	Philippines	0.582	0.608	0.603	0.609	0.614	0.608	0.610	0.610	0.610	0.610
118	Syrian Arab Republic	0.451	0.534		0.539	0.538	0.545	0.548	0.553	0.553	0.553

Table F. Mean years of schooling and expected years of schooling

HDI Rank	Countries	2000	2005	2006	2007	2008	2009	2010	2011	2012	2013
118	South Africa	0.655	0.659	0.661	0.666	0.669	0.682	0.685	0.687	0.695	0.695
120	Iraq	0.401	0.456	0.458	0.460	0.462	0.465	0.467	0.467	0.467	0.467
121	Vietnam	0.434	0.470	0.478	0.486	0.494	0.502	0.509	0.513	0.513	0.513
121	Guyana	0.553	0.587	0.581	0.574	0.575	0.573	0.576	0.582	0.582	0.582
123	Cabo Verde	0.442	0.442	0.461	0.458	0.464	0.467	0.472	0.483	0.483	0.483
124	Micronesia (Federated States of)							0.611	0.611	0.611	0.611
125	Guatemala	0.365	0.399	0.409	0.428	0.439	0.451	0.462	0.473	0.484	0.484
125	Kyrgyzstan	0.634	0.656	0.659	0.660	0.658	0.653	0.651	0.656	0.656	0.656
127	Namibia	0.508	0.515	0.514	0.515	0.517	0.519	0.520	0.520	0.520	0.520
128	Timor-Leste	0.362	0.379	0.399	0.419	0.439	0.459	0.472	0.472	0.472	0.472
129	Morocco	0.346	0.408	0.411	0.422	0.431	0.440	0.454	0.468	0.468	0.468
129	Honduras	0.419	0.458	0.466	0.473	0.483	0.492	0.502	0.507	0.505	0.505
131	Vanuatu					0.596	0.596	0.596	0.596	0.596	0.596
132	Nicaragua	0.425	0.463	0.467	0.471	0.476	0.480	0.484	0.484	0.484	0.484
133	Kiribati							0.602	0.602	0.602	0.602
133	Tajikistan	0.601	0.630	0.635	0.631	0.639	0.638	0.639	0.639	0.639	0.639
135	India	0.355	0.409	0.420	0.430	0.442	0.445	0.456	0.473	0.473	0.473
136	Bhutan							0.410	0.421	0.421	0.421
136	Cambodia	0.400	0.478	0.485	0.491	0.494	0.495	0.495	0.495	0.495	0.495
138	Ghana	0.433	0.460	0.477	0.496	0.516	0.522	0.535	0.544	0.553	0.553
139	Lao People's Democratic Republic	0.352	0.385	0.390	0.395	0.403	0.414	0.422	0.436	0.436	0.436
140	Congo	0.410	0.459	0.467	0.475	0.483	0.491	0.499	0.505	0.511	0.511
141	Zambia	0.488	0.538	0.546	0.554	0.561	0.569	0.577	0.584	0.591	0.591
142	Bangladesh	0.330	0.381	0.388	0.395	0.397	0.415	0.433	0.447	0.447	0.447
142	Sao Tome and Principe	0.392	0.422	0.430	0.439	0.441	0.458	0.455	0.462	0.469	0.469
144	Equatorial Guinea	0.415	0.415	0.415	0.415	0.415	0.415	0.415	0.415	0.415	0.415
145	Nepal	0.337	0.366	0.384	0.387	0.398	0.418	0.444	0.452	0.452	0.452
146	Pakistan	0.261	0.331	0.326	0.338	0.338	0.352	0.359	0.366	0.372	0.372
147	Kenya	0.431	0.467	0.472	0.491	0.502	0.512	0.515	0.515	0.515	0.515
148	Swaziland	0.459	0.494	0.514	0.523	0.531	0.539	0.547	0.551	0.551	0.551
149	Angola	0.299	0.365	0.380	0.395	0.410	0.426	0.441	0.474	0.474	0.474
150	Myanmar	0.310	0.343	0.351	0.360	0.363	0.367	0.371	0.371	0.371	0.371
151	Rwanda	0.278	0.324	0.355	0.356	0.354	0.366	0.375	0.387	0.478	0.478
152	Nigeria		0.415	0.417	0.419	0.421	0.423	0.425	0.425	0.425	0.425
152	Cameroon	0.357	0.408	0.406	0.429	0.446	0.463	0.480	0.486	0.486	0.486
154	Yemen	0.257	0.304	0.307	0.310	0.313	0.321	0.328	0.339	0.339	0.339
155	Madagascar	0.398	0.422	0.431	0.433	0.442	0.456	0.457	0.457	0.458	0.458
156	Zimbabwe	0.471	0.483	0.487	0.490	0.493	0.497	0.500	0.500	0.500	0.500
157	Papua New Guinea	0.285	0.326	0.335	0.344	0.353	0.361	0.370	0.376	0.376	0.376
157	Solomon Islands	0.333	0.383	0.400	0.405	0.405	0.405	0.405	0.405	0.405	0.405
159	Tanzania (United Republic of)	0.325	0.369	0.379	0.389	0.399	0.401	0.403	0.426	0.426	0.426

Table F. Mean years of schooling and expected years of schooling

HDI Rank	Countries	2000	2005	2006	2007	2008	2009	2010	2011	2012	2013
159	Comoros		0.402	0.409	0.416	0.423	0.430	0.437	0.443	0.450	0.450
161	Mauritania	0.280	0.311	0.322	0.325	0.319	0.336	0.338	0.341	0.352	0.352
162	Lesotho	0.449	0.474	0.477	0.483	0.489	0.495	0.500	0.502	0.504	0.504
163	Senegal	0.266	0.312	0.324	0.329	0.343	0.357	0.364	0.368	0.368	0.368
164	Uganda	0.426	0.449	0.446	0.452	0.466	0.475	0.479	0.479	0.479	0.479
165	Benin	0.269	0.339	0.352	0.364	0.377	0.390	0.403	0.414	0.414	0.414
166	Sudan	0.206	0.261	0.272	0.283	0.293	0.304	0.306	0.306	0.306	0.306
166	Togo	0.407	0.446	0.460	0.449	0.466	0.483	0.501	0.514	0.514	0.514
168	Haiti	0.331	0.355	0.359	0.363	0.367	0.371	0.374	0.374	0.374	0.374
169	Afghanistan	0.226	0.297	0.309	0.321	0.332	0.344	0.357	0.365	0.365	0.365
170	Djibouti		0.248	0.253	0.267	0.278	0.284	0.295	0.306	0.306	0.306
171	Côte d'Ivoire	0.302	0.343	0.351	0.359	0.368	0.376	0.384	0.389	0.389	0.389
172	Gambia	0.256	0.307	0.318	0.329	0.340	0.343	0.346	0.346	0.346	0.346
173	Ethiopia	0.169	0.233	0.252	0.281	0.301	0.305	0.300	0.314	0.317	0.317
174	Malawi	0.388	0.380	0.388	0.387	0.403	0.419	0.434	0.440	0.440	0.440
175	Liberia	0.344	0.349	0.353	0.357	0.360	0.364	0.367	0.367	0.367	0.367
176	Mali	0.177	0.239	0.251	0.263	0.276	0.286	0.294	0.301	0.305	0.305
177	Guinea-Bissau		0.314	0.325	0.325	0.325	0.325	0.325	0.325	0.325	0.325
178	Mozambique	0.222	0.324	0.334	0.353	0.366	0.374	0.373	0.372	0.372	0.372
179	Guinea		0.253	0.267	0.278	0.281	0.281	0.286	0.292	0.294	0.294
180	Burundi	0.187	0.242	0.281	0.303	0.327	0.351	0.370	0.370	0.370	0.370
181	Burkina Faso		0.172	0.178	0.192	0.206	0.217	0.228	0.242	0.250	0.250
182	Eritrea							0.228	0.228	0.228	0.228
183	Sierra Leone	0.274	0.286	0.288	0.290	0.292	0.294	0.296	0.305	0.305	0.305
184	Chad	0.186	0.203	0.210	0.218	0.225	0.236	0.236	0.256	0.256	0.256
185	Central African Republic	0.248	0.280	0.286	0.291	0.296	0.302	0.311	0.318	0.318	0.318
186	Congo (Democratic Republic of the)	0.297	0.316	0.320	0.323	0.346	0.349	0.355	0.361	0.372	0.372
187	Niger	0.118	0.149	0.152	0.159	0.165	0.172	0.184	0.192	0.198	0.198

Table G. Country rank per year for Mean Years of Schooling and Expected Years of Schooling

HDI Rank	Countries	2000	2005	2006	2007	2008	2009	2010	2011	2012	2013
1	Norway	156	174	173	173	173	176	185	185	185	185
2	Australia	158	172	171	172	174	178	187	187	187	187
3	Switzerland	145	156	155	157	158	161	170	170	170	170
4	Netherlands	150	161	162	166	167	168	178	184	184	184
5	United States	154	171	169	170	171	174	183	183	183	183
6	Germany	142	167	170	169	170	172	181	181	181	181
7	New Zealand	157	173	172	174	175	177	186	186	186	186
8	Canada	147	165	163	163	162	164	173	172	172	172

Table G. Country rank per year for Mean Years of Schooling and Expected Years of Schooling

HDI Rank	Countries	2000	2005	2006	2007	2008	2009	2010	2011	2012	2013
9	Singapore	87	110	112	116	124	127	143	141	142	147
10	Denmark	146	168	167	168	168	171	179	179	179	179
11	Ireland	153	169	168	171	172	175	184	182	182	182
12	Sweden	155	158	156	156	156	160	169	169	169	169
13	Iceland	141	157	157	158	159	162	171	171	171	171
14	United Kingdom	152	170	165	162	166	170	180	175	175	175
15	Hong Kong, China (SAR)	101	115	117	123	134	139	144	144	147	146
15	Korea (Republic of)	144	159	158	159	164	166	175	177	177	177
17	Japan	138	147	147	147	148	150	160	162	162	162
18	Liechtenstein							141	143	143	142
19	Israel	149	162	159	160	160	163	172	173	173	173
20	France	135	149	148	149	152	154	163	164	166	166
21	Luxembourg	131	139	136	135	133	136	147	145	144	143
21	Austria	130	140	139	140	141	145	158	158	157	157
21	Belgium	151	150	149	148	149	152	162	163	163	163
24	Finland	136	155	154	155	154	156	166	166	165	165
25	Slovenia	143	160	161	161	165	167	177	176	176	176
26	Italy	124	143	142	143	143	146	154	155	155	155
27	Spain	128	138	137	137	139	140	152	157	156	156
28	Czech Republic	140	164	160	165	161	169	176	178	178	178
29	Greece	120	152	152	146	147	149	159	159	159	159
30	Brunei Darussalam	100	113	111	112	108	112	113	115	117	117
31	Qatar	89	114	114	117	119	115	115	110	115	115
32	Cyprus	116	136	135	136	140	143	148	150	151	151
33	Estonia	148	166	164	164	163	165	174	174	174	174
34	Saudi Arabia	75	90	90	90	92	103	121	128	132	132
35	Poland	139	153	151	152	153	155	167	168	168	168
35	Lithuania	137	163	166	167	169	173	182	180	180	180
37	Andorra							107	108	107	107
37	Slovakia	132	146	146	150	151	153	161	160	160	160
39	Malta	90	117	110	111	113	122	134	134	135	135
40	United Arab Emirates	86	99	99	104	104	107	109	111	109	109
41	Portugal	106	109	107	108	114	119	130	133	134	134
41	Chile	102	123	122	126	132	131	137	138	139	139
43	Hungary	133	151	150	151	150	151	164	161	161	161
44	Bahrain	107	131	121	120	120	125	132	131	129	129
44	Cuba	108	137	143	153	155	158	157	147	138	138
46	Kuwait	97	87	85	86	86	86	92	92	94	94
47	Croatia	105	126	130	132	131	132	146	149	149	149
48	Latvia	126	154	153	154	157	159	168	165	164	164
49	Argentina	127	135	132	131	128	138	153	153	153	153

Table G. Country rank per year for Mean Years of Schooling and Expected Years of Schooling

HDI Rank	Countries	2000	2005	2006	2007	2008	2009	2010	2011	2012	2013
50	Uruguay	109	118	119	122	121	124	129	129	127	127
51	Bahamas	112	125	124	125	123	126	131	130	128	128
51	Montenegro		133	133	134	136	141	150	151	150	150
53	Belarus		134	134	139	144	157	165	167	167	167
54	Belize	85	108	109	109	110	110	111	113	116	116
54	Romania	103	127	129	133	135	135	140	139	140	140
55	Libya	95	107	106	107	111	116	124	122	121	121
56	Oman		62	61	62	64	72	75	76	77	77
57	Russian Federation	129	144	141	142	142	144	151	152	152	152
58	Bulgaria	117	129	127	129	127	130	139	140	141	141
59	Barbados	122	132	131	130	130	133	138	137	137	137
60	Palau	134	148	145	145	146	148	156	154	154	154
61	Antigua and Barbuda							120	116	112	112
62	Malaysia	84	100	102	103	103	105	108	109	108	108
63	Mauritius	77	96	95	100	105	111	119	121	130	130
64	Trinidad and Tobago	88	116	116	115	117	118	125	125	124	124
65	Lebanon		94	89	88	87	87	89	87	86	86
65	Panama	94	101	103	99	99	101	105	104	102	102
67	Venezuela (Bolivarian Republic of)	62	82	87	96	107	109	116	117	113	113
68	Costa Rica	81	81	81	85	88	91	95	98	98	98
69	Turkey	59	64	65	66	66	66	86	96	96	96
70	Kazakhstan	121	142	140	138	138	137	142	142	145	144
71	Seychelles	111	120	115	113	112	88	112	89	88	88
71	Mexico	71	79	80	81	84	85	90	88	89	89
73	Saint Kitts and Nevis						93	93	90	90	90
73	Sri Lanka	119	128	128	127	126	129	136	136	136	136
75	Iran (Islamic Republic of)	67	68	70	75	81	84	87	91	114	114
76	Azerbaijan	98	111	108	110	109	114	123	124	123	123
77	Serbia	123	119	118	114	116	117	122	120	119	119
77	Jordan	114	124	123	121	122	123	128	123	122	122
79	Brazil	79	89	91	92	97	102	104	106	103	103
79	Grenada							135	135	133	133
79	Georgia		141	138	141	137	142	149	148	148	148
82	Peru	99	95	92	89	89	89	102	105	105	105
83	Ukraine	125	145	144	144	145	147	155	156	158	158
84	The former Yugoslav Republic of Macedonia		84	83	83	90	94	96	94	92	92
86	Bosnia and Herzegovina		102	96	94	95	95	97	99	99	99
87	Armenia	113	112	113	118	115	120	126	126	125	125
88	Fiji	115	121	125	128	129	134	145	146	146	145
89	Thailand	63	71	67	74	74	77	80	78	79	79
90	Tunisia	66	76	77	79	80	81	84	85	85	85

Table G. Country rank per year for Mean Years of Schooling and Expected Years of Schooling

HDI Rank	Countries	2000	2005	2006	2007	2008	2009	2010	2011	2012	2013
91	China	55	65	64	67	70	73	76	80	81	81
91	Saint Vincent and the Grenadines						100	103	103	101	101
93	Dominica	91	91	86	87	82	78	79	77	78	78
93	Algeria	60	70	71	72	77	83	91	95	93	93
95	Albania	76	83	79	80	78	76	77	79	80	80
96	Jamaica	82	98	97	102	100	104	106	107	106	106
97	Saint Lucia						90	88	86	87	87
98	Ecuador	73	77	76	76	75	74	72	72	72	72
98	Colombia	69	73	74	78	79	80	82	83	75	75
100	Suriname		80	78	77	73	71	70	70	68	68
100	Tonga	110	130	126	124	125	128	133	132	131	131
102	Dominican Republic	70	74	73	73	72	68	68	68	70	70
103	Maldives	56	60	60	61	61	61	63	61	60	60
103	Turkmenistan							114	114	111	111
103	Mongolia	68	92	94	95	101	108	117	119	118	118
106	Samoa	118	122	120	119	118	121	127	127	126	126
107	Palestine (State of)		88	88	93	93	96	100	100	104	104
108	Indonesia	64	69	68	69	67	70	73	75	76	76
109	Botswana	78	85	82	82	83	82	85	84	84	84
110	Egypt	57	61	63	64	63	63	65	65	65	65
111	Paraguay	65	75	69	68	68	67	69	69	67	67
112	Gabon	72	72	72	71	71	69	71	71	69	69
113	Bolivia (Plurinational State of)	93	103	101	101	102	106	110	112	110	110
114	Moldova (Republic of)	92	97	100	98	96	99	101	101	97	97
115	El Salvador	53	63	62	60	60	59	61	62	64	64
116	Uzbekistan		104	98	97	94	97	99	97	95	95
117	Philippines	80	86	84	84	85	79	81	81	82	82
118	Syrian Arab Republic	51	66		63	62	62	64	64	63	63
118	South Africa	104	106	105	106	106	113	118	118	120	120
120	Iraq	39	48	46	48	45	45	45	41	39	39
121	Vietnam	48	54	55	53	54	55	57	56	56	56
121	Guyana	74	78	75	70	69	65	66	66	66	66
123	Cabo Verde	49	45	48	47	46	46	46	48	47	47
124	Micronesia (Federated States of)							83	82	83	83
125	Guatemala	34	36	38	40	40	40	44	45	48	48
125	Kyrgyzstan	96	105	104	105	98	98	98	102	100	100
127	Namibia	61	59	58	58	58	57	59	59	59	59
128	Timor-Leste	33	31	34	37	39	43	47	43	42	42
129	Morocco	29	38	39	39	38	38	40	42	40	40
129	Honduras	43	49	49	50	51	51	56	55	54	54
131	Vanuatu					76	75	74	73	73	73

Table G. Country rank per year for Mean Years of Schooling and Expected Years of Schooling

HDI Rank	Countries	2000	2005	2006	2007	2008	2009	2010	2011	2012	2013
132	Nicaragua	44	52	51	49	49	48	50	49	49	49
133	Kiribati							78	74	74	74
133	Tajikistan	83	93	93	91	91	92	94	93	91	91
135	India	31	40	42	42	42	39	42	44	43	43
136	Bhutan							31	31	30	30
136	Cambodia	38	56	56	56	55	53	51	51	51	51
138	Ghana	47	51	53	57	57	58	60	60	62	62
139	Lao People's Democratic Republic	30	35	33	33	32	30	33	34	33	33
140	Congo	41	50	50	51	50	50	52	54	55	55
141	Zambia	58	67	66	65	65	64	67	67	71	71
142	Bangladesh	24	33	32	32	28	31	35	37	35	35
142	Sao Tome and Principe	36	43	43	44	41	42	41	40	41	41
144	Equatorial Guinea	42	41	40	35	35	32	32	30	29	29
145	Nepal	27	29	30	28	29	33	39	38	37	37
146	Pakistan	12	22	19	19	16	18	18	18	21	21
147	Kenya	46	53	52	55	56	56	58	58	58	58
148	Swaziland	52	58	59	59	59	60	62	63	61	61
149	Angola	20	28	29	31	34	36	38	46	44	44
150	Myanmar	22	25	23	25	23	23	23	22	20	20
151	Rwanda	16	19	26	22	21	22	26	26	45	45
152	Nigeria		42	41	38	36	35	34	32	31	31
152	Cameroon	32	39	36	41	44	44	49	50	50	50
154	Yemen	11	13	12	12	11	11	13	13	13	13
155	Madagascar	37	44	44	43	43	41	43	39	38	38
156	Zimbabwe	54	57	57	54	53	54	53	52	52	52
157	Papua New Guinea	18	21	21	20	20	20	21	25	25	25
157	Solomon Islands	26	34	35	34	33	29	30	28	27	27
159	Tanzania (United Republic of)	23	30	28	30	30	28	29	33	32	32
159	Comoros		37	37	36	37	37	37	36	36	36
161	Mauritania	17	15	16	15	12	13	14	14	15	15
162	Lesotho	50	55	54	52	52	52	54	53	53	53
163	Senegal	13	16	17	18	18	19	19	20	18	18
164	Uganda	45	47	45	46	47	47	48	47	46	46
165	Benin	14	23	24	27	27	27	28	29	28	28
166	Sudan	6	9	8	8	8	9	10	9	9	9
166	Togo	40	46	47	45	48	49	55	57	57	57
168	Haiti	25	27	27	26	25	24	25	24	24	24
169	Afghanistan	8	12	13	13	15	15	17	17	16	16
170	Djibouti		7	6	5	5	5	7	8	8	8
171	Côte d'Ivoire	21	24	22	24	26	26	27	27	26	26
172	Gambia	10	14	14	17	17	14	15	15	14	14

Table G. Country rank per year for Mean Years of Schooling and Expected Years of Schooling

HDI Rank	Countries	2000	2005	2006	2007	2008	2009	2010	2011	2012	2013
173	Ethiopia	2	4	5	7	10	10	9	10	10	10
174	Malawi	35	32	31	29	31	34	36	35	34	34
175	Liberia	28	26	25	23	22	21	20	19	17	17
176	Mali	3	5	4	4	4	6	6	6	7	7
177	Guinea-Bissau		17	18	16	13	12	12	12	12	12
178	Mozambique	7	20	20	21	24	25	24	23	23	23
179	Guinea		8	7	6	6	4	5	5	5	5
180	Burundi	5	6	9	11	14	17	22	21	19	19
181	Burkina Faso		2	2	2	2	2	3	3	3	3
182	Eritrea							2	2	2	2
183	Sierra Leone	15	11	11	9	7	7	8	7	6	6
184	Chad	4	3	3	3	3	3	4	4	4	4
185	Central African Republic	9	10	10	10	9	8	11	11	11	11
186	Congo (Democratic Republic of the)	19	18	15	14	19	16	16	16	22	22
187	Niger	1	1	1	1	1	1	1	1	1	1
	**Total...**	**158**	**174**	**173**	**174**	**175**	**178**	**187**	**187**	**187**	**187**
	**Normalized value**	0.234	0.253	0.254	0.247	0.246	0.230	0.230	0.209	0.203	0.203

International Human Development Indicators
Accessed: 7/14/2014,2:06 PM from: http://hdr.undp.org
Expenditure on education, Public (% of GDP) (%)
Total public expenditure (current and capital) on education expressed as a percentage of GDP.

Source: World Bank (2013). "World Development Indicators 2013." Washington, D.C.: World Bank. http://data.worldbank.org. Accessed October, 2013. http://data.worldbank.org/data-catalog/world-development-indicators

Data in the tables are those available to the Human Development Report Office as of 15 November, 2013, unless otherwise specified.

Table H. Total public expenditure (current and capital) on education expressed as a percentage of GDP

HDI Rank	Countries	1980	1985	2000	2005	2006	2007	2008	2009	2011	2012
1	Norway	5.8	5.4	6.6	7.0	6.5	6.7	6.4	7.3		6.9
2	Australia	5.7	5.4	4.9	4.9	4.8	4.7	4.6	5.1		5.1
3	Switzerland	4.6	4.5	5.1	5.5	5.3	5.0	5.1	5.4		5.4
4	Netherlands	6.4	5.9	5.0	5.5	5.5	5.3	5.5	5.9		6.0
5	United States				5.3	5.6	5.5	5.5	5.4		5.6
6	Germany					4.4	4.5	4.6	5.1		5.1
7	New Zealand	4.3	3.6		6.4	6.0	6.0	5.6	6.4		7.2
8	Canada	6.6	6.4	5.6	4.9		4.9	4.8	5.0		5.5
9	Singapore	2.6		3.4				2.8	3.3	3.2	3.3
10	Denmark	6.0		8.3	8.3	8.0	7.8	7.7	8.7		8.7
11	Ireland	5.5	5.4	4.2	4.7	4.7	4.9	5.7	6.5		6.5
12	Sweden	7.0		7.2	6.9	6.7	6.6	6.8	7.3		7.0
13	Iceland			6.7	7.6	7.5	7.4	7.6	7.8		7.8
14	United Kingdom	5.3	4.8	4.5	5.4	5.5	5.4	5.4	5.6		5.6
15	Hong Kong, China (SAR)	2.2	2.6		4.1	3.8	3.5	3.3	4.4	3.4	3.4
15	Korea (Republic of)	3.5	4.2		4.1	4.2	4.2	4.8	5.0		5.0
17	Japan	5.2		3.6	3.5	3.5	3.5	3.4			3.8
18	Liechtenstein					2.1	1.9	2.1			2.1
19	Israel	8.6	9.0	6.5	6.1	6.1	5.9	5.9	5.8		6.0
20	France	4.4	4.9	5.7	5.7	5.6	5.6	5.6	5.9		5.9
21	Austria	5.1	5.4	5.7	5.4	5.4	5.3	5.5	6.0		6.0
21	Belgium	5.4			5.9	6.0	6.0	6.4	6.6		6.6
21	Luxembourg	4.6									
24	Finland	4.9	5.1	5.9	6.3	6.2	5.9	6.1	6.8		6.8
25	Slovenia				5.7	5.7	5.2	5.2	5.7		5.7
26	Italy		4.9	4.4	4.4	4.7	4.3	4.6	4.7		4.5
27	Spain			4.3	4.2	4.3	4.3	4.6	5.0		5.0
28	Czech Republic			3.8	4.1	4.4	4.1	3.9	4.4		4.2
29	Greece			3.4	4.1						4.1
30	Brunei Darussalam	1.2		3.7						3.7	3.3
31	Qatar							2.5			2.5
32	Cyprus	3.4	3.6	5.3	6.9	7.0	6.9	7.4	7.9		7.3
33	Estonia			5.3	4.9		4.7	5.6	6.1		5.7
34	Saudi Arabia			5.9	5.7	6.2	6.4	5.6			5.6

Table H. Total public expenditure (current and capital) on education expressed as a percentage of GDP

HDI Rank	Countries	1980	1985	2000	2005	2006	2007	2008	2009	2011	2012
35	Lithuania				4.9	4.8	4.7	4.9	5.7		5.4
35	Poland			5.0	5.5	5.3	4.9	5.1	5.1		5.2
37	Andorra				2.0	2.7	2.6	3.2	3.2	3.0	3.0
37	Slovakia			3.9	3.8	3.8	3.6	3.6	4.1		4.2
39	Malta		3.2				6.3	5.8	5.4		5.4
40	United Arab Emirates			1.3							
41	Chile	4.4		3.7	3.2	3.0	3.2	3.8	4.2	4.1	4.1
41	Portugal	3.1	3.3	5.2	5.2	5.1	5.1	4.9	5.8		5.8
43	Hungary	4.5	4.7	5.0	5.5	5.4	5.3	5.1	5.1		4.9
44	Bahrain					3.4	3.0	2.9			2.9
44	Cuba	8.4		7.7	10.6	9.1	11.9	14.1	13.1		12.9
46	Kuwait	2.8	5.0		4.7	3.8					3.8
47	Croatia						4.0	4.3	4.4		4.3
48	Latvia	3.3	3.4	5.4		5.1	5.0	5.7	5.6		5.0
49	Argentina	2.6	1.4	4.6		4.5	4.9	5.4	6.0		5.8
50	Uruguay	2.2	2.6	2.4	2.7	2.9					2.9
51	Bahamas			2.8							
51	Montenegro										
53	Belarus			6.2	5.9	6.1	5.2		4.5	5.2	5.2
54	Romania			2.9	3.5		4.3		4.2		4.2
55	Libya										
56	Oman			3.1	3.5	3.9			4.3		4.3
57	Russian Federation			2.9	3.8	3.9		4.1			4.1
58	Bulgaria	3.5			4.2	4.0	3.9	4.4	4.6		4.1
59	Barbados			5.6	6.9		6.9	6.7	6.7		7.5
60	Palau			9.8							
61	Antigua and Barbuda								2.5		2.5
62	Malaysia	5.6	6.0	6.0		4.5	4.4	4.0	6.0		5.1
63	Mauritius		3.9	3.8	4.2	3.8	3.4	3.2	3.2		3.7
64	Trinidad and Tobago	3.8	5.8	3.8							
65	Lebanon			1.9	2.6	2.7	2.6	2.0	1.8	1.6	1.6
65	Panama	4.3	4.3	5.0				3.8		4.1	4.1
67	Venezuela (Bolivarian Republic of)	3.9				3.7	3.6				3.6
68	Costa Rica	7.3		4.4		4.7	4.7	5.0	6.3		6.3
69	Turkey		1.7	2.6		2.9					2.9
70	Kazakhstan			3.3	2.3	2.6	2.8	2.6	3.1		3.1
71	Mexico			4.9	5.0	4.8	4.8	4.9	5.3		5.3
71	Seychelles		10.2			4.8					4.8
73	Saint Kitts and Nevis		5.6	4.8	3.9	4.2	4.2				4.2
73	Sri Lanka	2.7	2.6						2.1	2.0	2.0
75	Iran (Islamic Republic of)		3.7	4.4	4.7	5.1	5.5	4.8	4.7		4.7
76	Azerbaijan			3.9	3.0	2.6	2.5	2.4	3.2		2.8
77	Jordan										
77	Serbia						4.7	4.9	5.0		4.7
79	Brazil			4.0	4.5	5.0	5.1	5.4	5.6		5.8

Table H. Total public expenditure (current and capital) on education expressed as a percentage of GDP

HDI Rank	Countries	1980	1985	2000	2005	2006	2007	2008	2009	2011	2012
79	Georgia			2.2	2.5	3.0	2.7	2.9	3.2	2.7	2.7
79	Grenada										
82	Peru				2.7	2.5	2.5	2.7	3.0	2.6	2.6
83	Ukraine			4.2	6.1	6.2	5.3				5.3
84	Belize			5.0				5.7	6.1		6.6
84	The former Yugoslav Republic of Macedonia										
86	Bosnia and Herzegovina										
87	Armenia			2.8	2.7	2.7	3.0	3.2	3.8	3.1	3.1
88	Fiji			5.9	5.1	5.6	5.8	4.2	4.5	4.1	4.1
89	Thailand	2.6		5.4	4.2	4.3	3.8	3.8	4.1	5.8	5.8
90	Tunisia	5.2	5.5	6.2	6.5	6.4	6.5	6.3	6.5		6.2
91	China	1.9	2.0								
91	Saint Vincent and the Grenadines			7.9	6.3		5.7		5.7		5.1
93	Algeria	6.6						4.3			4.3
93	Dominica										3.5
95	Albania			3.2	3.2	3.1	3.3				3.3
96	Jamaica		5.4	5.0	4.6		5.5	6.2	6.2		6.4
97	Saint Lucia			6.7	5.3	5.9		5.3	3.8	4.4	4.4
98	Colombia	1.7	2.7	3.5	4.0	3.9	4.1	3.9	4.7	4.5	4.5
98	Ecuador		7.6	1.3						5.2	5.2
100	Suriname										
100	Tonga			4.9							
102	Dominican Republic		1.2	1.9			2.2				2.2
103	Maldives				5.9	5.6	5.4	5.4	7.8	7.2	7.2
103	Mongolia			5.6			4.7		5.1	5.5	5.5
103	Turkmenistan										
106	Samoa			4.0				5.8			5.8
107	Palestine, State of										
108	Indonesia				2.9	3.6	3.0	2.9	3.5	2.8	2.8
109	Botswana	5.5	5.7		9.5		8.0		7.8		7.8
110	Egypt	4.2			4.8	4.0	3.7	3.8			3.8
111	Paraguay		1.4	5.3			4.0				4.1
112	Gabon			3.8							
113	Bolivia (Plurinational State of)			5.5		6.3		7.0	8.1		7.6
114	Moldova (Republic of)			4.5	7.2	7.5	8.3	8.2	9.5	8.6	8.6
115	El Salvador			2.5	2.7	3.0	3.1	3.7		3.4	3.4
116	Uzbekistan										
117	Philippines	1.7		3.3	2.4	2.5	2.6	2.7	2.7		2.7
118	South Africa			5.6	5.3	5.3	5.2	5.1	5.5		6.0
118	Syrian Arab Republic	4.6	6.1			5.3	4.9	4.6	5.1		5.1
120	Iraq	2.3									
121	Guyana			8.5	8.1	5.1	3.8		3.4	3.6	3.6
121	Viet Nam							5.3			6.6
123	Cabo Verde					6.5	6.1	6.3	5.7		5.6

Table H. Total public expenditure (current and capital) on education expressed as a percentage of GDP

HDI Rank	Countries	1980	1985	2000	2005	2006	2007	2008	2009	2011	2012
124	Micronesia (Federated States of)			6.7							
125	Guatemala					3.0	3.0	3.2			2.8
125	Kyrgyzstan			3.5	4.9	5.5	6.5	5.9	6.2		5.8
127	Namibia			7.9		6.0		6.4			8.4
128	Timor-Leste							8.5	11.9	10.1	10.1
129	Honduras	3.0									
129	Morocco	5.9	5.9	5.8		5.5		5.6	5.4		5.4
131	Vanuatu			7.0				6.6	5.2		5.2
132	Nicaragua	3.3		3.0							4.7
133	Kiribati			11.0							
133	Tajikistan			2.3	3.5	3.4	3.4	3.5	4.1	3.9	3.9
135	India			4.3	3.1	3.1			3.2		3.3
136	Bhutan			5.5	7.1			4.8	4.6	4.7	4.7
136	Cambodia			1.7			1.6				2.6
138	Ghana	2.7			7.4	5.3	5.5	5.8	5.3	8.2	8.2
139	Lao People's Democratic Republic			1.5	2.4	3.0	3.1	2.3			3.3
140	Congo	6.4			1.8						6.2
141	Zambia	4.1	4.1	2.0	2.0		1.5	1.3			1.3
142	Bangladesh	0.9	1.2	2.4		2.5	2.6	2.4	2.2		2.2
142	Sao Tome and Principe										
144	Equatorial Guinea			0.7							
145	Nepal			3.0	3.4	3.6	3.5	3.8	4.7		4.7
146	Pakistan	2.1	2.4	1.8	2.3	2.6	2.8	2.9	2.7		2.4
147	Kenya	5.4	5.2	5.2	7.3	7.0					6.7
148	Swaziland	5.3		5.5	7.4	8.1		7.5	7.1	7.8	7.8
149	Angola		4.5	2.6	2.8	2.9					3.5
150	Myanmar			0.6						0.8	0.8
151	Rwanda	2.7		4.1			4.3	3.9		4.8	4.8
152	Cameroon			1.9	3.1	3.0	3.4	2.9	3.6	3.2	3.2
152	Nigeria										
154	Yemen			9.7				5.2			5.2
155	Madagascar	4.3	2.8	2.9	3.8	3.3	3.4	2.9	3.2	2.8	2.8
156	Zimbabwe	2.5	7.4								2.5
157	Papua New Guinea										
157	Solomon Islands						5.9	6.1	8.0		7.3
159	Comoros							7.6			7.6
159	Tanzania (United Republic of)							6.8			6.2
161	Mauritania					2.5		4.0		3.7	3.7
162	Lesotho		7.8	11.5	14.8	13.9		13.0			13.0
163	Senegal			3.2	5.1	4.8		5.0	5.6		5.6
164	Uganda		3.4	2.5					3.3	3.2	3.3
165	Benin			3.1	4.0	3.8	3.5	4.1	4.6		5.3
166	Sudan										
166	Togo			4.5	3.4	3.7	3.7	3.4	4.1	4.6	4.6
168	Haiti										

Table H. Total public expenditure (current and capital) on education expressed as a percentage of GDP

HDI Rank	Countries	1980	1985	2000	2005	2006	2007	2008	2009	2011	2012
169	Afghanistan	1.8									
170	Djibouti			9.7	8.4	8.3	8.4				8.4
171	Côte d'Ivoire	6.9		3.8	4.3	4.2	4.4	4.6			4.6
172	Gambia		4.3	1.5				3.5	3.1	3.9	3.9
173	Ethiopia		2.4	3.9		5.5	5.5	5.4	4.6		4.7

Table I. Country rank per year for Expenditure on education, Public (% of GDP)

HDI Rank	Countries	1980	1985	2000	2005	2006	2007	2008	2009	2011	2012
1	Norway	54	37	108	90	96	98	102	96		139
2	Australia	53	37	72	56	57	54	51	54		89
3	Switzerland	39	28	82	71	68	66	67	63		102
4	Netherlands	58	46	76	71	75	73	81	78		123
5	United States				66	80	79	81	63		109
6	Germany					49	52	51	54		89
7	New Zealand	33	20		85	86	90	84	89		141
8	Canada	60	50	94	56		61	57	50		107
9	Singapore	14		41				14	21	15	35
10	Denmark	56		117	98	103	102	114	104		155
11	Ireland	50	37	60	52	54	61	89	90		133
12	Sweden	63		113	87	98	97	107	96		140
13	Iceland			109	96	101	101	112	98		148
14	United Kingdom	46	31	67	69	75	77	76	68		109
15	Hong Kong, China (SAR)	9	11		38	34	28	25	35	18	41
15	Korea (Republic of)	27	25		38	44	44	57	50		85
17	Japan	44		45	27	29	28	27			50
18	Liechtenstein					2	4	4			8
19	Israel	66	54	107	81	89	87	95	76		123
20	France	36	32	98	75	80	84	84	78		122
21	Austria	43	37	98	69	73	73	81	80		123
21	Belgium	48			78	86	90	102	92		134
21	Luxembourg	39									
24	Finland	42	35	101	83	91	87	97	94		138
25	Slovenia				75	84	70	71	72		114
26	Italy		32	64	48	54	46	51	46		70
27	Spain			62	43	47	46	51	50		85
28	Czech Republic			49	38	49	42	40	35		62
29	Greece			41	38						55
30	Brunei Darussalam	2		47						22	35
31	Qatar							10			13

Table I. Country rank per year for Expenditure on education, Public (% of GDP)

HDI Rank	Countries	1980	1985	2000	2005	2006	2007	2008	2009	2011	2012
32	Cyprus	25	20	86	87	99	99	110	101		143
33	Estonia			86	56		54	84	83		114
34	Saudi Arabia			101	75	91	94	84			109
35	Lithuania				56	57	54	61	72		102
35	Poland			76	71	68	61	67	54		94
37	Andorra				4	11	8	21	14	12	30
37	Slovakia			54	32	34	32	31	28		62
39	Malta		16				93	92	63		102
40	United Arab Emirates			3							
41	Chile	36		47	23	17	22	34	32	26	55
41	Portugal	22	17	83	64	64	68	61	76		116
43	Hungary	38	30	76	71	73	73	67	54		84
44	Bahrain					27	16	15			27
44	Cuba	65		114	101	106	106	118	107		157
46	Kuwait	20	34		52	34					50
47	Croatia						39	48	35		66
48	Latvia	23	18	89		64	66	89	68		85
49	Argentina	14	4	70		51	61	76	80		116
50	Uruguay	9	11	17	13	14					27
51	Bahamas			25							
51	Montenegro										
53	Belarus			105	78	89	70		39	36	94
54	Romania			28	27		46		32		62
55	Libya										
56	Oman			33	27	39			34		66
57	Russian Federation			28	32	39		45			55
58	Bulgaria	27			43	42	38	50	41		55
59	Barbados			94	87		99	106	93		145
60	Palau			121							
61	Antigua and Barbuda								6		13
62	Malaysia	52	48	104		51	50	43	80		89
63	Mauritius		23	49	43	34	24	21	14		48
64	Trinidad and Tobago	29	45	49							
65	Lebanon			11	12	11	8	3	3	3	4
65	Panama	33	26	76				34		26	55
67	Venezuela (Bolivarian Republic of)	30				32	32				46
68	Costa Rica	64		64		54	54	65	88		131
69	Turkey		6	21		14					27
70	Kazakhstan			39	7	8	14	11	12		31
71	Mexico			72	61	57	60	61	61		99
71	Seychelles		55			57					82

Table I. Country rank per year for Expenditure on education, Public (% of GDP)

HDI Rank	Countries	1980	1985	2000	2005	2006	2007	2008	2009	2011	2012
73	Saint Kitts and Nevis		43	71	35	44	44				62
73	Sri Lanka	17	11						4	5	7
75	Iran (Islamic Republic of)		22	64	52	64	79	57	46		75
76	Azerbaijan			54	20	8	6	6	14		23
77	Jordan										
77	Serbia						54	61	50		75
79	Brazil			57	50	62	68	76	68		116
79	Georgia			15	11	17	13	15	14	8	20
79	Grenada										
82	Peru				13	4	6	12	10	6	17
83	Ukraine			60	81	91	73				99
84	Belize			76				89	83		134
84	The former Yugoslav Republic of Macedonia										
86	Bosnia and Herzegovina										
87	Armenia			25	13	11	16	21	26	13	31
88	Fiji			101	62	80	86	47	39	26	55
89	Thailand	14		89	43	47	36	34	28	40	116
90	Tunisia	44	42	105	86	95	95	100	90		128
91	China	6	8								
91	Saint Vincent and the Grenadines			115	83		85		72		89
93	Algeria	60						48			66
93	Dominica										44
95	Albania			35	23	23	23				35
96	Jamaica		37	76	51		79	99	86		132
97	Saint Lucia			109	66	85		74	26	29	69
98	Colombia	3	14	43	36	39	42	40	46	30	70
98	Ecuador		52	3						36	94
100	Suriname										
100	Tonga			72							
102	Dominican Republic		2	11			5				10
103	Maldives				78	80	77	76	98	42	141
103	Mongolia			94			54		54	39	107
103	Turkmenistan										
106	Samoa			57				92			116
107	Palestine, State of										
108	Indonesia				19	30	16	15	24	10	23
109	Botswana	50	44		100		103		98		148
110	Egypt	32			55	42	34	34			50
111	Paraguay		4	86			39				55
112	Gabon			49							
113	Bolivia (Plurinational State of)			91		94		109	103		146

Table I. Country rank per year for Expenditure on education, Public (% of GDP)

HDI Rank	Countries	1980	1985	2000	2005	2006	2007	2008	2009	2011	2012
114	Moldova (Republic of)			67	92	101	104	115	105	45	154
115	El Salvador			19	13	17	20	32		18	41
116	Uzbekistan										
117	Philippines	3		39	9	4	8	12	7		20
118	South Africa			94	66	68	70	67	67		123
118	Syrian Arab Republic	39	49			68	61	51	54		89
120	Iraq	11									
121	Guyana			118	97	64	36		23	21	46
121	Viet Nam							74			134
123	Cabo Verde					96	92	100	72		109
124	Micronesia (Federated States of)			109							
125	Guatemala					17	16	21			23
125	Kyrgyzstan			43	56	75	95	95	86		116
127	Namibia			115		86		102			152
128	Timor-Leste							116	106	46	156
129	Honduras	21									
129	Morocco	55	46	100		75		84	63		102
131	Vanuatu			112				105	60		94
132	Nicaragua	23		31							75
133	Kiribati			122							
133	Tajikistan			16	27	27	24	29	28	24	53
135	India			62	21	23			14		35
136	Bhutan			91	91			57	41	33	75
136	Cambodia			9			3				17
138	Ghana	17			94	68	79	92	61	44	151
139	Lao People's Democratic Republic			6	9	17	20	5			35
140	Congo	58			2						128
141	Zambia	31	24	14	4		2	1			3
142	Bangladesh	1	2	17		4	8	6	5		10
142	Sao Tome and Principe										
144	Equatorial Guinea			2							
145	Nepal			31	25	30	28	34	46		75
146	Pakistan	8	9	10	7	8	14	15	7		12
147	Kenya	48	36	83	93	99					137
148	Swaziland	46		91	94	104		111	95	43	148
149	Angola		28	21	17	14					44
150	Myanmar			1						1	1
151	Rwanda	17		59			46	40		35	82
152	Cameroon			11	21	17	24	15	25	15	34
152	Nigeria										
154	Yemen			119				71			94

Table I. Country rank per year for Expenditure on education, Public (% of GDP)

HDI Rank	Countries	1980	1985	2000	2005	2006	2007	2008	2009	2011	2012
155	Madagascar	33	15	28	32	25	24	15	14	10	23
156	Zimbabwe	13	51								13
157	Papua New Guinea										
157	Solomon Islands						87	97	102		143
159	Comoros							112			146
159	Tanzania (United Republic of)							107			128
161	Mauritania					4		43		22	48
162	Lesotho		53	123	102	107		117			158
163	Senegal			35	62	57		65	68		109
164	Uganda		18	19					21	15	35
165	Benin			33	36	34	28	45	41		99
166	Sudan										
166	Togo			67	25	32	34	27	28	32	73
168	Haiti										
169	Afghanistan	5									
170	Djibouti			119	99	105	105				152
171	Côte d'Ivoire	62		49	47	44	50	51			73
172	Gambia		26	6				29	12	24	53
173	Ethiopia		9	54		75	79	76	41		75
	**Total ....**	**66**	**55**	**124**	**102**	**107**	**106**	**118**	**107**	**46**	**158**
	**Normalized rank**	0.500	0.273	0.226	0.314	0.234	0.226	0.127	0.131	0.217	0.146

International Human Development Indicators
Accessed: 7/14/2014,2:10 PM from: http://hdr.undp.org

Female to male ratio, parliamentary seats
Percentage of parliamentary seats held by women expressed as a ratio of those held by men.
Source: Calculated based on data in columns 3 and 4.
Data in the tables are those available to the Human Development Report Office as of 15 November, 2013, unless otherwise specified. (Values for Ecuador in 2011 corrected, and Vanuatu in 2000 deleted)

Table J. Percentage of parliamentary seats held by women expressed as a ratio of those held by men

HDI Rank	Countries	2000	2005	2008	2009	2010	2011	2012	2013
1	Norway	0.572	0.61	0.565	0.656	0.656	0.656	0.656	0.657
2	Australia	0.333	0.395	0.42	0.431	0.395	0.395	0.412	0.412
3	Switzerland	0.289	0.33	0.374	0.381	0.381	0.366	0.366	0.374
4	Netherlands	0.49	0.52	0.642	0.656	0.608	0.618	0.642	0.607
5	United States		0.176	0.205	0.198	0.202	0.203	0.203	0.222
6	Germany	0.437	0.439	0.451	0.464	0.464	0.464	0.479	0.479
7	New Zealand	0.445	0.475	0.506	0.506	0.506	0.475	0.475	0.476
8	Canada		0.328	0.332	0.332	0.332	0.379	0.383	0.389
9	Singapore		0.19	0.325	0.305	0.305	0.185	0.285	0.32
10	Denmark	0.597	0.585	0.613	0.613	0.613	0.642	0.642	0.642
11	Ireland	0.159	0.166	0.183	0.192	0.203	0.235	0.235	0.242
12	Sweden	0.745	0.828	0.887	0.866	0.818	0.818	0.808	0.808
13	Iceland	0.536	0.499	0.499	0.751	0.751	0.751	0.658	0.658
14	United Kingdom	0.205	0.227	0.244	0.247	0.266	0.266	0.284	0.291
15	Korea (Republic of)		0.155	0.159	0.172	0.172	0.172	0.172	0.186
17	Japan	0.12	0.12	0.14	0.153	0.157	0.157	0.155	0.121
18	Liechtenstein		0.316	0.316	0.316	0.316	0.316	0.316	0.25
19	Israel	0.143	0.176	0.166	0.224	0.238	0.238	0.25	0.29
20	France	0.1	0.161	0.244	0.25	0.25	0.252	0.252	0.335
21	Luxembourg	0.2	0.304	0.304	0.25	0.25	0.25	0.333	0.277
21	Austria	0.335	0.475	0.362	0.395	0.395	0.395	0.403	0.402
21	Belgium	0.332	0.555	0.567	0.637	0.626	0.626	0.637	0.637
24	Finland	0.575	0.6	0.709	0.667	0.667	0.739	0.739	0.739
25	Slovenia		0.121	0.111	0.121	0.121	0.121	0.3	0.327
26	Italy	0.111	0.116	0.253	0.255	0.255	0.255	0.259	0.441
27	Spain	0.35	0.439	0.506	0.517	0.531	0.536	0.536	0.544
28	Czech Republic	0.12	0.186	0.19	0.19	0.266	0.266	0.266	0.26
29	Greece		0.149	0.172	0.209	0.209	0.209	0.23	0.266
31	Qatar			0.001	0.001	0.001	0.001	0.001	0.001
32	Cyprus		0.192	0.167	0.143	0.143	0.12	0.12	0.12
33	Estonia	0.217	0.232	0.263	0.295		0.247	0.247	0.263
34	Saudi Arabia		0.001	0.001	0.001	0.001	0.001	0.001	0.248
35	Poland	0.145	0.236	0.22	0.218	0.218	0.282	0.279	0.279
35	Lithuania		0.209	0.215	0.237	0.236	0.236	0.236	0.318

Table J. Percentage of parliamentary seats held by women expressed as a ratio of those held by men

HDI Rank	Countries	2000	2005	2008	2009	2010	2011	2012	2013
37	Slovakia	0.163	0.2	0.239	0.22	0.19	0.19	0.19	0.23
37	Andorra		0.401	0.333	0.555	0.555	1	1	1
39	Malta		0.101	0.095	0.095	0.095	0.095	0.095	0.167
40	United Arab Emirates	0.001	0.001	0.29	0.29	0.29	0.212	0.212	0.212
41	Chile	0.098	0.135	0.145	0.161	0.161	0.161	0.161	0.162
41	Portugal	0.211	0.271	0.395	0.377	0.377	0.361	0.403	0.402
43	Hungary		0.1	0.125	0.125	0.1	0.1	0.096	0.097
44	Bahrain		0.081	0.16	0.16	0.176	0.231	0.231	0.231
44	Cuba	0.381	0.563	0.761	0.761	0.761	0.761	0.825	0.955
46	Kuwait	0.001	0.015	0.032	0.083	0.083	0.083	0.083	0.066
47	Croatia	0.192	0.225	0.264	0.307	0.307	0.307	0.312	0.313
48	Latvia	0.205	0.266	0.25	0.282	0.25	0.266	0.299	0.299
49	Argentina	0.271	0.597	0.661	0.608	0.608	0.605	0.605	0.605
50	Uruguay	0.13	0.121	0.14	0.163	0.171	0.171	0.14	0.14
51	Bahamas	0.244	0.366	0.333	0.218	0.218	0.218	0.218	0.2
51	Montenegro			0.125	0.125	0.125	0.125	0.14	0.209
53	Belarus	0.117	0.425	0.481	0.473	0.473	0.473	0.473	0.419
54	Romania		0.12	0.109	0.109	0.109	0.109	0.107	0.131
55	Libya		0.049	0.083	0.083	0.083	0.083	0.083	0.198
56	Oman		0.085	0.1	0.099	0.099	0.106	0.106	0.106
57	Russian Federation	0.06	0.087	0.13	0.13	0.13	0.13	0.125	0.137
58	Bulgaria		0.42	0.277	0.263	0.263	0.263	0.263	0.326
59	Barbados	0.256	0.214	0.159	0.244	0.244	0.244	0.244	0.275
60	Palau	0.034	0.001	0.074	0.074	0.074	0.074	0.074	0.115
61	Antigua and Barbuda	0.091	0.161	0.2	0.241	0.241	0.241	0.241	0.241
62	Malaysia	0.17	0.151	0.171	0.163	0.163	0.163	0.148	0.162
63	Mauritius		0.206	0.206	0.206	0.232	0.232	0.232	0.232
64	Trinidad and Tobago		0.34	0.499	0.499	0.377	0.377	0.377	0.352
65	Lebanon	0.024	0.049	0.049	0.032	0.032	0.032	0.032	0.032
65	Panama		0.2	0.2	0.093	0.093	0.093	0.093	0.092
67	Venezuela (Bolivarian Republic of)		0.211	0.229	0.212	0.205	0.205	0.205	0.204
68	Costa Rica	0.239	0.541	0.582	0.582	0.629	0.629	0.629	0.629
69	Turkey		0.046	0.1	0.1	0.1	0.166	0.166	0.165
70	Kazakhstan	0.126	0.094	0.14	0.157	0.157	0.14	0.157	0.222
71	Mexico	0.189	0.311	0.284	0.351	0.342	0.342	0.342	0.562
71	Seychelles	0.307	0.416	0.307	0.307	0.307	0.778	0.778	0.778
73	Sri Lanka		0.052	0.062	0.062	0.056	0.062	0.062	0.061
73	Saint Kitts and Nevis	0.153	0.001	0.072	0.072	0.072	0.072	0.072	0.071
75	Iran (Islamic Republic of)	0.035	0.043	0.029	0.029	0.029	0.029	0.029	0.032
76	Azerbaijan	0.001	0.149	0.129	0.129	0.19	0.19	0.19	0.19
77	Jordan	0.026	0.079	0.093	0.093	0.139	0.125	0.125	0.137

Table J. Percentage of parliamentary seats held by women expressed as a ratio of those held by men

HDI Rank	Countries	2000	2005	2008	2009	2010	2011	2012	2013
77	Serbia			0.276	0.276	0.276	0.276	0.808	0.497
79	Brazil	0.063	0.1	0.104	0.103	0.106	0.106	0.106	0.106
79	Georgia		0.104	0.064	0.054	0.07	0.07	0.071	0.136
79	Grenada	0.218	0.473	0.272	0.272	0.272	0.272	0.218	0.333
82	Peru		0.224	0.412	0.379	0.379	0.274		0.275
83	Ukraine		0.056	0.089	0.087	0.087	0.087	0.087	0.104
84	Belize	0.156	0.135	0.125	0.125	0.125	0.125	0.125	0.154
84	The former Yugoslav Republic of Macedonia		0.238	0.464	0.481	0.481	0.447	0.447	0.519
86	Bosnia and Herzegovina		0.14	0.14	0.212	0.188	0.188	0.239	0.239
87	Armenia	0.032	0.056	0.092	0.101	0.101	0.101	0.092	0.12
88	Fiji		0.107						
89	Thailand	0.073	0.119	0.145	0.163	0.163	0.186	0.186	0.186
90	Tunisia		0.239	0.248	0.304		0.357	0.364	0.365
91	Saint Vincent and the Grenadines		0.222	0.222	0.277	0.167	0.211	0.211	0.15
91	China	0.001	0.255	0.271	0.271	0.271	0.271	0.271	0.306
93	Dominica		0.148	0.231	0.167	0.143	0.143	0.143	0.143
93	Algeria	0.042	0.056	0.07	0.076	0.075	0.075	0.078	0.348
95	Albania		0.076	0.076	0.196	0.196	0.196	0.186	0.217
96	Jamaica	0.19	0.157	0.157	0.157	0.19	0.19	0.183	0.183
97	Saint Lucia	0.16	0.261	0.208	0.261	0.261	0.261	0.318	0.208
98	Colombia	0.139	0.122	0.107	0.107	0.16	0.16	0.157	0.157
98	Ecuador	0.171	0.19	0.333	0.477	0.477	0.477	0.477	0.631
100	Tonga	0.001	0.035	0.032	0.032	0.037	0.037	0.037	0.037
100	Suriname		0.342	0.342	0.342	0.109	0.109	0.134	0.133
102	Dominican Republic	0.17	0.182	0.206	0.206	0.236	0.236	0.236	0.236
103	Maldives		0.136	0.136	0.07	0.07	0.07	0.07	0.069
103	Mongolia		0.072	0.044	0.042	0.041	0.041	0.041	0.175
103	Turkmenistan	0.351	0.19	0.001	0.202	0.202	0.202	0.202	0.202
106	Samoa		0.065	0.089	0.089	0.043	0.043	0.043	0.043
108	Indonesia		0.127	0.131	0.218	0.22	0.22	0.222	0.228
109	Botswana	0.205	0.125	0.125	0.086	0.086	0.086	0.086	0.086
110	Egypt	0.02	0.04	0.038	0.037			0.013	0.029
111	Paraguay	0.087	0.106	0.157	0.157	0.157	0.157	0.157	0.225
112	Gabon		0.135	0.192	0.192	0.192	0.192	0.188	0.2
113	Bolivia (Plurinational State of)	0.114	0.171	0.171	0.361	0.431	0.431	0.431	0.431
114	Moldova (Republic of)		0.14	0.279	0.312	0.232	0.232	0.247	0.247
115	El Salvador		0.12	0.2	0.236	0.235	0.235	0.235	0.355
116	Uzbekistan		0.196	0.196	0.238	0.238	0.238	0.238	0.238
117	Philippines	0.134	0.182	0.253	0.261	0.274	0.274	0.284	0.368
118	Syrian Arab Republic		0.136	0.142	0.142	0.142	0.142	0.142	0.136
118	South Africa	0.431	0.488	0.513	0.745	0.745	0.745	0.698	0.697

Table J. Percentage of parliamentary seats held by women expressed as a ratio of those held by men

HDI Rank	Countries	2000	2005	2008	2009	2010	2011	2012	2013
120	Iraq		0.001	0.342	0.342	0.337	0.337	0.337	0.337
121	Viet Nam	0.351	0.376	0.348	0.348	0.3	0.323	0.323	0.323
121	Guyana	0.227	0.445	0.429	0.429	0.429	0.429	0.456	0.457
123	Cabo Verde		0.125	0.221	0.221	0.263	0.263	0.263	0.263
124	Micronesia (Federated States of)		0.001	0.001	0.001	0.001	0	0.001	0.001
125	Guatemala		0.089	0.136	0.136	0.136	0.136	0.153	0.153
125	Kyrgyzstan	0.064	0.001	0.344	0.344	0.304	0.304	0.304	0.304
127	Namibia	0.256	0.368	0.368	0.368	0.333	0.333	0.333	0.333
128	Timor-Leste		0.339	0.412	0.412	0.412	0.412	0.477	0.625
129	Morocco	0.007	0.068	0.066	0.072	0.072	0.121	0.124	0.123
129	Honduras		0.305	0.305	0.22	0.22	0.22	0.242	0.243
131	Vanuatu	1	0.04	0.04	0.041	0.04	0.019	0.019	0.001
132	Nicaragua		0.261	0.227	0.261	0.261	0.672	0.672	0.673
133	Kiribati		0.05	0.045	0.046	0.045	0.095	0.095	0.095
133	Tajikistan	0.163	0.244	0.244	0.244	0.212	0.212	0.212	0.213
135	India		0.103	0.101	0.115	0.12	0.12	0.122	0.122
136	Bhutan		0.103	0.161	0.161	0.161	0.161	0.161	0.075
136	Cambodia	0.073	0.122	0.188	0.235	0.235	0.235	0.227	0.221
138	Ghana		0.122	0.086	0.091	0.091	0.091	0.091	0.122
139	Lao People's Democratic Republic	0.269	0.297	0.337	0.337	0.337	0.333	0.333	0.333
140	Congo	0.136	0.112	0.101	0.101	0.101	0.106	0.106	0.106
141	Zambia		0.145	0.179	0.163	0.163	0.124	0.13	0.129
142	Sao Tome and Principe		0.1	0.079	0.079	0.222	0.222	0.222	0.222
142	Bangladesh		0.174	0.067	0.229	0.229	0.229	0.245	0.246
144	Equatorial Guinea		0.22	0.064	0.111	0.111	0.111	0.111	0.232
145	Nepal	0.086	0.068	0.497	0.497	0.497	0.497	0.497	0.496
146	Pakistan		0.259	0.269	0.266	0.266	0.266	0.271	0.245
147	Kenya	0.037	0.076	0.109	0.109	0.109	0.109	0.109	0.248
148	Swaziland	0.067	0.202	0.284	0.28	0.28	0.28	0.28	0.28
149	Angola		0.176	0.595	0.629	0.629	0.629	0.618	0.517
150	Myanmar					0.042	0.042	0.03	0.048
151	Rwanda	0.346	0.828	1.037	1.037	1.037	1.079	1.079	1.078
152	Nigeria	0.034	0.062	0.079	0.079	0.079	0.038	0.072	0.071
152	Cameroon		0.098	0.161	0.161	0.161	0.161	0.161	0.191
154	Yemen	0.007	0.007	0.007	0.007	0.007	0.007	0.007	0.007
155	Madagascar		0.092	0.134		0.138	0.138	0.138	0.188
156	Zimbabwe		0.263	0.222	0.218	0.218	0.218	0.218	0.542
157	Solomon Islands	0.02	0.001	0.001	0.001	0.001	0.001	0.001	0.02
157	Papua New Guinea	0.018	0.009	0.009	0.009	0.009	0.009	0.009	0.028
159	Comoros		0.031	0.031	0	0.031	0.031	0.031	0.031
159	Tanzania (United Republic of)		0.437	0.437	0.443	0.563	0.563	0.563	0.563

Table J. Percentage of parliamentary seats held by women expressed as a ratio of those held by men

HDI Rank	Countries	2000	2005	2008	2009	2010	2011	2012	2013
161	Mauritania	0.031		0.248	0.238	0.238	0.238	0.238	0.238
162	Lesotho	0.12	0.205	0.248	0.297	0.297	0.297	0.307	0.366
163	Senegal	0.163	0.238	0.412	0.42	0.42	0.42	0.42	0.744
164	Uganda	0.217	0.314	0.443	0.46		0.536	0.538	0.538
165	Benin		0.078	0.121	0.121	0.121	0.092	0.092	0.092
166	Togo		0.08	0.125	0.125	0.125	0.125	0.125	0.182
166	Sudan		0.157	0.202	0.214	0.319	0.326	0.318	0.317
168	Haiti		0.1	0.055	0.049	0.044	0.042	0.042	0.036
169	Afghanistan		0.35	0.35	0.35	0.381	0.381	0.381	0.382
170	Djibouti	0.001	0.121	0.16	0.161	0.16	0.16		0.146
171	Côte d'Ivoire		0.093	0.098	0.098	0.098	0.098	0.124	0.117
172	Gambia	0.02	0.152	0.104	0.082	0.081	0.081	0.081	0.082
173	Ethiopia		0.266	0.272	0.272	0.342	0.342	0.342	0.343
174	Malawi		0.157	0.149	0.263	0.263	0.263	0.287	0.287
175	Liberia	0.125	0.16	0.16	0.16	0.16	0.16	0.12	0.132
176	Mali	0.139	0.114	0.114	0.114	0.114	0.114	0.114	0.114
177	Guinea-Bissau		0.163	0.111	0.111	0.111	0.111	0.111	0.163
178	Mozambique	0.429	0.534	0.534	0.645	0.645	0.645	0.645	0.645
179	Guinea		0.239						

Table K. Country rank per year for percentage of parliamentary seats held by women expressed as a ratio of those held by men

HDI Rank	Countries	2005	2008	2010	2011	2012	2013
1	Norway	169	163	164	163	162	164
2	Australia	147	149	143	143	141	140
3	Switzerland	139	144	141	138	135	135
4	Netherlands	161	168	157	157	159	157
5	United States	94	93	82	81	78	80
6	Germany	153	153	148	149	150	146
7	New Zealand	158	160	153	151	147	145
8	Canada	138	132	131	140	138	137
9	Singapore	100	131	126	72	117	119
10	Denmark	166	167	159	161	160	162
11	Ireland	90	84	83	96	92	93
12	Sweden	171	172	169	170	169	171
13	Iceland	160	158	167	167	163	165
14	United Kingdom	116	107	115	116	115	112
15	Korea (Republic of)	82	71	73	71	70	63
17	Japan	57	62	60	60	62	36
18	Liechtenstein	137	130	129	128	124	100

Table K. Country rank per year for percentage of parliamentary seats held by women expressed as a ratio of those held by men

HDI Rank	Countries	2005	2008	2010	2011	2012	2013
19	Israel	95	78	100	101	105	111
20	France	88	106	105	108	106	126
21	Luxembourg	133	127	107	107	128	107
21	Austria	157	142	142	142	140	139
21	Belgium	164	164	160	158	158	161
24	Finland	168	170	165	165	166	168
25	Slovenia	62	46	47	48	120	122
26	Italy	55	113	108	109	107	143
27	Spain	154	159	154	153	152	153
28	Czech Republic	98	86	114	115	110	101
29	Greece	79	82	85	83	89	104
31	Qatar		5	4	4	4	2
32	Cyprus	102	79	57	46	44	35
33	Estonia	117	115		106	104	102
34	Saudi Arabia	6	3	3	3	3	98
35	Poland	118	98	87	124	113	108
35	Lithuania	109	97	99	100	95	118
37	Slovakia	105	105	78	77	76	84
37	Andorra	148	135	155	171	171	173
39	Malta	47	35	32	32	34	59
40	United Arab Emirates	5	126	122	86	82	75
41	Chile	71	67	65	65	66	56
41	Portugal	131	145	137	137	139	138
43	Hungary	45	51	36	34	35	26
44	Bahrain	35	73	74	93	90	85
44	Cuba	165	171	168	168	170	172
46	Kuwait	11	10	26	24	25	16
47	Croatia	115	116	127	127	123	116
48	Latvia	130	112	106	114	119	113
49	Argentina	167	169	158	156	155	156
50	Uruguay	61	63	72	70	57	48
51	Bahamas	144	134	89	88	84	70
51	Montenegro		54	51	52	56	74
53	Belarus	151	155	149	150	146	141
54	Romania	58	45	41	40	39	41
55	Libya	19	29	27	25	26	68
56	Oman	36	38	34	36	36	28
57	Russian Federation	37	56	52	54	52	47
58	Bulgaria	150	122	113	113	109	121
59	Barbados	111	72	104	105	101	105
60	Palau	4	25	22	21	22	32

Table K. Country rank per year for percentage of parliamentary seats held by women expressed as a ratio of those held by men

HDI Rank	Countries	2005	2008	2010	2011	2012	2013
61	Antigua and Barbuda	87	90	103	104	99	92
62	Malaysia	80	80	69	68	60	55
63	Mauritius	108	95	94	94	91	87
64	Trinidad and Tobago	141	157	138	139	136	130
65	Lebanon	18	16	9	10	12	10
65	Panama	104	91	31	30	32	24
67	Venezuela (Bolivarian Republic of)	110	103	84	82	79	72
68	Costa Rica	163	165	161	159	157	159
69	Turkey	17	37	35	69	69	58
70	Kazakhstan	41	61	59	57	63	79
71	Mexico	135	125	136	135	133	154
71	Seychelles	149	129	128	169	167	170
73	Sri Lanka	21	18	17	17	17	15
73	Saint Kitts and Nevis	3	24	21	20	21	19
75	Iran (Islamic Republic of)	16	8	7	8	9	9
76	Azerbaijan	78	55	76	75	75	66
77	Jordan	33	34	55	53	51	46
77	Serbia		121	120	122	168	148
79	Brazil	46	41	39	38	38	30
79	Georgia	50	19	18	18	19	44
79	Grenada	156	120	118	119	85	123
82	Peru	114	146	139	121		106
83	Ukraine	23	31	29	27	28	27
84	Belize	70	53	50	51	50	53
84	The former Yugoslav Republic of Macedonia	120	154	151	148	144	150
86	Bosnia and Herzegovina	74	64	75	74	98	91
87	Armenia	24	33	37	35	31	34
88	Fiji	52					
89	Thailand	56	66	68	73	72	64
90	Tunisia	121	110		136	134	132
91	Saint Vincent and the Grenadines	113	100	71	84	80	51
91	China	124	118	117	118	112	115
93	Dominica	77	104	58	59	59	49
93	Algeria	22	23	23	22	23	129
95	Albania	31	26	80	79	73	77
96	Jamaica	85	70	77	76	71	62
97	Saint Lucia	126	96	109	110	125	73
98	Colombia	64	43	62	62	65	54
98	Ecuador	101	133	150	173	149	160
100	Tonga	13	11	10	11	13	12
100	Suriname	142	138	42	41	54	43

Table K. Country rank per year for percentage of parliamentary seats held by women expressed as a ratio of those held by men

HDI Rank	Countries	2005	2008	2010	2011	2012	2013
102	Dominican Republic	96	94	98	99	94	88
103	Maldives	72	60	19	19	18	17
103	Mongolia	29	14	12	13	14	60
103	Turkmenistan	99	4	81	80	77	71
106	Samoa	26	32	14	16	16	13
108	Indonesia	68	57	90	89	86	83
109	Botswana	66	52	28	26	27	22
110	Egypt	14	12			7	7
111	Paraguay	51	69	61	61	64	82
112	Gabon	69	87	79	78	74	69
113	Bolivia (Plurinational State of)	91	81	147	147	143	142
114	Moldova (Republic of)	75	123	95	95	103	97
115	El Salvador	59	89	97	98	93	131
116	Uzbekistan	103	88	101	102	96	89
117	Philippines	97	114	119	120	116	134
118	Syrian Arab Republic	73	65	56	58	58	45
118	South Africa	159	161	166	166	165	167
120	Iraq	8	137	134	133	131	127
121	Viet Nam	146	140	124	129	127	120
121	Guyana	155	150	146	146	145	144
123	Cabo Verde	67	99	112	112	108	103
124	Micronesia (Federated States of)	1	1	1	1	1	1
125	Guatemala	38	59	53	55	61	52
125	Kyrgyzstan	7	139	125	126	121	114
127	Namibia	145	143	132	131	129	124
128	Timor-Leste	140	148	144	144	148	158
129	Morocco	27	21	20	47	48	39
129	Honduras	134	128	91	90	100	94
131	Vanuatu	15	13	11	7	8	3
132	Nicaragua	127	102	110	164	164	166
133	Kiribati	20	15	16	31	33	25
133	Tajikistan	123	108	86	85	81	76
135	India	49	39	46	45	46	38
136	Bhutan	48	77	67	67	68	20
136	Cambodia	65	85	96	97	88	78
138	Ghana	63	30	30	28	29	37
139	Lao People's Democratic Republic	132	136	133	132	130	125
140	Congo	53	40	38	37	37	29
141	Zambia	76	83	70	49	53	40
142	Sao Tome and Principe	43	28	92	91	87	81
142	Bangladesh	92	22	93	92	102	96

Table K. Country rank per year for percentage of parliamentary seats held by women expressed as a ratio of those held by men

HDI Rank	Countries	2005	2008	2010	2011	2012	2013
144	Equatorial Guinea	112	20	43	42	41	86
145	Nepal	28	156	152	152	151	147
146	Pakistan	125	117	116	117	111	95
147	Kenya	30	44	40	39	40	99
148	Swaziland	106	124	121	123	114	109
149	Angola	93	166	162	160	156	149
150	Myanmar			13	14	10	14
151	Rwanda	170	173	170	172	172	174
152	Nigeria	25	27	24	12	20	18
152	Cameroon	42	76	66	66	67	67
154	Yemen	9	6	5	5	5	4
155	Madagascar	39	58	54	56	55	65
156	Zimbabwe	128	101	88	87	83	152
157	Solomon Islands	2	2	2	2	2	5
157	Papua New Guinea	10	7	6	6	6	6
159	Comoros	12	9	8	9	11	8
159	Tanzania (United Republic of)	152	151	156	155	154	155
161	Mauritania		111	102	103	97	90
162	Lesotho	107	109	123	125	122	133
163	Senegal	119	147	145	145	142	169
164	Uganda	136	152		154	153	151
165	Benin	32	49	48	29	30	23
166	Togo	34	50	49	50	49	61
166	Sudan	84	92	130	130	126	117
168	Haiti	44	17	15	15	15	11
169	Afghanistan	143	141	140	141	137	136
170	Djibouti	60	74	63	63		50
171	Côte d'Ivoire	40	36	33	33	47	33
172	Gambia	81	42	25	23	24	21
173	Ethiopia	129	119	135	134	132	128
174	Malawi	83	68	111	111	118	110
175	Liberia	86	75	64	64	45	42
176	Mali	54	48	45	44	43	31
177	Guinea-Bissau	89	47	44	43	42	57
178	Mozambique	162	162	163	162	161	163
179	Guinea	122					
	**Total ....**	**171**	**173**	**170**	**173**	**172**	**174**
	**Normalized rank**	0.228	0.335	0.318	0.324	0.320	0.374

International Human Development Indicators
Accessed: 7/14/2014,2:07 PM from: http://hdr.undp.org

Under-five mortality rate (per 1,000 live births)
Probability of dying between birth and exactly age 5, expressed per 1,000 live births.
Source: Inter-agency Group for Child Mortality Estimation (2013). Accessed September, 2013.
Data in the tables are those available to the Human Development Report Office as of 15 November, 2013, unless otherwise specified.

Table L. Probability of dying between birth and exactly age 5, expressed per 1,000 live births

HDI Rank	Countries	1980	1985	1990	2000	2005	2010	2011	2012	2013
1	Norway	10	10	9	5	4	3	3	3	3
2	Australia	13	11	9	6	6	5	5	5	5
3	Switzerland	10	9	8	6	5	5	4	4	4
4	Netherlands	11	10	8	6	5	4	4	4	4
5	United States	15	13	11	8	8	7	7	7	7
6	Germany	15	11	9	5	5	4	4	4	4
7	New Zealand	16	14	11	7	7	6	6	6	6
8	Canada	13	10	8	6	6	6	5	5	5
9	Singapore	15	11	8	4	3	3	3	3	3
10	Denmark	10	10	9	6	5	4	4	4	4
11	Ireland	14	11	9	7	5	4	4	4	4
12	Sweden	8	8	7	4	4	3	3	3	3
13	Iceland	10	8	6	4	3	3	2	2	2
14	United Kingdom	14	11	9	7	6	5	5	5	5
15	Korea (Republic of)	14	10	7	6	6	4	4	4	4
17	Japan	10	8	6	5	4	3	3	3	3
19	Israel	18	14	12	7	6	5	4	4	4
20	France	12	10	9	5	5	4	4	4	4
21	Luxembourg	13	11	9	5	4	3	2	2	2
21	Belgium	15	12	10	6	5	4	4	4	4
21	Austria	16	13	10	6	5	4	4	4	4
24	Finland	9	7	7	4	4	3	3	3	3
25	Slovenia		14	10	6	4	3	3	3	3
26	Italy	16	12	10	6	4	4	4	4	4
27	Spain	18	13	11	7	6	5	5	5	5
28	Czech Republic		17	15	7	5	4	4	4	4
29	Greece	24	18	13	8	6	5	5	5	5
30	Brunei Darussalam		15	12	10	9	8	8	8	8
31	Qatar	36	28	21	12	10	8	8	7	7
32	Cyprus	21	13	11	7	5	4	3	3	3
33	Estonia	27	23	20	11	7	4	4	4	4
34	Saudi Arabia	98	70	47	22	15	10	9	9	9
35	Poland	24	20	17	9	8	6	5	5	5
35	Lithuania	23	19	17	12	10	7	6	5	5

Table L. Probability of dying between birth and exactly age 5, expressed per 1,000 live births

HDI Rank	Countries	1980	1985	1990	2000	2005	2010	2011	2012	2013
37	Andorra			8	5	4	4	3	3	3
37	Slovakia		22	18	12	10	8	8	8	8
39	Malta	17	14	11	8	7	7	7	7	7
40	United Arab Emirates	36	23	17	11	10	9	9	8	8
41	Portugal	28	20	15	7	5	4	4	4	4
41	Chile	33	22	19	11	9	9	9	9	9
43	Hungary	26	24	19	11	8	7	6	6	6
44	Cuba	20	18	13	8	7	6	6	6	6
44	Bahrain	32	25	23	13	11	10	10	10	10
46	Kuwait	36	23	16	13	12	11	11	11	11
47	Croatia		18	13	8	7	5	5	5	5
48	Latvia	25	21	20	17	13	9	9	9	9
49	Argentina	43	33	28	20	17	15	15	14	14
50	Uruguay	41	31	23	16	16	9	8	7	7
51	Montenegro		24	17	14	11	7	6	6	6
51	Bahamas	30	28	23	17	18	18	17	17	17
53	Belarus	23	19	17	14	9	6	6	5	5
54	Romania	43	38	38	27	21	14	13	12	12
55	Libya	74	56	43	28	23	17	16	15	15
56	Oman	110	66	39	17	13	12	12	12	12
57	Russian Federation	33	30	26	23	17	12	11	10	10
58	Bulgaria	29	24	22	21	16	13	13	12	12
59	Barbados	27	22	18	18	20	19	19	18	18
60	Palau		37	34	28	25	22	21	21	21
61	Antigua and Barbuda			24	16	13	11	10	10	10
62	Malaysia	31	22	17	10	8	8	9	9	9
63	Mauritius	40	28	23	19	16	15	15	15	15
64	Trinidad and Tobago	41	37	33	28	25	22	21	21	21
65	Lebanon	49	41	33	20	14	10	10	9	9
65	Panama	45	37	32	26	23	20	19	19	19
67	Venezuela (Bolivarian Republic of)	44	36	30	21	18	16	16	15	15
68	Costa Rica	24	22	17	13	10	10	10	10	10
69	Turkey	127	98	74	37	24	16	15	14	14
70	Kazakhstan	70	60	54	44	33	22	20	19	19
71	Seychelles	31	21	17	14	14	14	13	13	13
71	Mexico	74	59	46	25	20	17	17	16	16
73	Saint Kitts and Nevis	52	38	29	18	13	10	10	9	9
73	Sri Lanka	50	30	21	17	13	10	10	10	10
75	Iran (Islamic Republic of)	106	75	56	35	26	19	18	18	18
76	Azerbaijan		99	93	72	51	39	37	35	35
77	Serbia		39	28	13	9	8	7	7	7

Table L. Probability of dying between birth and exactly age 5, expressed per 1,000 live births

HDI Rank	Countries	1980	1985	1990	2000	2005	2010	2011	2012	2013
77	Jordan	55	44	37	28	24	20	20	19	19
79	Grenada		26	22	16	15	14	14	14	14
79	Brazil	94	75	62	33	23	16	15	14	14
79	Georgia			35	34	26	22	21	20	20
82	Peru	123	103	79	40	28	20	19	18	18
83	Ukraine	27	23	20	19	15	12	11	11	11
84	The former Yugoslav Republic of Macedonia		49	37	16	14	10	9	7	7
84	Belize	77	59	43	25	21	19	19	18	18
86	Bosnia and Herzegovina		27	18	10	9	8	7	7	7
87	Armenia	74	61	49	30	23	18	17	16	16
88	Fiji	44	36	31	24	22	23	23	22	22
89	Thailand	62	49	38	23	18	14	14	13	13
90	Tunisia	93	68	51	30	23	18	17	16	16
91	China	61	53	54	37	24	16	15	14	14
91	Saint Vincent and the Grenadines	57	35	25	22	23	24	24	23	23
93	Dominica	20	17	17	16	14	13	13	13	13
93	Algeria	155	75	50	35	26	22	21	20	20
95	Albania		50	43	29	22	18	17	17	17
96	Jamaica	40	34	30	23	21	18	17	17	17
97	Saint Lucia	33	27	22	18	18	18	18	18	18
98	Colombia	57	42	35	25	22	19	18	18	18
98	Ecuador	93	72	56	34	29	25	24	23	23
100	Tonga	30	26	23	18	16	14	13	13	13
100	Suriname		63	51	33	26	22	21	21	21
102	Dominican Republic	88	73	60	40	34	29	28	27	27
103	Maldives	155	120	94	45	24	13	12	11	11
103	Mongolia	150	129	107	63	43	30	29	28	28
103	Turkmenistan	123	103	90	79	67	56	55	53	53
106	Samoa		37	30	22	19	18	18	18	18
107	Palestine, State of	78	57	43	30	27	24	23	23	23
108	Indonesia	120	102	84	52	42	34	32	31	31
109	Botswana	72	56	48	85	67	58	56	53	53
110	Egypt	165	117	86	45	31	23	22	21	21
111	Paraguay	63	54	46	33	28	24	23	22	22
112	Gabon	116	100	92	86	79	67	65	62	62
113	Bolivia (Plurinational State of)	169	147	123	78	58	45	43	41	41
114	Moldova (Republic of)	48	39	32	30	23	19	18	18	18
115	El Salvador	110	80	59	32	23	17	17	16	16
116	Uzbekistan	111	85	74	61	51	43	41	40	40
117	Philippines	81	75	59	40	36	32	31	30	30
118	Syrian Arab Republic	63	48	38	24	19	15	15	15	15

Table L. Probability of dying between birth and exactly age 5, expressed per 1,000 live births

HDI Rank	Countries	1980	1985	1990	2000	2005	2010	2011	2012	2013
118	South Africa	91	73	61	74	79	53	47	45	45
120	Iraq	72	60	53	45	41	36	35	34	34
121	Viet Nam	67	60	51	32	26	23	23	23	23
121	Guyana	71	67	60	46	42	37	36	35	35
123	Cabo Verde	94	74	62	38	29	24	23	22	22
124	Micronesia (Federated States of)		58	55	54	48	41	40	39	39
125	Kyrgyzstan	103	84	71	50	40	31	28	27	27
125	Guatemala	124	101	80	51	41	34	33	32	32
127	Namibia	96	86	73	73	67	45	41	39	39
128	Timor-Leste		212	171	106	80	62	59	57	57
129	Honduras	95	75	59	38	31	25	24	23	23
129	Morocco	133	103	80	50	41	34	32	31	31
131	Vanuatu	67	47	35	24	22	19	19	18	18
132	Nicaragua	107	81	66	40	32	26	25	24	24
133	Tajikistan	129	119	105	91	74	63	60	58	58
133	Kiribati	119	112	94	71	66	63	61	60	60
135	India	166	145	126	92	75	61	59	56	56
136	Cambodia	185	120	116	111	63	44	42	40	40
136	Bhutan	195	161	131	80	61	48	46	45	45
138	Ghana	168	155	128	103	88	76	74	72	72
139	Lao People's Democratic Republic	199	182	163	120	98	78	75	72	72
140	Congo	122	104	100	118	113	102	99	96	96
141	Zambia	158	174	192	169	127	100	95	89	89
142	Bangladesh	198	173	144	88	68	47	44	41	41
142	Sao Tome and Principe	91	98	104	87	70	57	55	53	53
144	Equatorial Guinea		203	182	143	125	107	104	100	100
145	Nepal	209	177	142	82	61	46	44	42	42
146	Pakistan	160	150	138	112	101	90	88	86	86
147	Kenya	107	96	98	110	97	79	76	73	73
148	Swaziland	113	86	71	121	127	92	85	80	80
149	Angola	226	218	213	203	194	173	169	164	164
150	Myanmar	134	120	106	79	67	56	54	52	52
151	Rwanda	211	158	151	182	107	64	59	55	55
152	Cameroon	180	148	135	150	124	103	99	95	95
152	Nigeria	215	210	213	188	158	132	128	124	124
154	Yemen	206	153	125	97	78	64	62	60	60
155	Madagascar	171	179	159	109	81	63	61	58	58
156	Zimbabwe	102	79	74	102	97	97	95	90	90
157	Solomon Islands	52	43	39	35	36	33	32	31	31
157	Papua New Guinea	108	97	89	79	75	67	65	63	63
159	Tanzania (United Republic of)	176	176	166	132	90	62	57	54	54

Table L. Probability of dying between birth and exactly age 5, expressed per 1,000 live births

HDI Rank	Countries	1980	1985	1990	2000	2005	2010	2011	2012	2013
159	Comoros	173	146	124	99	94	83	80	78	78
161	Mauritania	160	142	128	111	102	89	87	84	84
162	Lesotho	120	95	85	114	123	108	102	100	100
163	Senegal	205	175	142	139	99	67	63	60	60
164	Uganda	212	187	178	147	109	78	74	69	69
165	Benin	214	200	181	147	120	96	93	90	90
166	Sudan	141	135	128	106	91	78	76	73	73
166	Togo	174	157	143	122	112	101	98	96	96
168	Haiti	190	166	144	105	91	175	78	76	76
169	Afghanistan	250	215	176	134	118	104	101	99	99
170	Djibouti	151	133	119	108	99	86	83	81	81
171	Côte d'Ivoire	166	153	152	145	131	114	111	108	108
172	Gambia	237	203	170	116	95	78	76	73	73
173	Ethiopia	240	221	204	146	110	76	72	68	68
174	Malawi	256	250	244	174	120	83	77	71	71
175	Liberia	239	235	248	176	119	83	78	75	75
176	Mali	321	283	253	220	173	138	133	128	128
177	Guinea-Bissau	224	215	206	174	156	137	133	129	129
178	Mozambique	254	247	233	166	132	101	96	90	90
179	Guinea	286	264	241	171	135	110	105	101	101
180	Burundi	221	177	164	150	134	112	108	104	104
181	Burkina Faso	240	218	202	186	160	115	108	102	102
182	Eritrea	189	174	150	89	70	56	54	52	52
183	Sierra Leone	285	269	257	234	216	193	187	182	182
184	Chad	234	222	209	189	176	159	154	150	150
185	Central African Republic	182	175	171	164	157	138	133	129	129
186	Congo (Democratic Republic of the)	204	179	171	171	171	155	150	146	146
187	Niger	315	330	326	227	174	127	120	114	114
	San Marino			11	6	4	4	3	3	3
	Monaco			8	5	5	4	4	4	4
	Korea (Democratic People's Rep. of)		35	44	60	33	31	30	29	29
	Tuvalu	70	62	58	42	37	32	31	30	30
	Nauru			58	42	39	39	38	37	37
	Marshall Islands	67	58	49	41	40	39	39	38	38
	South Sudan	294	276	251	181	140	112	108	104	104
	Somalia		188	177	171	171	156	152	147	147

Table M. Country rank per year for Probability of dying between birth and exactly age 5

HDI Rank	Countries	1980	1985	1990	2000	2005	2010	2011	2012	2013
1	Norway	5	6	12	8	7	6	7	7	7
2	Australia	11	13	16	17	26	26	27	27	27
3	Switzerland	7	5	6	13	15	25	25	25	25
4	Netherlands	8	8	7	14	16	15	16	16	16
5	United States	19	20	24	31	36	38	41	41	41
6	Germany	17	17	19	11	14	14	15	15	15
7	New Zealand	22	24	27	27	31	33	34	36	36
8	Canada	12	10	8	15	25	32	32	32	32
9	Singapore	16	16	9	4	2	2	3	3	3
10	Denmark	6	7	13	16	17	16	17	17	17
11	Ireland	13	14	17	23	20	19	20	20	20
12	Sweden	1	2	4	3	4	4	5	5	5
13	Iceland	3	3	1	1	1	1	1	1	1
14	United Kingdom	15	15	18	24	27	27	28	28	28
15	Korea (Republic of)	14	11	5	12	24	23	23	23	23
17	Japan	4	4	2	5	5	5	6	6	6
19	Israel	24	26	30	28	29	29	26	26	26
20	France	9	9	14	9	13	13	14	14	14
21	Luxembourg	10	12	15	10	8	7	2	2	2
21	Belgium	18	18	20	18	18	17	18	18	18
21	Austria	21	21	22	20	19	18	19	19	19
24	Finland	2	1	3	2	3	3	4	4	4
25	Slovenia		27	23	21	10	8	8	8	8
26	Italy	20	19	21	19	9	10	12	12	12
27	Spain	25	22	25	25	28	28	29	29	29
28	Czech Republic		30	35	29	22	21	21	21	21
29	Greece	31	32	33	34	30	30	30	30	30
30	Brunei Darussalam		28	31	37	40	43	44	47	47
31	Qatar	49	58	55	46	48	47	46	44	44
32	Cyprus	28	23	26	26	21	20	11	11	11
33	Estonia	36	45	53	43	35	24	24	24	24
34	Saudi Arabia	96	104	97	75	64	58	53	53	53
35	Poland	32	36	41	36	37	35	33	33	33
35	Lithuania	30	35	40	44	46	40	38	35	35
37	Andorra			10	6	6	9	9	9	9
37	Slovakia		44	48	45	47	46	45	48	48
39	Malta	23	25	28	32	32	37	40	40	40
40	United Arab Emirates	50	47	45	40	45	49	50	49	49
41	Portugal	39	37	36	30	23	22	22	22	22
41	Chile	46	43	50	41	42	48	49	51	51
43	Hungary	35	49	51	42	39	39	37	38	38

Table M. Country rank per year for Probability of dying between birth and exactly age 5

HDI Rank	Countries	1980	1985	1990	2000	2005	2010	2011	2012	2013
44	Cuba	26	31	32	33	33	34	35	37	37
44	Bahrain	45	52	60	49	50	53	55	57	57
46	Kuwait	51	48	37	47	52	59	60	61	61
47	Croatia		33	34	35	34	31	31	31	31
48	Latvia	34	38	52	59	54	50	51	52	52
49	Argentina	57	63	68	69	69	73	73	72	72
50	Uruguay	54	62	64	56	65	51	47	45	45
51	Montenegro		51	46	53	51	41	39	39	39
51	Bahamas	42	57	62	61	71	82	82	84	84
53	Belarus	29	34	39	51	44	36	36	34	34
54	Romania	56	73	85	86	81	71	69	66	66
55	Libya	81	89	91	90	88	80	79	79	79
56	Oman	103	101	89	62	56	61	63	64	64
57	Russian Federation	47	60	67	76	70	63	62	60	60
58	Bulgaria	40	50	57	71	68	65	66	65	65
59	Barbados	38	41	47	63	77	89	92	92	92
60	Palau		72	79	88	99	99	100	102	102
61	Antigua and Barbuda			65	57	53	60	59	59	59
62	Malaysia	44	42	44	38	38	42	48	50	50
63	Mauritius	52	59	63	68	67	72	72	76	76
64	Trinidad and Tobago	55	69	77	87	98	98	99	101	101
65	Lebanon	62	77	78	70	61	57	58	55	55
65	Panama	60	70	75	85	87	95	95	96	96
67	Venezuela (Bolivarian Republic of)	58	67	72	72	73	75	78	78	78
68	Costa Rica	33	40	43	48	49	52	54	56	56
69	Turkey	115	125	127	107	96	78	77	75	75
70	Kazakhstan	76	96	107	117	113	103	98	98	98
71	Seychelles	43	39	42	52	58	67	67	68	68
71	Mexico	82	93	96	84	78	79	80	80	80
73	Saint Kitts and Nevis	64	74	70	66	57	55	57	54	54
73	Sri Lanka	63	61	56	60	55	54	56	58	58
75	Iran (Islamic Republic of)	99	112	110	105	104	94	91	91	91
76	Azerbaijan		126	137	131	130	128	126	126	126
77	Serbia		76	69	50	43	45	43	43	43
77	Jordan	66	80	83	89	94	96	97	97	97
79	Grenada		54	58	55	62	68	70	71	71
79	Brazil	93	110	120	100	93	76	75	73	73
79	Georgia			82	101	102	101	102	99	99
82	Peru	112	130	128	113	107	97	96	95	95
83	Ukraine	37	46	54	67	63	62	61	62	62
84	The former Yugoslav Republic of Macedonia		84	84	58	60	56	52	46	46

Table M. Country rank per year for Probability of dying between birth and exactly age 5

HDI Rank	Countries	1980	1985	1990	2000	2005	2010	2011	2012	2013
84	Belize	84	94	93	83	80	90	93	93	93
86	Bosnia and Herzegovina		56	49	39	41	44	42	42	42
87	Armenia	83	98	100	94	90	87	85	82	82
88	Fiji	59	68	74	79	82	104	105	105	105
89	Thailand	70	83	87	78	74	70	71	70	70
90	Tunisia	90	103	104	95	91	88	86	83	83
91	China	69	86	106	106	95	77	76	74	74
91	Saint Vincent and the Grenadines	68	65	66	73	86	107	110	110	110
93	Dominica	27	29	38	54	59	64	65	67	67
93	Algeria	122	113	101	104	103	102	103	100	100
95	Albania		85	90	91	85	86	84	86	86
96	Jamaica	53	64	71	77	79	85	83	85	85
97	Saint Lucia	48	55	59	64	72	83	87	87	87
98	Colombia	67	78	80	82	84	92	89	89	89
98	Ecuador	91	105	109	102	108	111	111	111	111
100	Tonga	41	53	61	65	66	69	68	69	69
100	Suriname		100	103	99	101	100	101	103	103
102	Dominican Republic	87	106	117	111	115	114	114	114	114
103	Maldives	123	138	139	120	97	66	64	63	63
103	Mongolia	120	140	145	129	127	115	116	116	116
103	Turkmenistan	113	131	135	136	137	138	140	140	140
106	Samoa		71	73	74	75	84	88	88	88
107	Palestine, State of	85	90	92	93	105	108	107	109	109
108	Indonesia	110	129	131	125	126	123	122	122	122
109	Botswana	79	88	98	140	139	142	142	142	142
110	Egypt	127	135	133	119	111	106	104	104	104
111	Paraguay	72	87	95	98	106	109	108	106	106
112	Gabon	107	127	136	141	148	152	153	152	152
113	Bolivia (Plurinational State of)	131	146	148	134	131	132	133	133	133
114	Moldova (Republic of)	61	75	76	92	89	93	90	90	90
115	El Salvador	104	115	115	97	92	81	81	81	81
116	Uzbekistan	105	118	126	128	129	130	130	131	131
117	Philippines	86	109	113	110	117	118	118	118	118
118	Syrian Arab Republic	71	82	86	81	76	74	74	77	77
118	South Africa	88	107	118	133	147	137	137	137	137
120	Iraq	80	97	105	118	122	124	124	124	124
121	Viet Nam	74	95	102	96	100	105	106	108	108
121	Guyana	78	102	116	121	125	125	125	125	125
123	Cabo Verde	92	108	119	109	109	110	109	107	107
124	Micronesia (Federated States of)		92	108	126	128	129	129	129	129
125	Kyrgyzstan	98	117	122	122	121	117	115	115	115

Table M. Country rank per year for Probability of dying between birth and exactly age 5

HDI Rank	Countries	1980	1985	1990	2000	2005	2010	2011	2012	2013
125	Guatemala	114	128	129	124	124	122	123	123	123
127	Namibia	95	119	124	132	136	133	131	130	130
128	Timor-Leste		173	173	153	149	144	145	146	146
129	Honduras	94	111	114	108	110	112	112	112	112
129	Morocco	117	132	130	123	123	121	121	121	121
131	Vanuatu	73	81	81	80	83	91	94	94	94
132	Nicaragua	101	116	121	112	112	113	113	113	113
133	Tajikistan	116	136	143	145	143	147	147	147	147
133	Kiribati	108	134	138	130	135	146	148	149	149
135	India	129	144	151	146	145	143	144	145	145
136	Cambodia	138	139	146	157	134	131	132	132	132
136	Bhutan	141	154	155	138	132	136	136	136	136
138	Ghana	130	151	154	150	151	154	155	157	157
139	Lao People's Democratic Republic	143	166	167	163	159	158	157	158	158
140	Congo	111	133	141	162	168	173	174	175	175
141	Zambia	124	157	179	178	177	170	171	169	169
142	Bangladesh	142	156	162	143	140	135	135	134	134
142	Sao Tome and Principe	89	124	142	142	141	141	141	141	141
144	Equatorial Guinea		171	178	169	175	176	178	178	178
145	Nepal	147	162	159	139	133	134	134	135	135
146	Pakistan	126	148	157	159	162	166	168	168	168
147	Kenya	100	122	140	156	158	160	160	161	161
148	Swaziland	106	120	123	164	176	167	166	165	165
149	Angola	154	176	185	190	192	191	192	192	192
150	Myanmar	118	137	144	137	138	139	138	138	138
151	Rwanda	148	153	164	186	164	150	146	144	144
152	Cameroon	136	147	156	174	174	174	175	173	173
152	Nigeria	151	172	184	188	185	184	185	185	185
154	Yemen	146	150	150	147	146	149	150	150	150
155	Madagascar	132	164	166	155	150	148	149	148	148
156	Zimbabwe	97	114	125	149	157	169	170	171	171
157	Solomon Islands	65	79	88	103	116	120	120	120	120
157	Papua New Guinea	102	123	134	135	144	151	152	153	153
159	Tanzania (United Republic of)	135	161	169	166	152	145	143	143	143
159	Comoros	133	145	149	148	155	161	164	164	164
161	Mauritania	125	143	153	158	163	165	167	167	167
162	Lesotho	109	121	132	160	173	177	177	177	177
163	Senegal	145	160	158	168	161	153	151	151	151
164	Uganda	149	167	176	172	165	159	156	155	155
165	Benin	150	169	177	173	171	168	169	170	170
166	Sudan	119	142	152	152	154	156	158	159	159

Table M. Country rank per year for Probability of dying between birth and exactly age 5

HDI Rank	Countries	1980	1985	1990	2000	2005	2010	2011	2012	2013
166	Togo	134	152	160	165	167	171	173	174	174
168	Haiti	140	155	161	151	153	192	163	163	163
169	Afghanistan	160	175	174	167	169	175	176	176	176
170	Djibouti	121	141	147	154	160	164	165	166	166
171	Côte d'Ivoire	128	149	165	170	178	181	183	183	183
172	Gambia	156	170	170	161	156	157	159	160	160
173	Ethiopia	158	178	181	171	166	155	154	154	154
174	Malawi	162	182	188	183	172	163	161	156	156
175	Liberia	157	180	189	184	170	162	162	162	162
176	Mali	167	186	191	191	189	187	188	186	186
177	Guinea-Bissau	153	174	182	182	183	185	186	187	187
178	Mozambique	161	181	186	177	179	172	172	172	172
179	Guinea	164	183	187	181	181	178	179	179	179
180	Burundi	152	163	168	175	180	179	180	181	181
181	Burkina Faso	159	177	180	187	186	182	182	180	180
182	Eritrea	139	158	163	144	142	140	139	139	139
183	Sierra Leone	163	184	192	193	193	193	193	193	193
184	Chad	155	179	183	189	191	190	191	191	191
185	Central African Republic	137	159	171	176	184	186	187	188	188
186	Congo (Democratic Republic of the)	144	165	172	179	187	188	189	189	189
187	Niger	166	187	193	192	190	183	184	184	184
	San Marino			29	22	11	11	10	10	10
	Monaco			11	7	12	12	13	13	13
	Korea (Democratic People's Rep. of)		66	94	127	114	116	117	117	117
	Tuvalu	77	99	111	115	118	119	119	119	119
	Nauru			112	116	119	126	127	127	127
	Marshall Islands	75	91	99	114	120	127	128	128	128
	South Sudan	165	185	190	185	182	180	181	182	182
	Somalia		168	175	180	188	189	190	190	190
	**Total..**	**167**	**187**	**193**	**193**	**193**	**193**	**193**	**193**	**193**
	**Normalized rank**	0.210	0.123	0.140	0.197	0.223	0.233	0.228	0.233	0.233

International Human Development Indicators
Accessed: 7/14/2014,2:06 PM from: http://hdr.undp.org
Adolescent birth rate (women aged 15-19 years) (births per 1,000 women ages 15-19)
Number of births to women ages 15–19 per 1,000 women ages 15–19.
Source: UNDESA (2013a).2012 Revision. World Population Prospects. Population Division Database. Detailed Indicators. Accessed June, 14.2013. http//:esa.un.org/unpd/wpp/unpp/
Data in the tables are those available to the Human Development Report Office as of 15 November, 2013, unless otherwise specified.

Table N. Adolescent birth rate (women aged 15–19 years) (births per 1,000 women ages 15–19)

HDI Rank	Countries	1985	1990	2000	2005	2010
1	Norway	16.9	17.8	12.8	10	8.9
2	Australia	26.1	21.1	19.2	16.7	16
3	Switzerland	8.8	6.3	5.7	5.3	4.5
4	Netherlands	8	6.9	6.2	7.1	5.3
5	United States	51.6	53.3	51.3	43.2	39.7
6	Germany	22.5	17	13.1	12.4	9.8
7	New Zealand	32.8	31.9	29.9	26.7	30.1
8	Canada	24.9	23.2	20.1	15	13.8
9	Singapore	11	8	7.4	7.5	6
10	Denmark	12.1	9.2	8	6.6	5.9
11	Ireland	19	15	19	19	16.5
12	Sweden	12.7	11.8	7.2	6.4	6
13	Iceland	43.9	30.9	23.5	17.2	14.3
14	United Kingdom	27	32	31	27	25.8
15	Korea (Republic of)	12.3	6.5	3	2.2	2.1
15	Hong Kong, China (SAR)	10	6	5	3.8	3.1
17	Japan	4.2	4	4.4	5.8	5.1
19	Israel	31.3	21.9	17.7	16.1	14
20	France	20.1	13.7	9.8	10.8	10
21	Luxembourg	14.1	11.4	10.2	11.7	9.6
21	Belgium	16.2	11.4	10.2	11	10.4
21	Austria	30.1	21.4	14.8	13.3	11.1
24	Finland	16.6	12.7	9.5	10.6	9.2
25	Slovenia	42.1	26.2	7	4.8	4.8
26	Italy	16.8	10.3	7	7	6.8
27	Spain	21.2	15.1	8.1	10.3	12.7
28	Czech Republic	53.9	51.3	16.7	11.4	11.1
29	Greece	44.8	29.1	11.6	10.5	11.7
30	Brunei Darussalam	50.6	38	28.8	26.2	25.1
31	Qatar	71.5	57.7	22.8	19	15.1
32	Cyprus	38.3	34.6	13.1	7.2	6.6
33	Estonia	41.3	44.5	30.4	21.9	23
34	Saudi Arabia	118.1	84.7	37.3	20.6	11.6
35	Poland	34.1	32.4	20.4	14.9	15

Table N. Adolescent birth rate (women aged 15–19 years) (births per 1,000 women ages 15–19)

HDI Rank	Countries	1985	1990	2000	2005	2010
35	Lithuania	20.5	30.2	31.8	21.1	18.9
37	Slovakia	48.6	51	27.4	20.7	20.7
39	Malta	16.7	11.3	16.5	16.2	18.7
40	United Arab Emirates	116.8	74	27.6	25.6	29.9
41	Portugal	37.9	28	20.7	20.2	16.8
41	Chile	64.3	64	66.9	61.6	58.5
43	Hungary	59	46.7	25.6	21	19.6
44	Bahrain	51.6	32.5	18.3	16.9	14.7
44	Cuba	85.7	85.7	67.9	50.3	45.2
46	Kuwait	67.6	32	26.6	19.5	15.1
47	Croatia	22.7	21.3	17.8	14.4	13.6
48	Latvia	40.4	44.9	21.9	17.3	18.2
49	Argentina	74.2	73.4	69.8	60.7	56.9
50	Uruguay	62.6	66.4	67.3	63.5	61.1
51	Montenegro	29.7	26.9	23.4	20.9	18.6
51	Bahamas	85.6	69.8	61.9	44	31.8
53	Belarus	35.6	42.7	33.2	23.9	22.6
54	Romania	60.4	60.3	37.4	29.9	30.9
55	Libya	14.4	11	4.8	3.7	3.1
56	Oman	117.2	93.9	43.5	21.1	13.6
57	Russian Federation	45.3	49.2	35.1	27.6	29.3
58	Bulgaria	79.8	75.8	48.2	41.7	42.1
59	Barbados	70.8	49.9	52	50.5	49.4
61	Antigua and Barbuda	61.6	59.6	68.7	62.8	55.5
62	Malaysia	28.2	19.4	15	13	12.8
63	Mauritius	41.4	44.7	36.3	36.5	33.1
64	Trinidad and Tobago	85.2	72.1	44.3	38.4	38.1
65	Lebanon	60.5	51	28.3	19.3	12.5
65	Panama	111.1	102.6	95.9	91.1	84.6
67	Venezuela (Bolivarian Republic of)	101	103.7	94.1	92.1	89.9
68	Costa Rica	97.9	100.2	87.8	76.4	65.6
69	Turkey	86.3	71.1	52.4	44.8	39.3
70	Kazakhstan	38.2	46.2	42.1	29.1	28.1
71	Seychelles	84.3	67.6	57.9	57.9	59.9
71	Mexico	95.1	85.7	81.7	74.2	69.3
73	Sri Lanka	38.6	33.4	29	27.6	22.3
75	Iran (Islamic Republic of)	141	111.6	48.3	33.6	31.5
76	Azerbaijan	17.7	23.7	41.4	34.3	40.5
77	Serbia	43.9	42.2	28	23.3	19.3
77	Jordan	68	56.8	40.6	33.1	29.4
79	Grenada	100.6	99.2	61.5	51.2	42.4

Table N. Adolescent birth rate (women aged 15–19 years) (births per 1,000 women ages 15–19)

HDI Rank	Countries	1985	1990	2000	2005	2010
79	Georgia	76.3	76.1	60.5	48.5	50.9
79	Brazil	75	80.4	89.7	86	75.6
82	Peru	74.1	72	70.5	61.5	54.7
83	Ukraine	55.5	65.8	43	29.5	30.8
84	The former Yugoslav Republic of Macedonia	46.4	42.8	33.6	26.6	22.3
84	Belize	151.2	131.6	106.3	91.2	78.7
86	Bosnia and Herzegovina	42.4	40.5	27	17.5	17
87	Armenia	45.5	65	49.9	33.8	28
88	Fiji	58.6	63.3	45.8	41.5	45.2
89	Thailand	57.7	53.2	45.7	41.9	40.6
90	Tunisia	33.5	23.8	8.4	6.5	5.7
91	China	19.5	24.7	5.9	7.5	8.4
91	Saint Vincent and the Grenadines	110.4	95.1	76.1	64.6	58.9
93	Algeria	53	33.5	14.9	9.7	9.7
95	Albania	48.1	44	36.6	30.1	19.5
96	Jamaica	129.2	112.8	93.5	85.4	77.3
97	Saint Lucia	148.6	120.8	69.8	62	61.4
98	Colombia	80.6	77.2	91.7	95.7	74.3
98	Ecuador	93.2	89.1	83.9	85	83.5
100	Tonga	30.1	25.5	22.5	21.3	22.3
100	Suriname	81.4	60	50.8	45.2	39.5
102	Dominican Republic	110.6	110.2	111	109.6	108.7
103	Maldives	210.1	175.8	58.1	24.9	11
103	Mongolia	38.6	33.9	31.4	21.6	19
103	Turkmenistan	21.1	22.3	25.4	24.4	20.9
106	Samoa	46.6	39.4	45.1	37	31.7
107	Palestine, State of	97	95.1	97.5	72	50.1
108	Indonesia	96.3	72.9	47.8	51	51.5
109	Botswana	143.9	127.2	73.3	61.2	52.1
110	Egypt	111.5	80.3	58.8	52.5	48.7
111	Paraguay	98.3	91.6	91.9	82.3	72.3
112	Gabon	170.5	166.2	142	126.7	115
113	Bolivia (Plurinational State of)	89	89.4	86.9	84.4	78.2
114	Moldova (Republic of)	37	48.6	58.1	37.4	33.8
115	El Salvador	143.4	128.6	111.5	91.9	82.7
116	Uzbekistan	32.9	40.5	50	50	48.8
117	Philippines	51.6	50.1	49.1	53.9	54.1
118	Syrian Arab Republic	100.8	82.1	64	53.7	44.6
118	South Africa	93.6	95.4	80.6	70.7	59.2
120	Iraq	83.7	75.1	64.8	66.9	74.1
121	Viet Nam	21.2	26.6	28.9	28.5	31.7

Table N. Adolescent birth rate (women aged 15–19 years) (births per 1,000 women ages 15–19)

HDI Rank	Countries	1985	1990	2000	2005	2010
121	Guyana	80.7	63.4	76.8	90	101
123	Cabo Verde	107.3	109	105.8	94.7	81.6
124	Micronesia (Federated States of)	63.6	53.5	44.8	34.3	25.4
125	Kyrgyzstan	42.3	49.9	63.1	34.4	29.8
125	Guatemala	138.5	131.4	121.1	115.4	107.2
127	Namibia	130.6	116.8	90.4	81.7	74.4
128	Timor-Leste	56.4	54.5	71.9	70.1	65.8
129	Morocco	82.8	59.6	44	34	32.7
129	Honduras	140	133.5	114.6	102.5	93.1
131	Vanuatu	83.6	77.7	62	54.7	47.7
132	Nicaragua	154	160.1	132.6	119.4	112.7
133	Kiribati	53	48.3	41.6	30.1	22.2
133	Tajikistan	56.4	56.4	50.2	40.9	44.8
135	India	113.3	112.9	88.8	72.2	50.6
136	Cambodia	89.3	88.1	50.2	48.4	47
136	Bhutan	108.1	110.5	90.8	69.5	50.9
138	Ghana	128.3	118	87.5	77	67.6
139	Lao People's Democratic Republic	111.6	110	86.6	71.4	75.1
140	Congo	139.1	133.8	133.8	137.9	130.5
141	Zambia	97.4	93.3	141.9	157	139.7
142	Sao Tome and Principe	131	118.8	94.6	85	76.4
142	Bangladesh	192.7	176.9	130.3	107.5	88.7
144	Equatorial Guinea	132.1	134.4	133.9	128.8	122.9
145	Nepal	127.6	134	129.3	114	94.8
146	Pakistan	102.9	93.8	58.8	40.5	30.9
147	Kenya	163.1	147.8	105.7	104.2	100.2
148	Swaziland	149.1	141.8	109.4	102.4	88
149	Angola	229	229	215.1	204.7	192.3
150	Myanmar	62.5	44.4	23.7	19.3	16.2
151	Rwanda	75.9	71.9	55.2	44.7	42.8
152	Nigeria	165.7	154.9	136.6	130	123.9
152	Cameroon	197.3	191.5	162.6	151.1	130.7
154	Yemen	166.6	154.7	116.3	82.7	56.4
155	Madagascar	142.6	150.7	155.1	149.5	134.3
156	Zimbabwe	129	108.3	110.1	80.6	72.6
157	Papua New Guinea	95.5	79.6	75.6	71	66.9
157	Solomon Islands	110.3	98.9	71.1	70.1	70.3
159	Comoros	93.1	79.2	66.5	63.6	58
159	Tanzania (United Republic of)	150.8	146.4	133.3	132	130.4
161	Mauritania	111.2	110.4	101.3	92.2	82.5
162	Lesotho	77.4	77.7	94	89.6	90

Table N. Adolescent birth rate (women aged 15–19 years) (births per 1,000 women ages 15–19)

HDI Rank	Countries	1985	1990	2000	2005	2010
163	Senegal	167	146.8	111.7	104.2	98.1
164	Uganda	181.8	185.5	191	172.5	149.9
165	Benin	112.1	123.1	122.8	115.7	105.1
166	Togo	138.4	127.1	95.8	90.7	90.1
166	Sudan	112.4	101.5	110.5	118.9	106.6
168	Haiti	86.1	78.3	61.8	52.5	46.4
169	Afghanistan	159.8	165.7	170.1	150.5	117.5
170	Djibouti	63.2	67.2	29.3	26.9	22.1
171	Côte d'Ivoire	193.4	164.1	128.1	125.2	125.6
172	Gambia	201.3	179.2	133.6	117.2	110.9
173	Ethiopia	121.8	119.4	117.5	105.2	90
174	Malawi	202	148	167	157.4	157.2
175	Liberia	196.8	182.1	152.1	147.4	142
176	Mali	189.9	192.7	189.1	185.1	180.9
177	Guinea-Bissau	122.5	132.1	134.5	128.7	123.5
178	Mozambique	123.7	119.4	117	183.2	162.5
179	Guinea	179.3	181.5	173.7	164.2	154
180	Burundi	49	50.1	47.4	36.6	33.5
181	Burkina Faso	160.4	150.2	142.6	137.1	129.7
182	Eritrea	115.1	113.1	106.5	92	76.9
183	Sierra Leone	167.1	148.3	128.1	127.1	119
184	Chad	218	218.1	216.4	209.7	181.9
185	Central African Republic	164.3	156.4	134.4	122.5	106.6
186	Congo (Democratic Republic of the)	141.1	138.1	132.1	130.1	132
187	Niger	225.3	225	222.2	216.1	209.6
..	Korea (Democratic People's Rep. of)	5.2	3.2	1.2	1	0.6
..	South Sudan	141.9	141.2	127.8	118.3	95.4
..	Somalia	54	76.7	126.6	126.9	127.2

Table O. Country rank per year for number of births to women ages 15–19 per 1,000 women ages 15–19

HDI Rank	Countries	1985	1990	2000	2005	2010
1	Norway	16	20	21	17	16
2	Australia	28	22	33	33	40
3	Switzerland	4	4	6	6	5
4	Netherlands	3	6	8	12	8
5	United States	65	73	89	88	86
6	Germany	25	19	22	25	20
7	New Zealand	35	39	55	60	71

Table O. Country rank per year for number of births to women ages 15–19 per 1,000 women ages 15–19

HDI Rank	Countries	1985	1990	2000	2005	2010
8	Canada	27	27	34	30	33
9	Singapore	6	7	12	15	12
10	Denmark	7	8	13	10	10
11	Ireland	18	17	32	38	42
12	Sweden	9	14	11	8	11
13	Iceland	52	38	41	35	35
14	United Kingdom	29	40	57	62	64
15	Korea (Republic of)	8	5	2	2	2
15	Hong Kong, China (SAR)	5	3	5	4	4
17	Japan	1	2	3	7	7
19	Israel	34	25	29	31	34
20	France	20	16	17	21	21
21	Luxembourg	10	12	18	24	18
21	Belgium	12	13	19	22	22
21	Austria	32	24	24	27	25
24	Finland	13	15	16	20	17
25	Slovenia	49	32	10	5	6
26	Italy	15	9	9	11	14
27	Spain	23	18	14	18	29
28	Czech Republic	68	71	28	23	24
29	Greece	54	36	20	19	27
30	Brunei Darussalam	62	48	51	58	62
31	Qatar	87	78	39	39	38
32	Cyprus	43	47	23	13	13
33	Estonia	47	57	56	52	61
34	Saudi Arabia	138	112	65	44	26
35	Poland	38	42	35	29	37
35	Lithuania	21	37	59	48	48
37	Slovakia	60	69	47	45	53
39	Malta	14	11	27	32	47
40	United Arab Emirates	136	98	48	57	70
41	Portugal	41	35	36	43	43
41	Chile	83	85	107	109	114
43	Hungary	75	61	44	47	52
44	Bahrain	63	43	31	34	36
44	Cuba	104	113	109	96	95
46	Kuwait	84	41	45	42	39
47	Croatia	26	23	30	28	31
48	Latvia	46	59	37	36	45
49	Argentina	89	97	111	106	112

Table O. Country rank per year for number of births to women ages 15–19 per 1,000 women ages 15–19

HDI Rank	Countries	1985	1990	2000	2005	2010
50	Uruguay	80	88	108	112	118
51	Montenegro	31	34	40	46	46
51	Bahamas	103	91	101	89	78
53	Belarus	39	53	60	54	60
54	Romania	76	82	66	68	73
55	Libya	11	10	4	3	3
56	Oman	137	121	72	49	32
57	Russian Federation	55	64	62	64	67
58	Bulgaria	94	100	81	86	89
59	Barbados	86	66	90	97	101
61	Antigua and Barbuda	78	79	110	111	110
62	Malaysia	30	21	26	26	30
63	Mauritius	48	58	63	78	80
64	Trinidad and Tobago	102	95	74	82	83
65	Lebanon	77	70	50	41	28
65	Panama	128	129	138	139	142
67	Venezuela (Bolivarian Republic of)	121	130	135	143	145
68	Costa Rica	117	127	126	125	120
69	Turkey	106	92	91	91	84
70	Kazakhstan	42	60	70	66	66
71	Seychelles	101	90	93	105	117
71	Mexico	112	114	121	124	124
73	Sri Lanka	45	44	53	63	59
75	Iran (Islamic Republic of)	153	137	82	72	75
76	Azerbaijan	17	28	68	75	87
77	Serbia	53	52	49	53	50
77	Jordan	85	77	67	71	68
79	Grenada	119	126	99	99	90
79	Georgia	92	101	98	94	104
79	Brazil	90	110	128	135	132
82	Peru	88	94	113	108	109
83	Ukraine	70	87	71	67	72
84	The former Yugoslav Republic of Macedonia	57	54	61	59	58
84	Belize	162	152	143	140	137
86	Bosnia and Herzegovina	51	51	46	37	44
87	Armenia	56	86	84	73	65
88	Fiji	74	83	78	85	94
89	Thailand	73	72	77	87	88
90	Tunisia	37	29	15	9	9
91	China	19	30	7	14	15

Table O. Country rank per year for number of births to women ages 15–19 per 1,000 women ages 15–19

HDI Rank	Countries	1985	1990	2000	2005	2010
91	Saint Vincent and the Grenadines	126	123	118	114	115
93	Algeria	66	45	25	16	19
95	Albania	59	55	64	69	51
96	Jamaica	145	138	133	134	135
97	Saint Lucia	159	146	112	110	119
98	Colombia	95	103	131	146	129
98	Ecuador	110	116	122	132	141
100	Tonga	33	31	38	50	57
100	Suriname	97	81	88	92	85
102	Dominican Republic	127	134	148	153	159
103	Maldives	182	175	95	56	23
103	Mongolia	44	46	58	51	49
103	Turkmenistan	22	26	43	55	54
106	Samoa	58	49	76	80	77
107	Palestine, State of	115	122	139	122	102
108	Indonesia	114	96	80	98	106
109	Botswana	158	149	116	107	107
110	Egypt	130	109	96	100	99
111	Paraguay	118	118	132	129	126
112	Gabon	172	174	173	163	162
113	Bolivia (Plurinational State of)	107	117	124	131	136
114	Moldova (Republic of)	40	63	94	81	82
115	El Salvador	157	150	149	141	140
116	Uzbekistan	36	50	85	95	100
117	Philippines	64	68	83	103	108
118	Syrian Arab Republic	120	111	104	102	92
118	South Africa	111	124	120	119	116
120	Iraq	100	99	105	115	128
121	Viet Nam	24	33	52	65	76
121	Guyana	96	84	119	137	154
123	Cabo Verde	123	132	142	145	138
124	Micronesia (Federated States of)	82	74	75	76	63
125	Kyrgyzstan	50	65	103	77	69
125	Guatemala	150	151	155	155	158
127	Namibia	146	141	129	128	130
128	Timor-Leste	72	75	115	118	121
129	Morocco	98	80	73	74	79
129	Honduras	152	154	151	148	149
131	Vanuatu	99	105	102	104	98
132	Nicaragua	163	171	164	160	161

Table O. Country rank per year for number of births to women ages 15–19 per 1,000 women ages 15–19

HDI Rank	Countries	1985	1990	2000	2005	2010
133	Kiribati	67	62	69	70	56
133	Tajikistan	71	76	86	84	93
135	India	134	139	127	123	103
136	Cambodia	108	115	87	93	97
136	Bhutan	124	136	130	116	105
138	Ghana	143	142	125	126	123
139	Lao People's Democratic Republic	131	133	123	121	131
140	Congo	151	155	167	172	172
141	Zambia	116	119	172	177	176
142	Sao Tome and Principe	147	143	136	133	133
142	Bangladesh	176	176	162	152	144
144	Equatorial Guinea	148	157	168	167	165
145	Nepal	142	156	161	154	150
146	Pakistan	122	120	97	83	74
147	Kenya	166	163	141	149	153
148	Swaziland	160	160	145	147	143
149	Angola	185	185	183	183	184
150	Myanmar	79	56	42	40	41
151	Rwanda	91	93	92	90	91
152	Nigeria	168	169	171	168	167
152	Cameroon	179	181	177	176	173
154	Yemen	169	168	152	130	111
155	Madagascar	156	167	176	174	175
156	Zimbabwe	144	131	146	127	127
157	Papua New Guinea	113	108	117	120	122
157	Solomon Islands	125	125	114	117	125
159	Comoros	109	107	106	113	113
159	Tanzania (United Republic of)	161	161	165	170	171
161	Mauritania	129	135	140	144	139
162	Lesotho	93	104	134	136	146
163	Senegal	170	162	150	150	152
164	Uganda	174	180	182	180	178
165	Benin	132	147	156	156	155
166	Togo	149	148	137	138	148
166	Sudan	133	128	147	159	156
168	Haiti	105	106	100	101	96
169	Afghanistan	164	173	179	175	163
170	Djibouti	81	89	54	61	55
171	Côte d'Ivoire	177	172	160	162	168
172	Gambia	180	177	166	157	160

Table O. Country rank per year for number of births to women ages 15–19 per 1,000 women ages 15–19

HDI Rank	Countries	1985	1990	2000	2005	2010
173	Ethiopia	139	144	154	151	147
174	Malawi	181	164	178	178	180
175	Liberia	178	179	175	173	177
176	Mali	175	182	181	182	182
177	Guinea-Bissau	140	153	170	166	166
178	Mozambique	141	145	153	181	181
179	Guinea	173	178	180	179	179
180	Burundi	61	67	79	79	81
181	Burkina Faso	165	166	174	171	170
182	Eritrea	135	140	144	142	134
183	Sierra Leone	171	165	159	165	164
184	Chad	183	183	184	184	183
185	Central African Republic	167	170	169	161	157
186	Congo (Democratic Republic of the)	154	158	163	169	174
187	Niger	184	184	185	185	185
..	Korea (Democratic People's Rep. of)	2	1	1	1	1
..	South Sudan	155	159	158	158	151
..	Somalia	69	102	157	164	169
	**Total ....**	**185**	**185**	**185**	**185**	**185**
	**Normalized rank**	0.157	0.157	0.157	0.157	0.157

International Human Development Indicators
Accessed: 7/14/2014,2:08 PM from: http://hdr.undp.org
Maternal mortality ratio (deaths of women per100,000 live births)
Ratio of the number of maternal deaths to the number of live births in a given year, expressed per 100,000 live births.
Source: UN Maternal Mortality Estimation Group (MMEIG) WHO, UNICEF, UNFPA and the World Bank (2013). June 2012 updates. Accessed June, 2013. http://www.childinfo.org/maternal_mortality_ratio.php
Data in the tables are those available to the Human Development Report Office as of 15 November, 2013, unless otherwise specified.

Table P. Maternal mortality ratio (deaths of women per100,000 live births)

HDI Rank	Countries	1990	2000	2005	2010
1	Norway	9	8	9	7
2	Australia	10	9	7	7
3	Switzerland	7	6	6	8
4	Netherlands	10	13	8	6
5	United States	12	14	18	21
6	Germany	13	7	7	7
7	New Zealand	18	12	15	15
8	Canada	6	7	11	12
9	Singapore	6	15	9	3
10	Denmark	13	8	7	12
11	Ireland	6	6	2	6
12	Sweden	6	5	4	4
13	Iceland	8	7	6	5
14	United Kingdom	10	12	13	12
15	Korea (Republic of)	18	19	17	16
17	Japan	12	10	7	5
19	Israel	12	9	7	7
20	France	13	10	8	8
21	Luxembourg	6	11	17	20
21	Austria	10	5	5	4
21	Belgium	10	9	8	8
24	Finland	7	5	6	5
25	Slovenia	11	12	14	12
26	Italy	10	4	5	4
27	Spain	7	5	6	6
28	Czech Republic	15	7	7	5
29	Greece	6	5	3	3
30	Brunei Darussalam	29	24	25	24
31	Qatar	15	11	9	7
32	Cyprus	17	15	12	10
33	Estonia	48	28	23	2
34	Saudi Arabia	44	27	25	24
35	Poland	17	8	5	5

Table P. Maternal mortality ratio (deaths of women per100,000 live births)

HDI Rank	Countries	1990	2000	2005	2010
35	Lithuania	34	21	11	8
37	Slovakia	15	13	6	6
39	Malta	14	12	10	8
40	United Arab Emirates	24	14	13	12
41	Portugal	15	8	8	8
41	Chile	56	29	26	25
43	Hungary	23	10	10	21
44	Bahrain	23	22	21	20
44	Cuba	63	63	67	73
46	Kuwait	11	9	8	14
47	Croatia	8	11	14	17
48	Latvia	57	43	21	34
49	Argentina	71	63	69	77
50	Uruguay	39	35	31	29
51	Montenegro	8	11	9	8
51	Bahamas	52	56	47	47
53	Belarus	37	31	20	4
54	Romania	170	52	30	27
55	Libya	99	67	61	58
56	Oman	110	51	39	32
57	Russian Federation	74	57	37	34
58	Bulgaria	24	28	13	11
59	Barbados	120	49	41	51
62	Malaysia	53	39	34	29
63	Mauritius	68	28	32	60
64	Trinidad and Tobago	86	59	59	46
65	Lebanon	52	38	31	25
65	Panama	100	110	100	92
67	Venezuela (Bolivarian Republic of)	94	91	94	92
68	Costa Rica	38	47	50	40
69	Turkey	67	39	28	20
70	Kazakhstan	92	70	50	51
71	Mexico	92	82	54	50
73	Sri Lanka	85	58	44	35
75	Iran (Islamic Republic of)	120	48	30	21
76	Azerbaijan	56	65	52	43
77	Serbia	23	12	10	12
77	Jordan	110	79	72	63
79	Grenada	34	27	25	24
79	Georgia	63	58	61	67
79	Brazil	120	81	67	56

Table P. Maternal mortality ratio (deaths of women per100,000 live births)

HDI Rank	Countries	1990	2000	2005	2010
82	Peru	200	120	90	67
83	Ukraine	49	35	25	32
84	The former Yugoslav Republic of Macedonia	16	15	10	10
84	Belize	71	100	77	53
86	Bosnia and Herzegovina	18	10	9	8
87	Armenia	46	38	34	30
88	Fiji	32	31	29	26
89	Thailand	54	66	54	48
90	Tunisia	130	84	68	56
91	Saint Vincent and the Grenadines	59	88	64	48
91	China	120	61	45	37
93	Algeria	220	140	110	97
95	Albania	48	39	31	27
96	Jamaica	59	83	89	110
97	Saint Lucia	64	46	40	35
98	Colombia	170	130	100	92
98	Ecuador	180	130	110	110
100	Tonga	67	87	100	110
100	Suriname	84	130	110	130
102	Dominican Republic	220	130	130	150
103	Turkmenistan	82	91	76	67
103	Mongolia	120	96	84	63
103	Maldives	830	190	94	60
106	Samoa	260	150	120	100
107	Palestine, State of	90	64	67	64
108	Indonesia	600	340	270	220
109	Botswana	140	350	330	160
110	Egypt	230	100	78	66
111	Paraguay	120	110	110	99
112	Gabon	270	270	260	230
113	Bolivia (Plurinational State of)	450	280	240	190
114	Moldova (Republic of)	62	39	25	41
115	El Salvador	150	110	94	81
116	Uzbekistan	59	33	32	28
117	Philippines	170	120	110	99
118	Syrian Arab Republic	240	120	89	70
118	South Africa	250	330	360	300
120	Iraq	89	78	74	63
121	Guyana	180	220	280	280
121	Viet Nam	240	100	74	59
123	Cabo Verde	200	170	110	79

Table P. Maternal mortality ratio (deaths of women per100,000 live births)

HDI Rank	Countries	1990	2000	2005	2010
124	Micronesia (Federated States of)	140	130	110	100
125	Kyrgyzstan	73	82	77	71
125	Guatemala	160	130	120	120
127	Namibia	200	280	310	200
128	Timor-Leste	1000	610	410	300
129	Honduras	220	160	130	100
129	Morocco	300	170	130	100
131	Vanuatu	220	120	110	110
132	Nicaragua	170	130	110	95
133	Tajikistan	94	120	79	65
135	India	600	390	280	200
136	Cambodia	830	510	340	250
136	Bhutan	1000	430	270	180
138	Ghana	580	550	440	350
139	Lao People's Democratic Republic	1600	870	650	470
140	Congo	420	540	550	560
141	Zambia	470	540	500	440
142	Sao Tome and Principe	150	110	87	70
142	Bangladesh	800	400	330	240
144	Equatorial Guinea	1200	450	270	240
145	Nepal	770	360	250	170
146	Pakistan	490	380	310	260
147	Kenya	400	490	450	360
148	Swaziland	300	360	420	320
149	Angola	1200	890	650	450
150	Myanmar	520	300	230	200
151	Rwanda	910	840	550	340
152	Cameroon	670	730	720	690
152	Nigeria	1100	970	820	630
154	Yemen	610	380	270	200
155	Madagascar	640	400	310	240
156	Zimbabwe	450	640	690	570
157	Solomon Islands	150	120	110	93
157	Papua New Guinea	390	310	270	230
159	Comoros	440	340	310	280
159	Tanzania (United Republic of)	870	730	610	460
161	Mauritania	760	630	560	510
162	Lesotho	520	690	720	620
163	Senegal	670	500	430	370
164	Uganda	600	530	420	310
165	Benin	770	530	430	350

Table P. Maternal mortality ratio (deaths of women per100,000 live births)

HDI Rank	Countries	1990	2000	2005	2010
166	Togo	620	440	370	300
166	Sudan	1000	870	800	730
168	Haiti	620	460	410	350
169	Afghanistan	1300	1000	710	460
170	Djibouti	290	290	220	200
171	Côte d'Ivoire	710	590	510	400
172	Gambia	700	520	430	360
173	Ethiopia	950	700	510	350
174	Malawi	1100	840	630	460
175	Liberia	1200	1300	1100	770
176	Mali	1100	740	620	540
177	Guinea-Bissau	1100	970	890	790
178	Mozambique	910	710	630	490
179	Guinea	1200	970	800	610
180	Burundi	1100	1000	910	800
181	Burkina Faso	700	450	370	300
182	Eritrea	880	390	300	240
183	Sierra Leone	1300	1300	1000	890
184	Chad	920	1100	1100	1100
185	Central African Republic	930	1000	1000	890
186	Congo (Democratic Republic of the)	930	770	660	540
187	Niger	1200	870	720	590
	Korea (Democratic People's Rep. of)	97	120	85	81
	Somalia	890	1000	1000	1000

Table Q. Country rank per year for maternal mortality ratio

HDI Rank	Countries	1990	2000	2005	2010
1	Norway	13	13	23	17
2	Australia	14	17	12	17
3	Switzerland	7	7	7	22
4	Netherlands	14	34	18	13
5	United States	22	36	43	46
6	Germany	25	9	12	17
7	New Zealand	36	29	40	40
8	Canada	1	9	32	33
9	Singapore	1	38	23	2
10	Denmark	25	13	12	33
11	Ireland	1	7	1	13
12	Sweden	1	2	3	4

Table Q. Country rank per year for maternal mortality ratio

HDI Rank	Countries	1990	2000	2005	2010
13	Iceland	10	9	7	8
14	United Kingdom	14	29	35	33
15	Korea (Republic of)	36	41	41	41
17	Japan	22	21	12	8
19	Israel	22	17	12	17
20	France	25	21	18	22
21	Luxembourg	14	2	4	4
21	Austria	14	17	18	22
21	Belgium	1	25	41	43
24	Finland	7	2	7	8
25	Slovenia	20	29	38	33
26	Italy	14	1	4	4
27	Spain	7	2	7	13
28	Czech Republic	29	9	12	8
29	Greece	1	2	2	2
30	Brunei Darussalam	44	44	48	49
31	Qatar	29	25	23	17
32	Cyprus	34	38	34	30
33	Estonia	53	47	47	1
34	Saudi Arabia	51	45	48	49
35	Poland	46	42	32	22
35	Lithuania	34	13	4	8
37	Slovakia	29	34	7	13
39	Malta	28	29	28	22
40	United Arab Emirates	42	36	35	33
41	Portugal	60	50	53	52
41	Chile	29	13	18	22
43	Hungary	39	21	28	46
44	Bahrain	39	43	45	43
44	Cuba	67	75	81	97
46	Kuwait	20	17	18	39
47	Croatia	10	25	38	42
48	Latvia	62	62	45	63
49	Argentina	73	75	85	98
50	Uruguay	50	54	58	58
51	Montenegro	56	69	71	72
51	Bahamas	10	25	23	22
53	Belarus	48	51	44	4
54	Romania	105	68	56	55
55	Libya	88	80	78	81
56	Oman	90	67	66	61

Table Q. Country rank per year for maternal mortality ratio

HDI Rank	Countries	1990	2000	2005	2010
57	Russian Federation	76	70	65	63
58	Bulgaria	42	47	35	32
59	Barbados	92	66	68	76
62	Malaysia	58	58	63	58
63	Mauritius	72	47	61	83
64	Trinidad and Tobago	80	73	77	71
65	Lebanon	56	56	58	52
65	Panama	89	97	103	102
67	Venezuela (Bolivarian Republic of)	85	91	100	102
68	Costa Rica	49	64	72	68
69	Turkey	70	58	54	43
70	Kazakhstan	83	81	72	76
71	Mexico	83	85	75	75
73	Sri Lanka	79	71	69	65
75	Iran (Islamic Republic of)	92	65	56	46
76	Azerbaijan	60	78	74	70
77	Serbia	90	83	86	85
77	Jordan	39	29	28	33
79	Grenada	92	84	81	79
79	Georgia	67	71	78	91
79	Brazil	46	45	48	49
82	Peru	111	101	99	91
83	Ukraine	55	54	48	61
84	The former Yugoslav Republic of Macedonia	73	94	90	78
84	Belize	33	38	28	30
86	Bosnia and Herzegovina	36	21	23	22
87	Armenia	52	56	63	60
88	Fiji	45	51	55	54
89	Thailand	59	79	75	73
90	Tunisia	98	88	84	79
91	Saint Vincent and the Grenadines	92	74	70	67
91	China	63	90	80	73
93	Algeria	114	115	106	107
95	Albania	53	58	58	55
96	Jamaica	63	87	97	114
97	Saint Lucia	69	63	67	65
98	Colombia	105	108	103	102
98	Ecuador	109	108	106	114
100	Tonga	78	108	106	119
100	Suriname	70	89	103	114
102	Dominican Republic	114	108	118	120

Table Q. Country rank per year for maternal mortality ratio

HDI Rank	Countries	1990	2000	2005	2010
103	Turkmenistan	154	120	100	83
103	Mongolia	92	93	94	85
103	Maldives	77	91	89	91
106	Samoa	122	116	116	110
107	Palestine, State of	82	77	81	88
108	Indonesia	138	129	126	130
109	Botswana	99	131	138	121
110	Egypt	118	94	92	90
111	Paraguay	92	97	106	108
112	Gabon	123	122	125	131
113	Bolivia (Plurinational State of)	131	123	123	124
114	Moldova (Republic of)	66	58	48	69
115	El Salvador	101	97	100	100
116	Uzbekistan	63	53	61	57
117	Philippines	105	101	106	108
118	Syrian Arab Republic	121	128	141	141
118	South Africa	119	101	97	94
120	Iraq	81	82	87	85
121	Guyana	109	121	131	139
121	Viet Nam	119	94	87	82
123	Cabo Verde	111	118	106	99
124	Micronesia (Federated States of)	99	108	106	110
125	Kyrgyzstan	104	108	116	118
125	Guatemala	75	85	90	96
127	Namibia	111	123	134	125
128	Timor-Leste	165	155	144	141
129	Honduras	114	117	118	110
129	Morocco	125	118	118	110
131	Vanuatu	114	101	106	114
132	Nicaragua	105	108	106	106
133	Tajikistan	85	101	93	89
135	India	138	136	131	125
136	Cambodia	165	140	126	123
136	Bhutan	154	147	140	137
138	Ghana	137	153	151	148
139	Lao People's Democratic Republic	180	167	163	161
140	Congo	129	151	156	166
141	Zambia	133	151	153	156
142	Sao Tome and Principe	153	138	138	133
142	Bangladesh	101	97	96	94
144	Equatorial Guinea	173	142	126	133

Table Q. Country rank per year for maternal mortality ratio

HDI Rank	Countries	1990	2000	2005	2010
145	Nepal	151	132	124	122
146	Pakistan	134	134	134	138
147	Kenya	128	145	152	152
148	Swaziland	125	132	146	146
149	Angola	173	170	163	157
150	Myanmar	135	126	122	125
151	Rwanda	159	165	156	147
152	Cameroon	145	161	168	172
152	Nigeria	168	171	173	171
154	Yemen	141	134	126	125
155	Madagascar	144	138	134	133
156	Zimbabwe	131	157	166	167
157	Solomon Islands	127	127	126	131
157	Papua New Guinea	101	101	106	105
159	Comoros	130	129	134	139
159	Tanzania (United Republic of)	156	161	159	158
161	Mauritania	150	156	158	163
162	Lesotho	135	158	168	170
163	Senegal	145	146	148	154
164	Uganda	138	149	146	145
165	Benin	151	149	148	148
166	Togo	165	167	171	173
166	Sudan	142	141	142	141
168	Haiti	142	144	144	148
169	Afghanistan	178	174	167	158
170	Djibouti	124	125	121	125
171	Côte d'Ivoire	149	154	154	155
172	Gambia	147	148	148	152
173	Ethiopia	164	159	154	148
174	Malawi	168	165	161	158
175	Liberia	173	179	179	174
176	Mali	168	163	160	164
177	Guinea-Bissau	168	171	174	175
178	Mozambique	159	160	161	162
179	Guinea	173	171	171	169
180	Burundi	168	174	175	176
181	Burkina Faso	147	142	142	141
182	Eritrea	157	136	133	133
183	Sierra Leone	178	179	176	177
184	Chad	161	178	179	180
185	Central African Republic	162	174	176	177

Table Q. Country rank per year for maternal mortality ratio

HDI Rank	Countries	1990	2000	2005	2010
186	Congo (Democratic Republic of the)	162	164	165	164
187	Niger	173	167	168	168
	Korea (Democratic People's Rep. of)	87	101	95	100
	Somalia	158	174	176	179
	**Total ....**	**180**	**180**	**180**	**180**
	**Normalized rank**	0.200	0.233	0.256	0.261

International Human Development Indicators
Accessed: 7/14/2014,2:07 PM from: http://hdr.undp.org
Expenditure on health, total (% of GDP) (%)
Current and capital spending on health from government (central and local) budgets, external borrowing and grants (including donations from international agencies and nongovernmental organizations) and social (or compulsory) health insurance funds, expressed as a percentage of GDP.
Source: World Bank (2013). "World Development Indicators 2013." Washington, D.C.: World Bank. http://data.worldbank.org. Accessed October, 2013. http://data.worldbank.org/data-catalog/world-development-indicators
Data in the tables are those available to the Human Development Report Office as of 15 November, 2013, unless otherwise specified.

Table R. Current and capital spending on health
(from government - central and local - budgets, external borrowing and grants - including donations from international agencies and nongovernmental organizations - and social - or compulsory - health insurance funds, expressed as a percentage of GDP)

HDI Rank	Countries	2000	2005	2006	2007	2008	2009	2010	2011
1	Norway	167	161	157	158	148	158	154	150
2	Australia	161	154	154	153	154	142	144	147
3	Switzerland	179	180	175	172	172	172	173	175
4	Netherlands	160	170	168	178	178	180	182	181
5	United States	186	184	185	184	186	185	187	186
6	Germany	182	179	177	177	175	177	179	176
7	New Zealand	150	153	158	154	164	162	161	164
8	Canada	171	172	171	168	171	175	176	178
9	Singapore	12	29	31	25	45	51	41	43
10	Denmark	169	171	169	167	169	176	175	177
11	Ireland	113	131	131	132	152	152	151	154
12	Sweden	163	164	159	159	161	160	159	156
13	Iceland	177	167	163	162	160	157	153	149
14	United Kingdom	137	144	150	150	150	159	158	153
15	Korea (Republic of)	55	82	88	98	101	102	110	108
17	Japan	149	146	143	145	147	153	152	152
19	Israel	146	135	133	133	132	122	125	122
20	France	181	182	180	181	179	179	180	180
21	Luxembourg	147	138	134	124	122	130	132	124
21	Belgium	162	174	166	165	168	169	169	169
21	Austria	180	178	173	174	174	173	174	170
24	Finland	143	152	149	143	142	148	147	146
25	Slovenia	165	149	146	139	141	147	146	148
26	Italy	159	160	161	156	159	155	155	158
27	Spain	144	148	151	151	156	156	157	155
28	Czech Republic	124	114	114	107	110	127	121	117
29	Greece	156	168	167	166	177	174	172	173
30	Brunei Darussalam	16	8	7	7	9	9	11	7
31	Qatar	6	13	10	6	3	5	3	2

Table R. Current and capital spending on health
(from government - central and local - budgets, external borrowing and grants - including donations from international agencies and nongovernmental organizations - and social - or compulsory - health insurance funds, expressed as a percentage of GDP)

HDI Rank	Countries	2000	2005	2006	2007	2008	2009	2010	2011
32	Cyprus	102	99	98	94	113	112	115	116
33	Estonia	81	62	62	65	90	104	94	84
34	Saudi Arabia	49	21	24	23	14	24	25	20
35	Lithuania	127	86	92	97	103	117	109	99
35	Poland	91	94	93	99	114	113	108	102
37	Andorra	117	85	89	96	96	85	112	109
37	Slovakia	92	117	128	137	136	146	145	142
39	Malta	131	162	162	155	140	135	138	141
40	United Arab Emirates	7	3	5	8	13	30	22	14
41	Chile	151	105	94	105	119	119	117	118
41	Portugal	173	177	172	169	170	170	170	168
43	Hungary	141	151	148	136	129	121	127	123
44	Bahrain	37	27	22	22	35	36	33	24
44	Cuba	114	140	135	175	176	178	164	162
46	Kuwait	10	5	6	4	2	20	7	10
47	Croatia	152	122	121	128	134	124	129	125
48	Latvia	106	100	117	119	107	100	102	91
49	Argentina	172	150	147	146	143	151	135	131
50	Uruguay	185	147	145	142	139	136	134	130
51	Bahamas	82	90	119	122	127	118	120	120
51	Montenegro	148	157	144	138	137	150	148	151
53	Belarus	111	112	99	103	89	80	68	64
54	Romania	51	77	65	66	67	62	78	74
55	Libya	33	6	3	5	5	12	13	37
56	Oman	17	9	8	9	7	8	8	4
57	Russian Federation	89	68	71	70	57	82	95	90
58	Bulgaria	118	128	120	111	118	114	124	114
59	Barbados	125	120	124	120	120	92	99	119
60	Palau	183	181	179	180	180	171	178	171
61	Antigua and Barbuda	73	66	61	41	50	44	76	77
62	Malaysia	18	22	29	27	27	38	38	17
63	Mauritius	31	48	58	64	65	67	85	79
64	Trinidad and Tobago	38	81	63	52	42	88	92	73
65	Lebanon	178	142	139	144	128	109	88	93
65	Panama	153	129	123	108	126	129	140	132
67	Venezuela (Bolivarian Republic of)	97	75	81	88	79	72	58	56
68	Costa Rica	140	133	137	149	163	166	165	174
69	Turkey	77	73	84	89	93	96	100	100
70	Kazakhstan	44	36	32	17	30	35	32	26

Table R. Current and capital spending on health
(from government - central and local - budgets, external borrowing and grants - including donations from international agencies and nongovernmental organizations - and social - or compulsory - health insurance funds, expressed as a percentage of GDP)

HDI Rank	Countries	2000	2005	2006	2007	2008	2009	2010	2011
71	Seychelles	70	33	27	16	15	10	14	21
71	Mexico	80	87	82	82	84	89	93	89
73	Sri Lanka	32	35	38	28	19	14	17	15
73	Saint Kitts and Nevis	65	26	45	35	29	21	42	40
75	Iran (Islamic Republic of)	59	83	78	75	82	74	59	80
76	Azerbaijan	67	136	97	63	43	75	60	57
77	Jordan	176	158	140	148	153	154	136	135
77	Serbia	145	163	164	176	173	168	168	167
79	Grenada	129	92	102	95	88	84	75	88
79	Brazil	142	145	153	152	144	140	143	145
79	Georgia	134	155	152	147	158	165	163	161
82	Peru	66	50	49	60	80	57	48	46
83	Ukraine	94	97	103	104	104	123	128	112
84	Belize	40	49	48	54	58	65	73	71
84	The former Yugoslav Republic of Macedonia	170	141	138	115	105	94	98	98
86	Bosnia and Herzegovina	139	156	156	157	157	164	162	166
87	Armenia	121	61	41	32	28	39	40	36
88	Fiji	35	23	25	24	21	29	29	23
89	Thailand	25	20	19	21	33	27	24	30
90	Tunisia	87	79	77	77	76	71	70	87
91	Saint Vincent and the Grenadines	34	25	30	30	49	53	44	49
91	China	57	53	50	44	47	52	50	54
93	Algeria	26	15	15	18	24	37	34	27
93	Dominica	76	59	60	57	52	45	77	78
95	Albania	126	95	91	93	92	87	83	92
96	Jamaica	90	34	37	48	62	49	54	50
97	Saint Lucia	101	98	110	127	130	132	126	111
98	Colombia	104	91	106	110	111	106	97	86
98	Ecuador	45	123	129	131	123	131	133	115
100	Tonga	72	103	74	109	99	42	49	59
100	Suriname	168	110	95	83	85	78	71	65
102	Dominican Republic	122	71	68	59	63	59	62	67
103	Turkmenistan	36	14	4	3	1	3	5	9
103	Mongolia	68	70	56	67	81	73	64	62
103	Maldives	136	121	115	102	133	126	89	137
106	Samoa	107	64	66	73	75	60	90	107
108	Indonesia	3	11	13	13	12	6	9	11
109	Botswana	63	125	96	125	44	43	51	52
110	Egypt	86	67	70	55	51	48	43	48

Table R. Current and capital spending on health
(from government - central and local - budgets, external borrowing and grants - including donations from international agencies and nongovernmental organizations - and social - or compulsory - health insurance funds, expressed as a percentage of GDP)

HDI Rank	Countries	2000	2005	2006	2007	2008	2009	2010	2011
111	Paraguay	174	106	111	100	97	133	156	160
112	Gabon	9	7	11	11	10	17	18	13
113	Bolivia (Plurinational State of)	110	80	57	50	53	70	63	51
114	Moldova (Republic of)	132	166	176	179	181	182	181	179
115	El Salvador	158	124	112	101	98	98	105	106
116	Uzbekistan	98	60	73	76	61	66	66	68
117	Philippines	19	19	18	29	31	26	27	31
118	Syrian Arab Republic	75	38	28	26	17	13	15	18
118	South Africa	164	159	155	140	138	139	141	139
120	Iraq	13	55	23	53	54	137	139	133
121	Guyana	100	76	42	34	108	91	69	76
121	Viet Nam	85	88	101	117	106	105	103	105
123	Cabo Verde	71	58	64	58	48	47	46	45
124	Micronesia (Federated States of)	154	183	183	182	184	183	183	183
125	Kyrgyzstan	69	84	113	112	95	97	101	95
125	Guatemala	93	104	127	126	116	108	106	101
127	Namibia	108	127	125	114	115	107	65	63
128	Timor-Leste	157	176	181	171	149	103	72	53
129	Morocco	42	65	67	74	69	63	79	82
129	Honduras	130	132	132	141	145	144	142	140
131	Vanuatu	29	17	17	47	55	54	56	33
132	Nicaragua	138	137	142	161	165	163	160	163
133	Tajikistan	58	56	59	68	74	76	80	75
133	Kiribati	155	169	174	183	183	181	171	165
135	India	50	39	34	31	32	22	21	25
136	Bhutan	133	42	46	56	59	41	35	32
136	Cambodia	123	119	79	45	66	86	82	72
138	Ghana	62	116	72	87	78	50	55	47
139	Lao People's Democratic Republic	22	40	43	37	36	15	6	12
140	Congo	4	4	9	10	8	2	4	5
141	Zambia	96	118	100	78	91	81	81	85
142	Bangladesh	11	16	16	19	18	18	20	19
142	Sao Tome and Principe	166	175	141	129	117	134	122	121
144	Equatorial Guinea	2	1	1	1	11	55	30	28
145	Nepal	88	93	86	62	73	61	53	66
146	Pakistan	15	12	12	12	16	7	10	6
147	Kenya	61	46	47	43	37	40	39	41
148	Swaziland	84	109	118	113	135	138	130	129
149	Angola	14	18	39	20	26	69	16	16

Table R. Current and capital spending on health
(from government - central and local - budgets, external borrowing and grants - including donations from international agencies and nongovernmental organizations - and social - or compulsory - health insurance funds, expressed as a percentage of GDP)

HDI Rank	Countries	2000	2005	2006	2007	2008	2009	2010	2011
150	Myanmar	5	2	2	2	4	1	1	3
151	Rwanda	43	102	170	164	162	161	167	172
152	Cameroon	53	52	53	49	56	46	52	55
152	Nigeria	56	107	87	116	100	99	61	61
154	Yemen	54	57	69	69	68	77	67	70
155	Madagascar	30	24	33	36	22	19	19	29
156	Zimbabwe	1							
157	Papua New Guinea	39	32	20	38	46	28	28	35
157	Solomon Islands	60	134	116	84	77	125	118	144
159	Comoros	28	44	52	61	64	23	57	60
159	Tanzania (United Republic of)	24	31	105	80	70	64	111	113
161	Mauritania	105	63	21	42	40	58	84	69
162	Lesotho	135	115	136	160	167	167	177	182
163	Senegal	48	72	83	79	83	68	74	81
164	Uganda	128	165	165	163	155	149	150	157
165	Benin	46	51	55	46	38	33	31	42
166	Togo	83	108	104	106	102	110	119	128
166	Sudan	21	30	54	86	112	111	113	134
168	Haiti	112	47	80	71	71	93	104	126
169	Afghanistan		101	107	130	131	128	166	159
170	Djibouti	99	126	122	134	124	120	131	127
171	Côte d'Ivoire	79	41	51	81	86	79	86	103
172	Gambia	27	43	44	33	34	32	37	39
173	Ethiopia	47	37	40	51	41	34	45	44
174	Malawi	109	143	160	121	151	141	137	136
175	Liberia	103	139	178	173	182	186	185	188
176	Mali	120	96	109	118	109	101	96	104
177	Guinea-Bissau	74	78	85	90	87	95	107	94
178	Mozambique	115	113	108	91	72	83	91	97
179	Guinea	95	74	76	92	94	90	87	83
180	Burundi	116	173	182	170	166	145	149	143
181	Burkina Faso	78	111	130	135	146	116	116	96
182	Eritrea	52	10	14	14	25	11	12	8
183	Sierra Leone	187	186	184	187	187	188	188	187
184	Chad	119	54	36	39	23	31	26	34
185	Central African Republic	41	45	35	40	39	16	23	22
186	Congo (Democratic Republic of the)	64	69	75	85	125	143	123	138
187	Niger	23	89	90	72	60	56	47	58
	South Sudan					6	4	2	1

Table R. Current and capital spending on health
(from government - central and local - budgets, external borrowing and grants - including donations from international agencies and nongovernmental organizations - and social - or compulsory - health insurance funds, expressed as a percentage of GDP)

HDI Rank	Countries	2000	2005	2006	2007	2008	2009	2010	2011
	Monaco	20	28	26	15	20	25	36	38
	San Marino	175	130	126	123	121	115	114	110
	Marshall Islands	188	185	186	185	188	187	186	184
	Tuvalu	184	187	187	186	185	184	184	185
	Somalia	8							

Table S. Country rank per year for total expenditure on health

HDI Rank	Countries	2000	2005	2006	2007	2008	2009	2010	2011
1	Norway	167	161	157	158	148	158	154	150
2	Australia	161	154	154	153	154	142	144	147
3	Switzerland	179	180	175	172	172	172	173	175
4	Netherlands	160	170	168	178	178	180	182	181
5	United States	186	184	185	184	186	185	187	186
6	Germany	182	179	177	177	175	177	179	176
7	New Zealand	150	153	158	154	164	162	161	164
8	Canada	171	172	171	168	171	175	176	178
9	Singapore	12	29	31	25	45	51	41	43
10	Denmark	169	171	169	167	169	176	175	177
11	Ireland	113	131	131	132	152	152	151	154
12	Sweden	163	164	159	159	161	160	159	156
13	Iceland	177	167	163	162	160	157	153	149
14	United Kingdom	137	144	150	150	150	159	158	153
15	Korea (Republic of)	55	82	88	98	101	102	110	108
17	Japan	149	146	143	145	147	153	152	152
19	Israel	146	135	133	133	132	122	125	122
20	France	181	182	180	181	179	179	180	180
21	Luxembourg	147	138	134	124	122	130	132	124
21	Belgium	162	174	166	165	168	169	169	169
21	Austria	180	178	173	174	174	173	174	170
24	Finland	143	152	149	143	142	148	147	146
25	Slovenia	165	149	146	139	141	147	146	148
26	Italy	159	160	161	156	159	155	155	158
27	Spain	144	148	151	151	156	156	157	155
28	Czech Republic	124	114	114	107	110	127	121	117
29	Greece	156	168	167	166	177	174	172	173
30	Brunei Darussalam	16	8	7	7	9	9	11	7
31	Qatar	6	13	10	6	3	5	3	2
32	Cyprus	102	99	98	94	113	112	115	116

Table S. Country rank per year for total expenditure on health

HDI Rank	Countries	2000	2005	2006	2007	2008	2009	2010	2011
33	Estonia	81	62	62	65	90	104	94	84
34	Saudi Arabia	49	21	24	23	14	24	25	20
35	Lithuania	127	86	92	97	103	117	109	99
35	Poland	91	94	93	99	114	113	108	102
37	Andorra	117	85	89	96	96	85	112	109
37	Slovakia	92	117	128	137	136	146	145	142
39	Malta	131	162	162	155	140	135	138	141
40	United Arab Emirates	7	3	5	8	13	30	22	14
41	Chile	151	105	94	105	119	119	117	118
41	Portugal	173	177	172	169	170	170	170	168
43	Hungary	141	151	148	136	129	121	127	123
44	Bahrain	37	27	22	22	35	36	33	24
44	Cuba	114	140	135	175	176	178	164	162
46	Kuwait	10	5	6	4	2	20	7	10
47	Croatia	152	122	121	128	134	124	129	125
48	Latvia	106	100	117	119	107	100	102	91
49	Argentina	172	150	147	146	143	151	135	131
50	Uruguay	185	147	145	142	139	136	134	130
51	Bahamas	82	90	119	122	127	118	120	120
51	Montenegro	148	157	144	138	137	150	148	151
53	Belarus	111	112	99	103	89	80	68	64
54	Romania	51	77	65	66	67	62	78	74
55	Libya	33	6	3	5	5	12	13	37
56	Oman	17	9	8	9	7	8	8	4
57	Russian Federation	89	68	71	70	57	82	95	90
58	Bulgaria	118	128	120	111	118	114	124	114
59	Barbados	125	120	124	120	120	92	99	119
60	Palau	183	181	179	180	180	171	178	171
61	Antigua and Barbuda	73	66	61	41	50	44	76	77
62	Malaysia	18	22	29	27	27	38	38	17
63	Mauritius	31	48	58	64	65	67	85	79
64	Trinidad and Tobago	38	81	63	52	42	88	92	73
65	Lebanon	178	142	139	144	128	109	88	93
65	Panama	153	129	123	108	126	129	140	132
67	Venezuela (Bolivarian Republic of)	97	75	81	88	79	72	58	56
68	Costa Rica	140	133	137	149	163	166	165	174
69	Turkey	77	73	84	89	93	96	100	100
70	Kazakhstan	44	36	32	17	30	35	32	26
71	Seychelles	70	33	27	16	15	10	14	21
71	Mexico	80	87	82	82	84	89	93	89
73	Sri Lanka	32	35	38	28	19	14	17	15

Table S. Country rank per year for total expenditure on health

HDI Rank	Countries	2000	2005	2006	2007	2008	2009	2010	2011
73	Saint Kitts and Nevis	65	26	45	35	29	21	42	40
75	Iran (Islamic Republic of)	59	83	78	75	82	74	59	80
76	Azerbaijan	67	136	97	63	43	75	60	57
77	Jordan	176	158	140	148	153	154	136	135
77	Serbia	145	163	164	176	173	168	168	167
79	Grenada	129	92	102	95	88	84	75	88
79	Brazil	142	145	153	152	144	140	143	145
79	Georgia	134	155	152	147	158	165	163	161
82	Peru	66	50	49	60	80	57	48	46
83	Ukraine	94	97	103	104	104	123	128	112
84	Belize	40	49	48	54	58	65	73	71
84	The former Yugoslav Republic of Macedonia	170	141	138	115	105	94	98	98
86	Bosnia and Herzegovina	139	156	156	157	157	164	162	166
87	Armenia	121	61	41	32	28	39	40	36
88	Fiji	35	23	25	24	21	29	29	23
89	Thailand	25	20	19	21	33	27	24	30
90	Tunisia	87	79	77	77	76	71	70	87
91	Saint Vincent and the Grenadines	34	25	30	30	49	53	44	49
91	China	57	53	50	44	47	52	50	54
93	Algeria	26	15	15	18	24	37	34	27
93	Dominica	76	59	60	57	52	45	77	78
95	Albania	126	95	91	93	92	87	83	92
96	Jamaica	90	34	37	48	62	49	54	50
97	Saint Lucia	101	98	110	127	130	132	126	111
98	Colombia	104	91	106	110	111	106	97	86
98	Ecuador	45	123	129	131	123	131	133	115
100	Tonga	72	103	74	109	99	42	49	59
100	Suriname	168	110	95	83	85	78	71	65
102	Dominican Republic	122	71	68	59	63	59	62	67
103	Turkmenistan	36	14	4	3	1	3	5	9
103	Mongolia	68	70	56	67	81	73	64	62
103	Maldives	136	121	115	102	133	126	89	137
106	Samoa	107	64	66	73	75	60	90	107
108	Indonesia	3	11	13	13	12	6	9	11
109	Botswana	63	125	96	125	44	43	51	52
110	Egypt	86	67	70	55	51	48	43	48
111	Paraguay	174	106	111	100	97	133	156	160
112	Gabon	9	7	11	11	10	17	18	13
113	Bolivia (Plurinational State of)	110	80	57	50	53	70	63	51
114	Moldova (Republic of)	132	166	176	179	181	182	181	179
115	El Salvador	158	124	112	101	98	98	105	106

Table S. Country rank per year for total expenditure on health

HDI Rank	Countries	2000	2005	2006	2007	2008	2009	2010	2011
116	Uzbekistan	98	60	73	76	61	66	66	68
117	Philippines	19	19	18	29	31	26	27	31
118	Syrian Arab Republic	75	38	28	26	17	13	15	18
118	South Africa	164	159	155	140	138	139	141	139
120	Iraq	13	55	23	53	54	137	139	133
121	Guyana	100	76	42	34	108	91	69	76
121	Viet Nam	85	88	101	117	106	105	103	105
123	Cabo Verde	71	58	64	58	48	47	46	45
124	Micronesia (Federated States of)	154	183	183	182	184	183	183	183
125	Kyrgyzstan	69	84	113	112	95	97	101	95
125	Guatemala	93	104	127	126	116	108	106	101
127	Namibia	108	127	125	114	115	107	65	63
128	Timor-Leste	157	176	181	171	149	103	72	53
129	Morocco	42	65	67	74	69	63	79	82
129	Honduras	130	132	132	141	145	144	142	140
131	Vanuatu	29	17	17	47	55	54	56	33
132	Nicaragua	138	137	142	161	165	163	160	163
133	Tajikistan	58	56	59	68	74	76	80	75
133	Kiribati	155	169	174	183	183	181	171	165
135	India	50	39	34	31	32	22	21	25
136	Bhutan	133	42	46	56	59	41	35	32
136	Cambodia	123	119	79	45	66	86	82	72
138	Ghana	62	116	72	87	78	50	55	47
139	Lao People's Democratic Republic	22	40	43	37	36	15	6	12
140	Congo	4	4	9	10	8	2	4	5
141	Zambia	96	118	100	78	91	81	81	85
142	Bangladesh	11	16	16	19	18	18	20	19
142	Sao Tome and Principe	166	175	141	129	117	134	122	121
144	Equatorial Guinea	2	1	1	1	11	55	30	28
145	Nepal	88	93	86	62	73	61	53	66
146	Pakistan	15	12	12	12	16	7	10	6
147	Kenya	61	46	47	43	37	40	39	41
148	Swaziland	84	109	118	113	135	138	130	129
149	Angola	14	18	39	20	26	69	16	16
150	Myanmar	5	2	2	2	4	1	1	3
151	Rwanda	43	102	170	164	162	161	167	172
152	Cameroon	53	52	53	49	56	46	52	55
152	Nigeria	56	107	87	116	100	99	61	61
154	Yemen	54	57	69	69	68	77	67	70
155	Madagascar	30	24	33	36	22	19	19	29
156	Zimbabwe	1							

Table S. Country rank per year for total expenditure on health

HDI Rank	Countries	2000	2005	2006	2007	2008	2009	2010	2011
157	Papua New Guinea	39	32	20	38	46	28	28	35
157	Solomon Islands	60	134	116	84	77	125	118	144
159	Comoros	28	44	52	61	64	23	57	60
159	Tanzania (United Republic of)	24	31	105	80	70	64	111	113
161	Mauritania	105	63	21	42	40	58	84	69
162	Lesotho	135	115	136	160	167	167	177	182
163	Senegal	48	72	83	79	83	68	74	81
164	Uganda	128	165	165	163	155	149	150	157
165	Benin	46	51	55	46	38	33	31	42
166	Togo	83	108	104	106	102	110	119	128
166	Sudan	21	30	54	86	112	111	113	134
168	Haiti	112	47	80	71	71	93	104	126
169	Afghanistan		101	107	130	131	128	166	159
170	Djibouti	99	126	122	134	124	120	131	127
171	Côte d'Ivoire	79	41	51	81	86	79	86	103
172	Gambia	27	43	44	33	34	32	37	39
173	Ethiopia	47	37	40	51	41	34	45	44
174	Malawi	109	143	160	121	151	141	137	136
175	Liberia	103	139	178	173	182	186	185	188
176	Mali	120	96	109	118	109	101	96	104
177	Guinea-Bissau	74	78	85	90	87	95	107	94
178	Mozambique	115	113	108	91	72	83	91	97
179	Guinea	95	74	76	92	94	90	87	83
180	Burundi	116	173	182	170	166	145	149	143
181	Burkina Faso	78	111	130	135	146	116	116	96
182	Eritrea	52	10	14	14	25	11	12	8
183	Sierra Leone	187	186	184	187	187	188	188	187
184	Chad	119	54	36	39	23	31	26	34
185	Central African Republic	41	45	35	40	39	16	23	22
186	Congo (Democratic Republic of the)	64	69	75	85	125	143	123	138
187	Niger	23	89	90	72	60	56	47	58
	South Sudan					6	4	2	1
	Monaco	20	28	26	15	20	25	36	38
	San Marino	175	130	126	123	121	115	114	110
	Marshall Islands	188	185	186	185	188	187	186	184
	Tuvalu	184	187	187	186	185	184	184	185
	Somalia	8							
	**Total ....**	**188**	**187**	**187**	**187**	**188**	**188**	**188**	**188**
	**Normalized rank**	0.160	0.128	0.176	0.193	0.106	0.090	0.101	0.154

Table T. Aid funding for Madagascar during the period 1988 to 2014 in millions of dollars
(* Data based on Repoblikan'I Madagasikara 2013 & http://stpca-primature.gov.mg ** data based on http://aiddata.org/dashboard#/advanced/project-list)

Year	Sources			
	Madagascar Government 2015*		AidData.org**	
	Total	Environment	Total	Environment
1988			969.5	12.9
1989			558.8	2.2
1990			738.3	67.3
1991			475.9	22.6
1992			315	58.6
1993			348.5	31.8
1994			492.6	17.8
1995			234.4	18.0
1996			401.6	56.6
1997			1100	81.3
1998			934.8	28.0
1999			938.1	14.0
2000	295.2		635.1	14.0
2001	229.6		1200	19.7
2002	325.7		557.6	23.1
2003	485.0		1000	10.5
2004	667.5		1500	90.4
2005	850.0		1800	16.6
2006	750.0		744.8	27.1
2007	758.5		1900	31.7
2008	700.0		1300	22.2
2009	442.8	16.6	917.9	16.6
2010	518.9	12.5	341.8	5.7
2011	442.7	15.8	350.4	16.2
2012	421.3	26.6	738.4	9.1
2013	509.5	19.5	969.5	12.9
2014	557.8	23.7	558.8	2.2

Table U. Madagascar native taxa assessed for the Red List
(IUCN Red List Web Site site@iucnredlist.org; EX = Extinct, CR = Critically Endangered, EN = Endangered, VU = Vulnerable, NT = Near Threatened, LC = Least Concern, LR = Lower Risk, DD = Data Deficient)

Red List status*	Native plants	Native animals	Total
EX		11	11
CR	169	82	251
EN	226	211	437
VU	191	292	483
NT	34	223	257
LC	354	1,679	2,033
LR	23	10	33
DD	39	377	416
Total	1,036	2,885	3,921
