# Supplementary material for: How Effective Have Thirty Years of Internationally Driven Conservation and Development Efforts Been in Madagascar?
Source: PLoS One. 2016 Aug 17;11(8):e0161115. doi: 10.1371/journal.pone.0161115 (PMC4988661; doi:10.1371/journal.pone.0161115)
Supplement: S2 File — Relative performance of Madagascar compared to Africa (Fig A). Evolution of HDI and MDGs 1 to 6 values over time for Madagascar, mean values for the World, Sub-Saharan Africa, and minimum and maximum values (Fig B). (PDF) [file pone.0161115.s003.pdf]

S2 File. MDG performance of Madagascar compared to the World, Africa, and Sub-Saharan Africa

Fig A. Relative performance of Madagascar compared to Africa 2

Fig B. Evolution of HDI and MDGs 1 to 6 values over time for Madagascar, mean values for the World, Sub-Saharan Africa, and minimum and maximum values 3

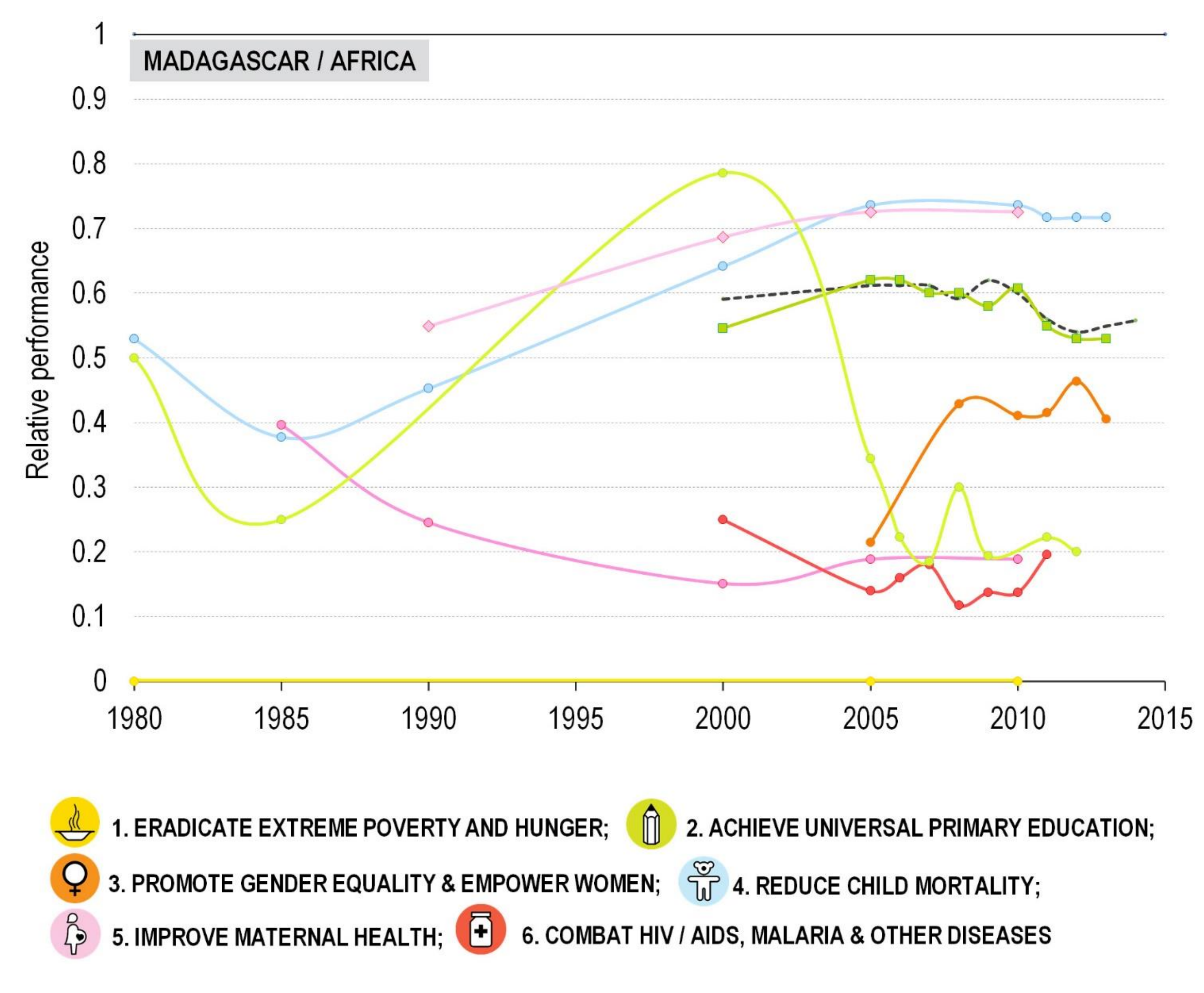

Fig A. Relative performance of Madagascar compared to Africa

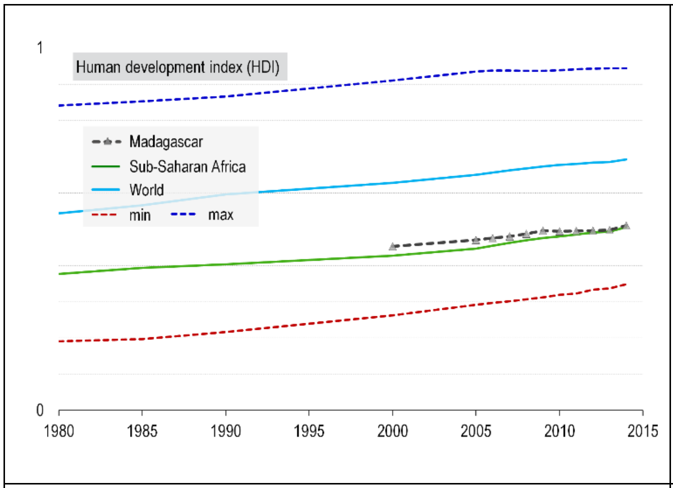
1
Human development index (HDI)
Madagascar
Sub-Saharan Africa
World
min
max
0
1980
1985
1990
1995
2000
2005
2010
2015

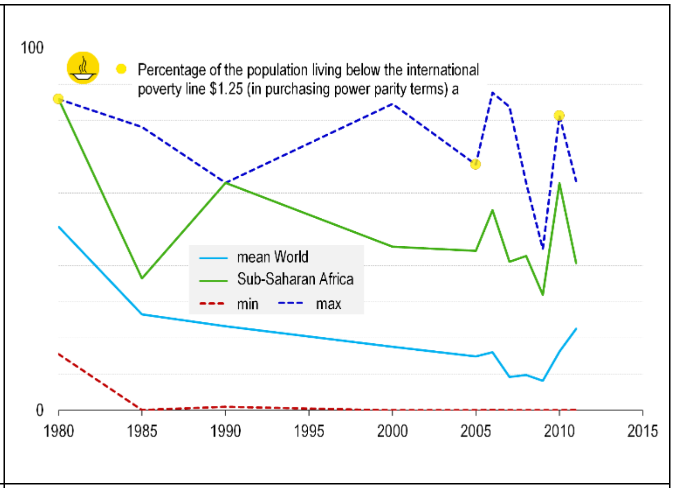
100
Percentage of the population living below the international poverty line $1.25 (in purchasing power parity terms) a
mean World
Sub-Saharan Africa
min
max
0
1980
1985
1990
1995
2000
2005
2010
2015

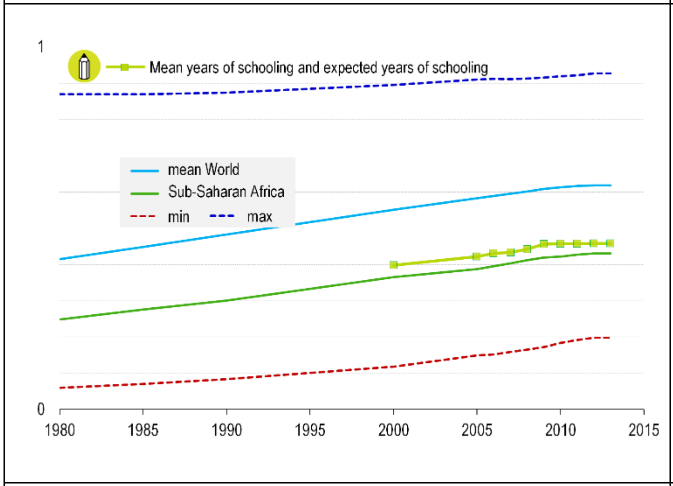
1
Mean years of schooling and expected years of schooling
mean World
Sub-Saharan Africa
min
max
0
1980
1985
1990
1995
2000
2005
2010
2015

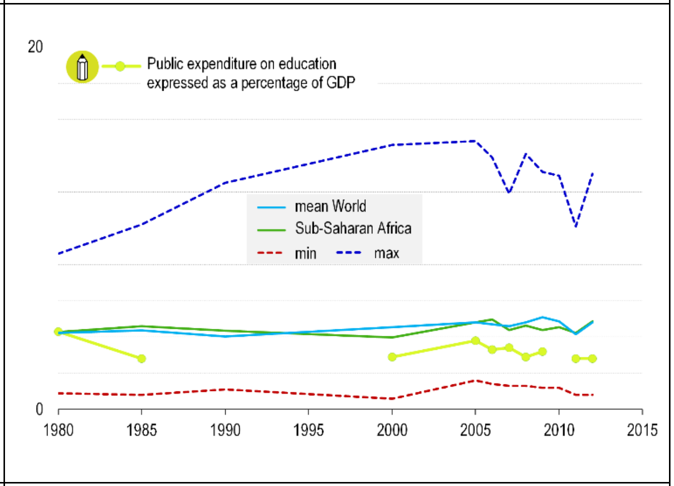
20
Public expenditure on education expressed as a percentage of GDP
mean World
Sub-Saharan Africa
min
max
0
1980
1985
1990
1995
2000
2005
2010
2015

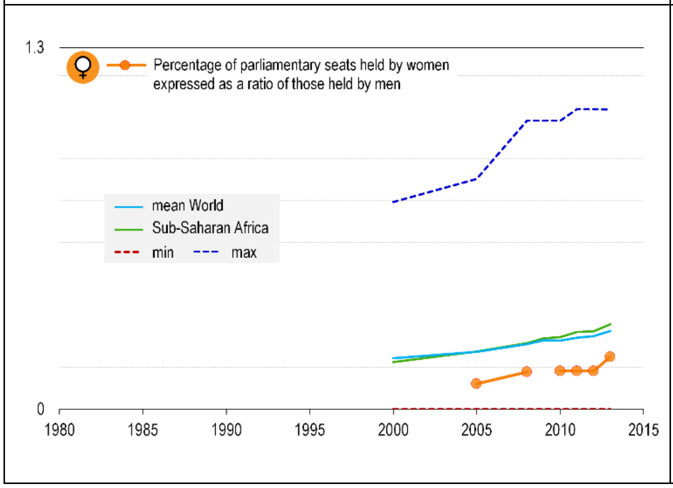
1.3
Percentage of parliamentary seats held by women expressed as a ratio of those held by men
mean World
Sub-Saharan Africa
min
max
0
1980
1985
1990
1995
2000
2005
2010
2015

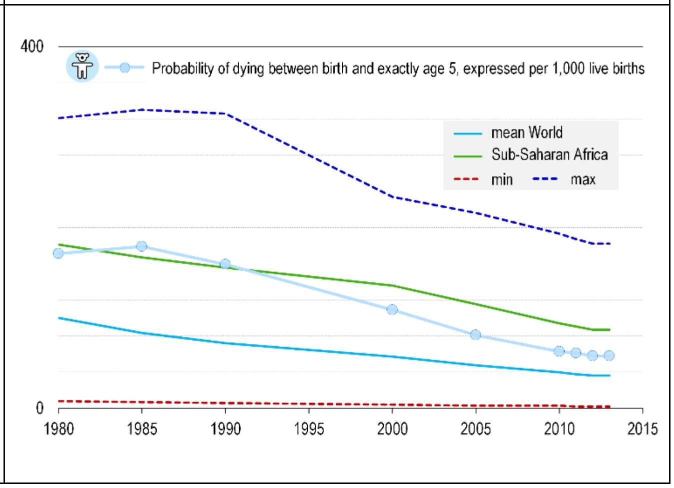
400
Probability of dying between birth and exactly age 5, expressed per 1,000 live births
mean World
Sub-Saharan Africa
min
max
0
1980
1985
1990
1995
2000
2005
2010
2015

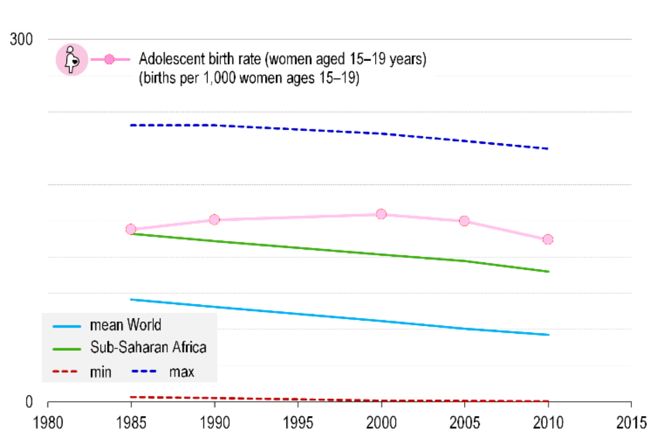

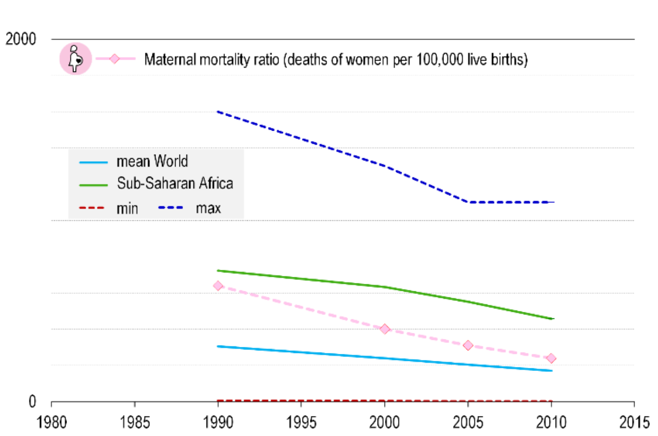

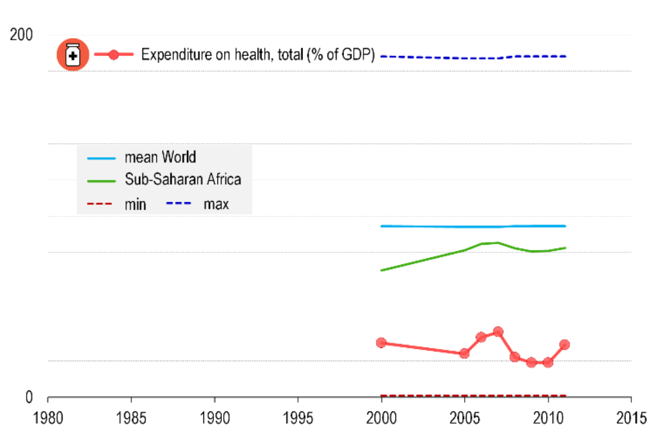

1. ERADICATE EXTREME POVERTY AND HUNGER;
2. ACHIEVE UNIVERSAL PRIMARY EDUCATION;
3. PROMOTE GENDER EQUALITY & EMPOWER WOMEN;
4. REDUCE CHILD MORTALITY; 5. IMPROVE MATERNAL HEALTH;
6. COMBAT HIV / AIDS, MALARIA & OTHER DISEASES

Fig B. Evolution of HDI and MDGs 1 to 6 values over time for Madagascar, mean values for the World, Sub-Saharan Africa, and minimum and maximum values
